# Surveillance of Parvovirus in Free-Roaming Dogs in the Qinling Mountains and Assessment of the Risk of Cross-Species Transmission to Giant Pandas

**DOI:** 10.3390/ani16111686

**Published:** 2026-05-31

**Authors:** Zhiyang Huang, U Cheong, Zichen Liu, Jiao He, Leigang Zhao, Dapeng Zhu, Haojie Xu, Yuhan Tsai, Jingjie Wei, Zhonghao Dan, Bolong Lu, Yipeng Jin

**Affiliations:** 1 College of Veterinary Medicine, China Agricultural University, Beijing 100193, China; 2 Foping National Nature Reserve Administration, Foping County, Hanzhong 723400, China

**Keywords:** canine parvovirus, free-roaming dogs, giant panda, cross-species transmission, vaccination coverage, seroepidemiology, home range overlap, wildlife disease

## Abstract

To assess the risk of cross-species transmission of canine parvovirus (CPV) from free-roaming dogs to wild giant pandas in the Foping National Nature Reserve, we collected rectal swabs and serum samples from dogs and fecal samples from giant pandas seasonally (August 2024–August 2025), combined with population surveys and GPS home-range tracking. Vaccination coverage declined from 54.2% to 36.4%, while the proportion of susceptible dogs rose from 8.7% to 29.5%. The CPV nucleic acid positive rate in dogs was 3.9% (4/102, all sub-adults, three deaths), whereas all giant panda samples were negative. Based on individual–seasonal exposure, the serum exposure rate was 30.4%, with immature dogs at significantly higher risk than adults (OR = 5.37). Home-range overlap between dogs and giant pandas (95% KDE: 19.17% in cold seasons vs. 2.61% in warm seasons) and encounter probability were markedly higher in winter and spring. Canine-derived CPV strains possessed the molecular potential to infect giant pandas. In summary, CPV persists long-term in free-roaming dog populations, summer vaccination has not established an effective immune barrier, and winter–spring is a high-risk window for cross-species transmission. We recommend enhanced winter–spring immunization, spatial control measures, and quarantine protocols for incoming dogs.

## Simple Summary

Canine parvovirus (CPV) is a highly contagious virus that can infect giant pandas and cause fatal disease. In the Foping National Nature Reserve (Shaanxi, China), free-roaming dogs live close to wild giant pandas. We tracked these dogs, collected samples, and used GPS collars to see if CPV could spread from dogs to pandas. We found that the virus stayed in the dog population for at least ten months. Vaccination coverage among dogs dropped from 54% to 36% during the study, while the number of unprotected young dogs increased. Four dogs tested positive for CPV, but no panda samples contained the virus. However, blood tests showed that 30% of dogs had been recently exposed, with young dogs at five times higher risk. During winter and spring, dog and panda home ranges overlapped much more than in summer, and the virus strain found in dogs had the molecular potential to infect pandas. Therefore, even though we did not find infected pandas, the risk of CPV spilling over from dogs to pandas remains real, especially during cold seasons. We recommend additional vaccinations in winter and spring, stronger dog control measures, and stopping the entry of outside dogs.

## 1. Introduction

Canine parvovirus (CPV-2) belongs to the genus Parvovirus within the family Parvoviridae and is a non-enveloped, single-stranded DNA virus. The virus is primarily transmitted via the fecal-oral route, and is highly contagious and can cause vomiting, diarrhea, leukopenia, and even death in animals. CPV has a broad host range, including carnivores such as Canidae, Felidae, Ursidae, and Mustelidae [1]. The giant panda (*Ailuropoda melanoleuca*) is one of the hosts of CPV [2,3], and a fatal CPV infection in a wild giant panda cub was reported in Sichuan Province [4], indicating that CPV poses a threat to the survival and reproduction of wild giant panda populations.

The Foping National Nature Reserve (FNNR) is located in the middle of the Qinling Mountains in Hanzhong City, Shaanxi Province. It serves as an important habitat for the Qinling subspecies of the giant panda (*Ailuropoda melanoleuca qinlingensis*) [5]. The reserve is home to 70–80 wild giant pandas, which is the highest population density of wild giant pandas in China [6]. There are free-roaming domestic dogs in and around the reserve. These dogs move freely and can easily enter the habitats of wild giant pandas. Previous studies have confirmed that free-roaming dogs can serve as sources and vectors for pathogens such as CPV, leading to widespread transmission of CPV between domestic dogs and wildlife [7,8,9]. Therefore, the FNNR high-density giant panda population and the active free-range dogs together constitute a potential cross-species transmission hotspot of the disease. Our previous research found that the home range of free-roaming dogs and wild giant pandas in the FNNR significantly overlap during winter and spring, and the canine-derived viruses have the risk of cross-species transmission [10], but that study primarily focused on CDV and did not systematically investigate CPV.

Vaccination is an effective method to prevent CPV. In order to prevent and control canine-borne infectious diseases, the FNNR has implemented summer intensive immunization of free-roaming dogs in collaboration with surrounding villages since 2017, along with sentinel dog surveillance and other control measures to establish herd immunity. A dog-banning campaign was also carried out for several months in 2019. However, systematic evaluations of CPV epidemiology and the effectiveness of the herd immunity barrier are still lacking, making it impossible to determine whether current strategies can effectively prevent the transmission of CPV from domestic dogs to giant pandas. To address these gaps, we systematically collected rectal swabs and serum samples from free-roaming dogs, as well as the fresh feces samples from giant pandas, on a seasonal basis from the summer of 2024 to the summer of 2025. Through pathogen detection and serum antibody analysis, we aimed to determine whether CPV persists long-term in free-roaming dog populations and whether current immunization strategies are sufficient to establish an effective herd immunity barrier. Furthermore, by integrating dog population dynamics and home-range analysis, we identified high-risk contact windows for CPV cross-species transmission between dogs and giant pandas, as well as their seasonal characteristics. In this study, longitudinal serological monitoring, pathogen molecular evolution analysis and a high-precision home range overlap model were combined for the first time to comprehensively evaluate the risk of cross-species transmission of CPV from domestic dogs to wild giant pandas. The results will provide key scientific evidence for optimizing dog management and CPV prevention and control in protected areas.

## 2. Materials and Methods

### 2.1. Study Design Overview

Five cross-sectional sampling and census time points were set:

T1: late August 2024

T2: late November–early December 2024

T3: mid-February 2025

T4: late April 2025

T5: mid-to-late August 2025

These five time points define four between-census intervals, which serve as the basic temporal units for all longitudinal analyses (population dynamics, serological exposure):

Interval 1: T1 → T2 (Sept 2024–late Nov 2024)

Interval 2: T2 → T3 (Dec 2024–mid-Feb 2025)

Interval 3: T3 → T4 (mid-Feb–late Apr 2025)

Interval 4: T4 → T5 (May–mid-to-late Aug 2025)

Study sites. Six locations were included: Daguping, Sanguanmiao, Liangfengya, Yueba, Longtanzi, and Xiaonanping. All except Xiaonanping are partially or entirely within the FNNR.

Free-roaming dogs. All known free-roaming dogs were individually identified by full-body photographs verified with owners and assigned a unique ID. At each of the five time points (T1–T5), we attempted to collect a rectal swab (or fresh fecal sample) and a blood sample from every available dog. Blood was collected only from dogs aged ≥ 4 months to avoid interference from maternal antibodies. Of the 102 dogs that were sampled at least once, 79 provided at least one blood sample and at least one pathogen sample, while the remaining 23 provided only pathogen samples. Intestinal tissue and contents were additionally collected from any dog that had recently died. Individual-level sampling details are provided in Supplementary S8. Pre-study-period samples (January–June 2024) from clinically affected dogs were used exclusively for phylogenetic comparison and were excluded from all epidemiological analyses.

Giant pandas. Fresh giant panda fecal samples were collected opportunistically by reserve staff during routine patrols within the core panda habitat (primarily in Daguping, Sanguanmiao, and Liangfengya), concurrently with the dog sampling at T1–T5.

GPS tracking. Six representative free-roaming dogs (one from each identified activity group) and five wild giant pandas were fitted with GPS collars. Tracking was conducted during a warm season (July–August, 2 months) and a cold season (December–March, 4 months), yielding 6527 dog locations and 8372 giant panda locations. These tracking periods correspond roughly to T1/T5 and Intervals 2–3, respectively.

Detailed methods for population monitoring, pathogen detection, serology, and spatial analysis are provided in the following sections.

### 2.2. Canine Population Dynamics and Immunological Monitoring

As described in the Study Design and Sampling Overview, all dogs were individually identified. For each dog, we recorded location, date of birth, sex, age, breed, owner, vaccination status, and place of origin. Vaccination history was jointly confirmed by the local vaccination coordinator and the dog owner, including information on vaccination date, location, and vaccine type. The origin of each dog was obtained through communication with the dog owner and was classified into four categories: a. Born locally; b. Other villages within the study area; c. Areas outside the study area but still under the jurisdiction of Foping County; d. Areas outside the jurisdiction of Foping County. If a dog moved out or died during the study period, the date of departure, method of departure, or date of death was recorded.

Using the five sampling time points as reference, vaccination coverage (the proportion of dogs aged ≥ 2 months with a history of vaccination) and the proportion of susceptible dogs (the proportion of unvaccinated dogs aged 2–12 months among all dogs) were calculated at each time point. Birth rate, mortality rate, immigration rate, and emigration rate were calculated for each of the four intervals (Interval 1–4). Each rate was calculated as the number of corresponding events during that interval divided by the average population size for that interval (arithmetic mean of the total population counts at the two bounding time points), with results expressed as percentages.

### 2.3. Pathogen Monitoring and Phylogenetic Analysis

Rectal swabs, fecal samples, and tissue samples from dead dogs were collected as described in the Study Design and Sampling Overview. Pre-study-period samples (January–June 2024) from clinically affected dogs were included only for phylogenetic comparison; they were not used in prevalence or exposure calculations.

After grinding, all samples were centrifuged at 12,000 rpm for 2 min. The supernatant was taken to extract viral DNA using the DNA Viral Genome Extraction Kit (Beijing Solarbio Science & Technology Co., Ltd., Beijing, China) according to the manufacturer’s instructions. The extracted DNA was detected by qPCR using the following primers: CPV-QF: 5′-CCATGGTACAGATCCAGATGA-3′, and CPV-QR: 5′-GCCTCAAAAGAATAATATGGT-3′. The reaction system was 20 μL: 10 μL Taq Pro Universal SYBR qPCR Master Mix (Nanjing Vazyme Biotech Co., Ltd., Nanjing, China), 0.5 μL each of the forward and reverse primers, 7 μL nuclease-free water, and 2 μL DNA template. The reaction program was: 95 °C for 30 s for initial denaturation; 40 cycles (10 s denaturation at 95 °C, 30 s annealing and extension at 60 °C); the melting curve program consists of 15 s at 95 °C, 1 min at 60 °C, 15 s at 95 °C, and 30 s at 50 °C. A result is considered positive if the Cq value is ≤35 and the melting curve exhibits a single peak at approximately 78 °C.

For qPCR-positive samples, the amplification, sequencing, and assembly of the full-length VP2 gene (1755 bp) were outsourced to a commercial sequencing company. The sequences obtained in this study were aligned with 44 reference sequences of different subtypes and from different countries and hosts downloaded from GenBank (see Supplementary S1 for details). Sequence alignment was performed using the Clustal W method in MEGA 10 software. A maximum-likelihood phylogenetic tree was constructed using the Tamura–Nei model with 1000 bootstrap replicates. The 17 key amino acid residues (80, 87, 93, 101, 103, 232, 267, 297, 300, 305, 323, 324, 370, 375, 426, 564, 568) of VP2 protein of CPV strains isolated in this study and 13 key reference strains of different hosts and subtypes (including 3 CPV strains from giant pandas) were compared and analyzed.

### 2.4. Detection of CPV Antibody (IgG) and Exposure Criteria

For serological exposure analysis, an exposure interval was defined as the period between two consecutive sampling time points for the same dog, corresponding to the four intervals defined in the Study Design and Sampling Overview: Interval 1 (T1 → T2), Interval 2 (T2 → T3), Interval 3 (T3 → T4), and Interval 4 (T4 → T5). Blood sample collection and age restrictions are described in the Study Design and Sampling Overview. Anti-CPV antibody titers were quantified using a canine trivalent virus fluorescent immunoassay (FIA) kit (Haiwei Te (Guangzhou) Medical Technology Co., Ltd., Guangzhou, China) according to the manufacturer’s instructions. Antibody titer grading criteria are provided in Supplementary S2. This testing method cannot distinguish between antibodies produced by vaccination and those produced by exposure to wild-type virus strains.

This study defines “exposure” as follows: (1) Past exposure: Antibody titers ≥ S2 in the first sample collected from dogs with no history of vaccination; the past exposure rate prior to the study period was derived from data collected before the large-scale vaccination campaign in August 2024. Since the specific timing of exposure could not be determined, these cases were excluded from subsequent analyses. (2) Recent exposure: For the same dog, no vaccination occurred between two consecutive sampling events, and antibody titers showed a significant increase; specific criteria are provided in Supplementary S2. (3) Clinical infection: qPCR-positive. Exposure records counted during the study period included only recent exposure and clinical infection. The time interval between two consecutive samples from the same dog was treated as a single exposure assessment unit. After excluding records where the exposure status could not be determined, a total of 194 valid records were obtained for analysis (Supplementary S3: Table S2). Since a single dog may have contributed multiple records, these records are not entirely independent. Each record included: dog ID, location (Daguping, Sanguanmiao, or outside the habitat), interval (Interval 1, Interval 2, Interval 3, or Interval 4), breed (mixed breed or purebred), sex (female or male), age group (puppy < 12 months or adult ≥ 12 months), vaccination history (yes or no), and exposure status (yes or no). Among these, the four locations of Liangfengya, Yueba, Longtanzi, and Xiaonanping were combined into “Peripheral Habitat” due to their small sample sizes and their locations along the highway connecting Foping County to FNNR, far from the core zone.

Chi-square tests were used to preliminarily screen for categorical variables significantly associated with exposure. Since some interval–location combinations had zero exposure, multivariate Firth logistic regression was used to analyze the effects of age group, interval, location, and their interaction terms on exposure; results are presented as odds ratios (OR) and 95% confidence intervals (CI). The reference group was set as adult dogs, Interval 4, and Daguping. All analyses were performed using the logistf package in R 4.5.2, with a significance level of α = 0.05.

### 2.5. Home Range Data Collection and Overlap Analysis

Free-roaming dogs from three locations where giant pandas are frequently active—Daguping, Sanguanmiao, and Liangfengya—were selected as the study subjects. Among these, the dogs in Sanguanmiao formed a single activity group, the dogs in Daguping were divided into four independent groups, and a single dog at the Liangfengya Conservation Station roamed alone. Since the home ranges of individuals within the same group overlap significantly, and to balance tracking costs with representativeness, one representative individual was selected from each group—confirmed by the owner as the most active, free of visible diseases or injuries, and exhibiting normal mobility during preliminary observations. A total of six dogs were fitted with Sinmao M1 GPS collars for tracking. Based on preliminary findings that dogs are rarely active at night, only daytime locations were recorded: the collars were set to record positions every 2 h between 6:00 and 18:00 daily, yielding 6 valid locations per day, with each record containing the date, time, longitude, and latitude. Tracking lasted for 6 months, covering the warm season (summer, July–August, 2 months) and the cold season (winter and spring, December–March, 4 months). A total of 6527 GPS locations were obtained for the dogs, with a location validity rate exceeding 99%; missing data were attributed to temporary signal obstruction. 8372 activity points from 5 wild giant pandas active in areas with frequent dog activity within the FNNR were obtained via GPS collars. The transmission frequency varied by individual and season.

Using ArcGIS 10.7 software, the 95% Minimum Convex Polygon (MCP) method and the 95% Kernel Density Estimation (KDE) method were applied to calculate the total home range area and overlap rate of the dogs and giant pandas during the warm and cold seasons, respectively, while the 50% KDE was used to calculate the core home range area and overlap rate. To avoid erroneously including blank areas between individuals in the total home range, the 95% MCP method first calculated individual home ranges, then took the union of all home ranges of individuals of the same species and season to form the total home range. The KDE method uses a weighted approach to correct for differences in sampling frequency: Although giant pandas are solitary, the sampling frequency varies across different individuals and seasons. Therefore, for each individual–seasonal group, the ratio of the theoretical maximum number of sampling points to the actual number of points is calculated as the weight for that individual’s sampling points. For free-roaming dogs, the sampling frequency is the same across different individuals and seasons; however, since each individual represents the activity of its respective pack, the weight is set to the number of individuals in that pack. Based on the degree of overlap between the home ranges calculated by the two methods, and in conjunction with relevant research literature, the KDE bandwidths for dogs [10] and giant pandas [11] were determined to be 200 m and 500 m, respectively.

Local villagers and staff members enter and exit the reserve on foot via a fixed patrol route, and free-roaming dogs often follow their owners along this route over long distances. To investigate the route selection preferences of dogs and giant pandas, we generated vector lines of the patrol routes using waypoints collected by handheld GPS, and defined the study area as the 95% KDE home range area of giant pandas during that season (area: $A_{total}$). We established concentric buffer zones with a 100 m stride centered on the patrol routes until the entire study area was covered, and then used the 95% KDE polygon to clip the area of each zone $A_{i}$. We weighted all animal locations within the season by sampling frequency or population size to obtain the total weighted count $U_{total}$, and recorded the weighted count within each zone as $U_{i}$. The calculation methods for the actual usage ratio $r_{i}$ and the available ratio $p_{i}$ are as follows:

$$r_{i}=\frac{U_{i}}{U_{\mathrm{total}}}$$

$$p_{i}=\frac{A_{i}}{A_{\mathrm{total}}}$$

Calculate the Jacobs selection index (D) for each ring to quantify the animals’ preference (D>0) or avoidance (D<0) for each ring [12].

$$D_{i}=\frac{r_{i}-p_{i}}{r_{i}+p_{i}-2r_{i}p_{i}}$$

Define the encounter probability index $M_{i}$ and its normalized form:

$$M_{i}=\frac{U_{panda,i}\times \;U_{dog,i}}{A_{i}}$$

$$M_{norm},i=\frac{M_{i}}{sum\left(M_{j}\right)}$$

where $U_{panda,i}$ and $U_{dog,i}$ represent the weighted point counts for giant pandas and dogs, respectively, within the i th zone. Since the 95% KDE areas for giant pandas differ between the cold and warm seasons, the maximum distance common to both seasons (rounded up to 100 m if smaller) is used to standardize the zone boundaries. Bootstrap analysis (500 iterations) was performed to calculate the 95% confidence intervals for the M of each zone and the seasonal differences $\Delta M=M_{warm}-M_{cold}$ along with their 95% confidence intervals. If the interval did not include 0, the seasonal difference for that zone was considered significant. All spatial analyses and statistical calculations were performed in R version 4.5.2.

## 3. Results

### 3.1. Population Dynamics and Vaccination Status of Dogs

A total of 107 free-roaming dogs were recorded during the study period. Vaccination coverage declined steadily from 54.2% in Interval 4 of 2024 to 36.4% in Interval 4 of 2025, while the proportion of susceptible dogs rose from 8.7% to 29.5% (Table 1). Birth peaks occurred in Interval 2 and Interval 4, while migration peaks occurred in Interval 2 and Interval 3. Among the newly arrived dogs, 60.3% came from outside Foping County, and the majority had no vaccination history or an unknown vaccination history.

Daguping has the largest dog population, and its vaccination coverage has consistently remained above the overall average; however, due to high birth rates and a large proportion of puppies and sub-adults, the proportion of susceptible dogs has consistently been higher than in other areas. The population at Sanguanmiao is stable (10–12 dogs), with vaccination coverage at 40–50%. The remaining four locations (Liangfengya, Yueba, Longtanzi, and Xiaonanping) have small populations (0–10 dogs) and generally low vaccination coverage (0–25%); however, due to the absence of sustained puppy births and inflows, the proportion of susceptible dogs has not shown significant peaks. For complete information, see Supplementary S4.

### 3.2. Pathogen Monitoring and Phylogenetic Analysis

**Figure 1:**
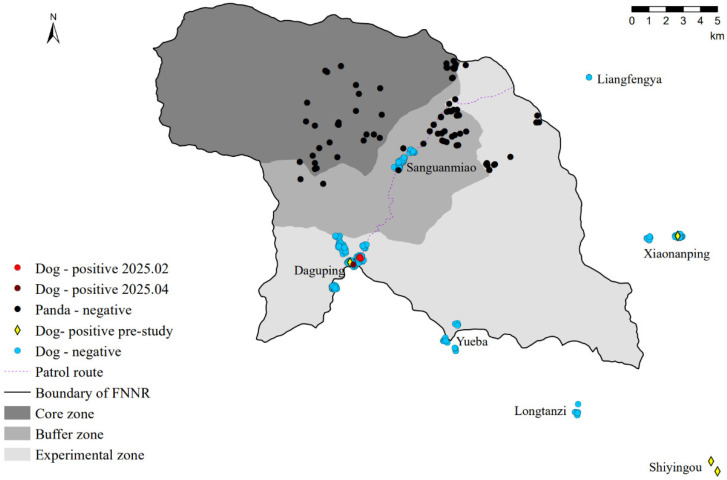
Sampling sites of domestic dogs and giant pandas. Light blue dots represent sampling sites of qPCR-negative dog samples collected during the study period. Black dots represent sampling sites of qPCR-negative giant panda fecal samples collected during the study period. Red and dark red dots represent sampling sites of PCR-positive dog samples collected during the study period. Yellow diamonds represent some of the sampling sites of CPV strains isolated in the first half of 2024, while sampling sites in Yangxian and Shiquan counties are not shown because they are far from FNNR.

As shown in Figure 1, a total of 302 samples—including dog feces, rectal swabs, and tissue samples—were collected from 102 free-roaming dogs. A total of four positive samples were detected, originating from four different individuals. Among these, three samples were collected in February 2025 from three sub-adult dogs that had died from the disease in Daguping, and one sample was collected in late April 2025 from a sub-adult dog with only mild symptoms in Daguping. All 73 wild giant panda fecal samples tested negative. Detailed information on the positive samples and surrounding strains is provided in Supplementary S5.

**Figure 2:**
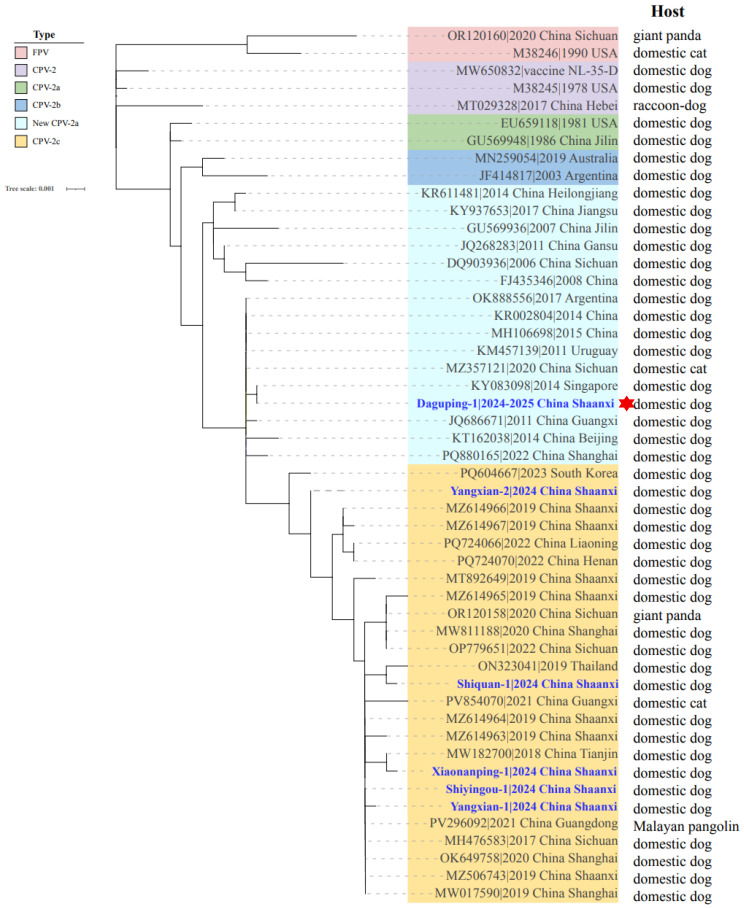
Maximum likelihood tree based on the complete VP2 gene sequences (1755 bp) of CPV. Strains reported in the study area and surrounding regions are highlighted in blue, and the strain isolated in this study is marked with a red asterisk. The wild giant panda-derived strains JX624771 and PP862237 were not included in this phylogenetic analysis because their complete VP2 gene sequences were not available.

The strain sequences of positive samples collected at Daguping prior to the study period (June 2024) and during the study period (February 2025 and April 2025) were identical, identified as New CPV-2a and designated as Daguping-1. All strains isolated from areas surrounding FNNR in the first half of 2024 were CPV-2c. Analysis of key amino acid sites (Table 2) shows that Daguping-1 differs from the New CPV-2a strain JX624771 isolated from a giant panda in Sichuan in 2011 [13] at positions 267 (Y vs. F) and 370 (Q vs. R), but is identical at host-associated key sites (297, 300, 305, 323,324, 426). Phylogenetic analysis indicates that Daguping-1 clusters with a 2014 canine strain from Singapore and is not related to the CPV-2c strains circulating in Shaanxi Province in recent years (Figure 2).

### 3.3. Serological Exposure Risk

At the time of the first sampling, the historical exposure rate among unvaccinated dogs was 63.3%. Among the 194 valid exposure records during the study period (Supplementary S3), the exposure rate was 30.4%. The exposure rate was highest in Daguping (44.1%) and lowest in Sanguanmiao (5.4%); the exposure rate was highest in spring (42.3%) and lowest in autumn (8.6%) (Table 3).

Chi-square tests revealed that location, interval, and age group were significantly associated with exposure (*p* < 0.01). Multivariate regression analysis showed that the risk of exposure was significantly higher for puppies than for adult dogs (OR = 5.37, 95% CI 2.45–12.48); at Daguping, the risk of exposure was significantly higher in Interval 3 (OR = 5.42) and Interval 2 (OR = 4.38) than in Interval 4; the increased risk of exposure at the habitat periphery in Interval 3 and Interval 2 was attenuated (significant interaction term) (Table 4). Vaccination history was not significantly associated with exposure. Complete statistical results are provided in Supplementary S6.

### 3.4. Home Range Data Collection and Overlap Analysis

**Figure 3:**
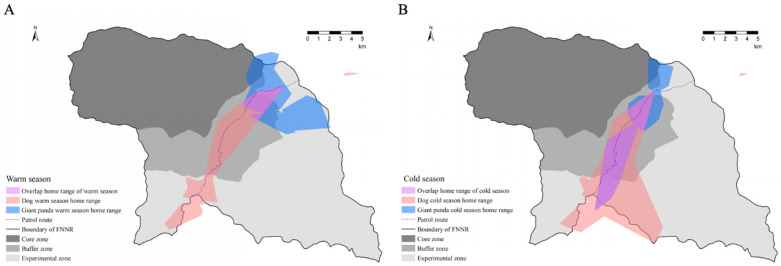
Home ranges calculated using the minimum convex polygon (95% MCP) method. (**A**) Warm season; (**B**) Cold season.

**Figure 4:**
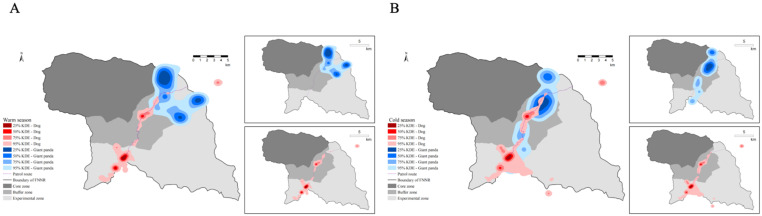
Home ranges calculated using the kernel density estimation (KDE) method. (**A**) Warm season; (**B**) Cold season.

**Figure 5:**
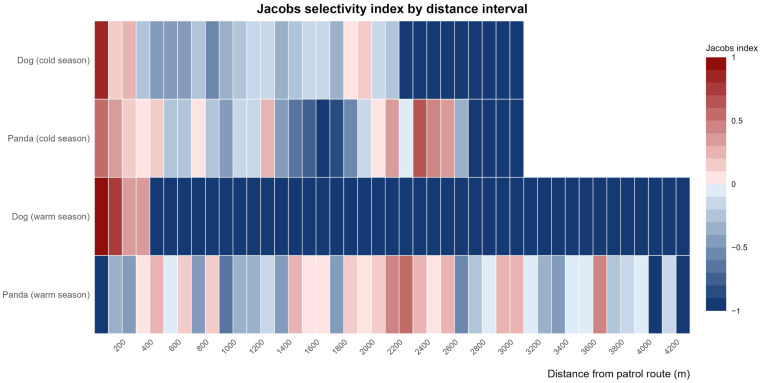
Jacobs selectivity index (D) for giant pandas and domestic dogs relative to the patrol route, shown by distance interval (100 m bins) and season. Positive values (red shades) indicate preference, negative values (blue shades) indicate avoidance. Darker colors represent stronger selectivity (closer to +1 or −1).

**Figure 6:**
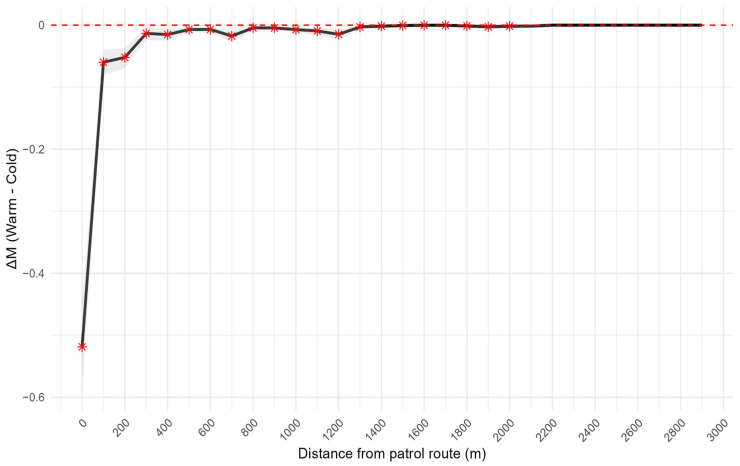
Difference in encounter probability (warm season minus cold season) with 95% bootstrap confidence intervals. Red stars indicate distance bands where the confidence interval does not include 0, suggesting significant seasonal differences.

Using the 95% Minimum Convex Polygon (MCP) method, in the warm season, the home range areas of giant pandas and domestic dogs were 24.789 km^2^ and 24.997 km^2^, respectively, with an overlap area of 4.104 km^2^ and an overlap rate of 8.99%. In the cold season, the home range areas of giant pandas and domestic dogs were 22.381 km^2^ and 45.028 km^2^, respectively, with an overlap area of 14.905 km^2^ and an overlap rate of 28.39% (Figure 3). Using the 95% kernel density estimation (95% KDE) method, in the warm season, the home range areas of giant pandas and domestic dogs were 34.550 km^2^ and 13.415 km^2^, respectively, with an overlap area of 1.220 km^2^ and an overlap rate of 2.61%. In the cold season, the home range areas of giant pandas and domestic dogs were 33.986 km^2^ and 19.760 km^2^, respectively, with an overlap area of 8.645 km^2^ and an overlap rate of 19.17%. The 50% KDE core home range areas of giant pandas and domestic dogs were 7.953 km^2^ and 0.891 km^2^, respectively, in the warm season, and 5.889 km^2^ and 0.946 km^2^, respectively, in the cold season, with no overlap between the two species in either season (Figure 4). The Jacobs index indicated that dogs strongly preferred areas near patrol routes in both seasons; giant pandas preferred the 0–300 m zone during the cold season but avoided it during the warm season (Figure 5). The encounter probability index M was highly concentrated in the 0–100 m zone during the cold season. Bootstrap difference analysis indicated that M values in most zones were significantly lower in the warm season compared to the cold season (Figure 6); see Supplementary S7 for details.

## 4. Discussion

CPV has an extremely broad host range, capable of infecting various carnivorous animals, including giant pandas, as well as non-carnivorous animals such as pangolins [14]. In recent years, although reports of CPV infection in giant pandas have been relatively scarce compared to viruses such as feline panleukopenia virus (FPV) [15,16,17,18], the only confirmed fatal cases of parvovirus infection in wild giant pandas have been caused by CPV. Previous studies have confirmed that domestic dogs may serve as a source of infection or a transmission route in the spillover of CPV to wildlife [19,20,21,22,23].

For ease of discussion, the four sampling intervals are grouped into conventional seasons: winter (Dec–Feb, predominantly Interval 2), spring (Mar–May, predominantly Interval 3), summer (Jun–Aug, encompassing the GPS warm season), and autumn (Sep–Nov, predominantly Interval 1). The GPS cold season (Dec–Mar) spans winter and early spring.

This study found that CPV persists over the long term in free-roaming dog populations in FNNR. During the study period, a New CPV-2a strain (Daguping-1) was isolated from dogs; this strain persisted in the reserve for at least 10 months. Serological studies revealed significant seasonal, geographical, and age-related variations in exposure risk, indicating that the prevalence of CPV in the local canine population is closely associated with cold seasons, high-density gatherings of susceptible individuals, and frequent contact among young dogs. Among all recorded exposures, only a small number were accompanied by clinical disease, viral shedding, or death; the remainder consisted of asymptomatic seroconversions, consistent with the typical pattern of CPV infection in canine populations [24].

Establishing herd immunity within domestic dog populations is a key strategy for reducing the transmission of canine-derived viruses to wildlife [25,26,27,28]. Although the FNNR has conducted annual canine vaccination campaigns in recent years, the actual vaccination coverage remains far below the herd immunity threshold required to block CPV transmission [29], and it has declined seasonally throughout the study period, while the proportion of susceptible dogs has continued to rise. The reasons for this situation are as follows: First, vaccination coverage is unevenly distributed geographically, with relatively high coverage observed only in Daguping and Sanguanmiao. Second, the peak birth season for local dogs occurs in summer and winter, but mass vaccination campaigns are conducted only in summer, resulting in a large number of young dogs in winter and spring being unable to receive timely vaccination after maternal antibodies have waned. Third, due to factors such as an imbalanced sex ratio in the local dog population (with females accounting for only about 30%), population renewal is highly dependent on incoming individuals, resulting in a large influx of non-resident dogs that could not be promptly incorporated into the vaccination program. Additionally, we observed that small-scale dog transport and trade have long existed in villages surrounding the reserve; these transactions may serve as a pathway for the cross-regional transmission of CPV and pose a threat to wildlife [30].

Reported CPV serotypes capable of infecting giant pandas include New CPV-2a and CPV-2c. Analysis of key amino acid sites revealed that Daguping-1 is fully identical to the giant panda-derived strain New CPV-2a JX624771 at host-specific key sites. Daguping-1 is genetically distant from CPV-2c strains circulating in Shaanxi Province in recent years, suggesting that an independently circulating strain may exist within the FNNR dog population, and its original source may not be from the surrounding areas [31]. Although the CPV-2c strains circulating in the surrounding areas have not yet been detected within the reserve, frequent cross-regional movement of dogs indicates that the possibility of future introduction cannot be ruled out. The co-circulation of New CPV-2a and CPV-2c observed in this study, together with the strains isolated during the pre-study period, is broadly consistent with reports from other regions of China, where both variants have been detected in domestic dogs and wildlife.

Two complementary home range estimation methods were used in this study. Although 95% MCP was calculated for each individual and then merged to reduce the inclusion of empty areas, it remains sensitive to outlying locations. For dogs with elongated home ranges, 95% MCP may overestimate the true activity area, whereas for giant pandas, when location points are sparse in certain seasons, 95% MCP may underestimate the home range. In contrast, 95% KDE weights locations by utilization density and is more robust to variation in sample size, better reflecting the differential habitat use of giant pandas. However, because dog activity points are often highly concentrated around dwellings, 95% KDE may underestimate their actual ranging extent. Overall, the two methods characterize home ranges from different perspectives, and their combined use provides a more comprehensive assessment of potential encounter risk.

Spatial analysis identified key ecological conditions for the potential transmission of CPV from domestic dogs to giant pandas. Within the giant pandas’ home ranges, free-roaming domestic dogs move along human patrol routes throughout the year; this movement pattern resembles the tendency of wild canids to follow fixed routes [32]. During winter and spring, giant pandas utilize low-altitude areas more frequently due to resource availability and estrus cycles [33]. At this time, the overlap rate between the total home ranges of giant pandas and dogs is significantly higher than during the warm season, and giant pandas exhibit a preference for the buffer zones near patrol routes. Consequently, the probability index of encounters between the two species is highly concentrated near patrol routes and is significantly higher than during the warm season. The core home ranges of giant pandas and dogs do not overlap in any season, indicating that high-risk zones for potential encounters and disease transmission are more likely to occur in their respective peripheral activity zones or linear corridors.

In early 2019, the local authorities implemented a ban on dog ownership. Compared to studies conducted before the ban, the total number of free-roaming dogs in the area has significantly decreased, and the overlap rate with giant panda home ranges has declined. However, in areas near the core zone, such as Daguping and Sanguanmiao, the total number of dogs still exceeds 50, and some dog packs continue to enter giant pandas’ winter home ranges and key corridors, indicating that the reduction in the total dog population is insufficient to eliminate the risk.

Although this study did not detect the virus in giant panda feces and thus could not directly confirm transmission events, the possibility of low prevalence, transient shedding, or sampling limitations cannot be ruled out. Based on a combination of molecular and spatial evidence, the risk of cross-species CPV infection to local giant pandas remains significant and should not be overlooked.

Overall, the winter and spring months (December through April) present a high-risk window for cross-species transmission of CPV due to the convergence of multiple adverse factors, including increased exposure risk among dog populations, decreased vaccination coverage, a rise in the proportion of susceptible dogs, an expansion of the overlap between dog and giant panda home ranges, and an increased likelihood of encounters. This period is also a critical time for implementing prevention and control measures. It is recommended that the reserve improve its sentinel dog monitoring network and apply the experience gained from CDV control to CPV prevention and control; in addition to the existing summer vaccination program, conduct additional mass vaccination of dog populations during the winter and spring to bridge the immunization gap, thereby raising the annual vaccination coverage rate of local dog populations to over 90%; enforce mandatory tethering of dogs during the winter and spring seasons; strengthen fecal sampling and pathogen monitoring in areas surrounding reserve patrol routes; and install warning signs and surveillance cameras to prohibit dog-related activities in the mountains. At the same time, strictly restrict the introduction of non-native dogs throughout the year by implementing measures such as registration, quarantine, and mandatory vaccination.

Future research should focus on the following four areas: First, conduct serological surveys of giant pandas to systematically assess the historical exposure rate to CPV and the dynamic changes in antibody levels within local wild giant panda populations. Second, evaluate the host adaptability of local circulating strains to giant pandas through methods such as in vitro infection experiments. Third, expand the scope of epidemiological monitoring to include additional species to assess potential transmission networks of CPV among various wildlife. Fourth, investigate the role of the local dog trade in the cross-regional transmission of CPV by conducting continuous viral testing and movement path analysis of traded dogs, thereby providing more empirical evidence to support relevant prevention and control measures.

This study has several limitations. For example, serological testing cannot distinguish between antibodies generated by vaccination and those resulting from natural infection. Since vaccine strains may circulate within dog populations [34], exposure cannot be fully equated with contact with wild-type strains. Additionally, the exposure analysis did not include statistical correction for repeated measurements from the same dog, which may affect the precision of the estimates. Regarding pathogen detection, the relatively short viremia period and the fact that infected dogs may shed virus intermittently may have resulted in low pathogen detection rates in giant panda feces and canine rectal swabs. The small sample size of dogs at study sites such as Daguping led to instability in some statistical estimates. Furthermore, the limited number of individuals tracked via GPS may have caused individual behavioral differences to be averaged out.

## 5. Conclusions

CPV persists long-term in free-roaming dog populations in the FNNR, and current vaccination efforts have failed to establish an effective barrier. Winter and spring (predominantly Intervals 2 & 3) constitute high-risk periods during which dogs face increased exposure risks and have minimal immune protection, while the overlap between dog and panda home ranges and the likelihood of encounters rise significantly. Although no evidence of CPV infection in giant pandas was found, and direct proof of transmission from dogs to pandas cannot be established, canine-derived strains still possess the molecular potential to infect giant pandas. Furthermore, the frequent influx of non-native dogs poses a potential risk of introduction, and the possibility of CPV spillover from domestic dogs to wild giant pandas cannot be ruled out.

## Figures and Tables

**Table 1:** Changes in vaccination coverage and susceptible rate of free-ranging dogs in different areas.

Location	Indicator	Aug. 24	Nov. 24	Feb. 2025	Apr. 2025	Aug. 2025
Daguping	Vaccination coverage	76.70%	59.00%	47.50%	47.20%	43.80%
	Susceptibility rate	7.50%	32.50%	39.10%	41.80%	36.70%
Sanguanmiao	Vaccination coverage	50.00%	50.00%	50.00%	50.00%	40.00%
	Susceptibility rate	25.00%	10.00%	10.00%	0.00%	20.00%
Peripheral habitat	Vaccination coverage	15.40%	15.00%	13.60%	15.80%	16.70%
	Susceptibility rate	0.00%	0.00%	4.50%	5.30%	11.10%
Total	Vaccination coverage	54.20%	46.30%	39.70%	40.70%	36.40%
	Susceptibility rate	8.70%	20.00%	27.00%	28.90%	29.50%

Vaccination coverage = proportion of dogs ≥ 2 months old with vaccination history; Susceptible rate = proportion of unvaccinated dogs aged 2–12 months. Peripheral habitat includes Liangfengya, Yueba, Longtanzi, and Xiaonanping; Total includes all six locations.

**Table 2:** Comparison of key amino acid residues of the VP2 protein.

Strain	Type	Source	Host	Amino Acid Residues at Each Amino Acid Position of the VP2 Protein																
				80	87	93	101	103	232	267	297	300	305	323	324	370	375	426	564	568
X55115	FPV	Australia, 1973	cat	K	M	K	I	V	V	F	S	A	D	D	Y	Q	D	N	N	A
M38245	CPV-2	USA, 1978	dog	R	M	N	I	A	I	F	S	A	D	N	Y	Q	N	N	S	G
MW650832	CPV-2	Vaccine NL-35-D	dog	R	M	N	I	A	I	F	S	A	D	N	Y	Q	E	N	S	G
EU659118	CPV-2a	USA, 1981	dog	R	L	N	T	A	I	F	S	G	Y	N	Y	Q	D	N	S	G
JF414817	CPV-2b	Argentina, 2003	dog	R	L	N	T	A	I	F	N	G	Y	N	Y	Q	D	D	S	G
MH476583	CPV-2c	China, Sichuan, 2017	dog	R	L	N	T	A	I	Y	A	G	Y	N	I	R	D	E	S	G
OR120158	CPV-2c	China, Sichuan, 2020	panda	R	L	N	T	A	I	Y	A	G	Y	N	I	R	D	E	S	G
PP862237	CPV-2c	China, Sichuan, 2020	panda	/	/	/	/	/	I	Y	A	G	Y	N	I	R	D	E	/	/
JQ686671	New CPV-2a	China, Guangxi, 2011	dog	R	L	N	T	A	I	Y	A	G	Y	N	I	Q	D	N	S	G
JX624771	New CPV-2a	China, Sichuan, 2011	panda	/	/	/	/	/	/	F	A	G	Y	N	I	R	D	N	/	/
KX425921	New CPV-2b	India, 2010	dog	R	L	N	T	A	I	Y	A	G	Y	N	Y	Q	D	D	S	G
** Daguping-1	New CPV-2a	China, Shaanxi, 2024–2025	dog	R	L	N	T	A	I	Y	A	G	Y	N	I	Q	D	N	S	G
* Xiaonanping -1	CPV-2c	China, Shaanxi, 2024	dog	R	L	N	T	A	I	Y	A	G	Y	N	I	R	D	E	S	G
* Shiyingou-1	CPV-2c	China, Shaanxi, 2024	dog	R	L	N	T	A	I	Y	A	G	Y	N	I	R	D	E	S	G
* Yangxian-1	CPV-2c	China, Shaanxi, 2024	dog	R	L	N	T	A	I	Y	A	G	Y	N	I	R	D	E	S	G
* Yangxian-2	CPV-2c	China, Shaanxi, 2024	dog	R	L	N	T	A	I	Y	A	G	Y	N	I	R	D	E	S	G
* Shiquan-1	CPV-2c	China, Shaanxi, 2024	dog	R	L	N	T	A	I	Y	A	G	Y	N	I	R	D	E	S	G

Strains marked with ** were isolated during the study period, and those marked with * were isolated in the first half of 2024. Partial VP2 sequences were not available for some strains, so data for certain positions are missing. “/” indicates sequence not available for this position (partial VP2 sequence). “N” = asparagine.

**Table 3:** CPV exposure in domestic dogs by interval and location.

Interval	Location	No. Sampled	No. Exposed	Proportion of Exposed Dogs
Interval 1	Sanguanmiao	7	1	14.3%
	Daguping	19	2	10.5%
	Peripheral habitat	9	0	0
Interval 2	Sanguanmiao	10	1	10%
	Daguping	30	18	60%
	Peripheral habitat	10	0	0
Interval 3	Sanguanmiao	10	0	0
	Daguping	32	21	65.6%
	Peripheral habitat	10	1	10%
Interval 4	Sanguanmiao	10	0	0
	Daguping	37	11	29.7%
	Peripheral habitat	10	4	40%
Total		194	59	30.4%

“Peripheral habitat” includes Liangfengya, Yueba, Longtanzi, and Xiaonanping. Intervals were defined according to sampling periods: Interval 1 (Sep.–Nov. 2024), Interval 2 (Dec. 2024–mid-Feb. 2025), Interval 3 (mid-Feb.–Apr. 2025), Interval 4 (May–Aug. 2025).

**Table 4:** Multivariate Firth logistic regression analysis (significant variables only).

Variable	OR	95% CI	*p*-Value
Age group (juvenile vs. adult)	5.37	2.45–12.48	<0.001
Interval (Interval 3 vs. Interval 4, within Daguping)	5.42	1.87–17.05	0.002
Interval (Interval 2 vs. Interval 4, within Daguping)	4.38	1.50–13.77	0.006
Interval × location (Interval 3 × peripheral habitat)	0.04	0.002–0.38	0.005
Interval × location (Interval 2 × peripheral habitat)	0.01	0–0.22	0.001

Reference categories: adult dogs, Interval 4, and Daguping location. Only significant interaction terms are shown. For full results including non-significant terms, see Supplementary Tables in Supplementary S6.

## Data Availability

The data supporting the findings of this study are available within the article and its Supplementary Materials. The giant panda location data and a portion of the dog GPS tracking data used in this study are not publicly available due to the sensitivity of endangered species location information and the confidentiality requirements of the nature reserve administration. These data may be accessed from the corresponding author upon reasonable request, subject to approval by the relevant authorities.

## Supplementary Materials

The following supporting information can be downloaded at: https://www.mdpi.com/article/10.3390/ani16111686/s1, Supplementary S1: Reference Sequences. Supplementary S2: Antibody Titer Grading and Exposure Criteria (including Table S1. Antibody Titer Grading). Supplementary S3: Summary table of Canine Exposure status (including Table S2. Summary table of Canine Exposure status). Supplementary S4: Summary of Canine Vaccination Coverage and Population Dynamics (including Table S3. Summary of Canine Vaccination Coverage and Table S4. Summary of Canine Population Dynamics). Supplementary S5 Information on Collection of Positive Pathogen Samples (including Table S5. Information on Collection of Positive Pathogen Samples). Supplementary S6: Results of serological statistical analysis (including Table S6.1. Sample size and exposure proportion by location, Table S6.2. Sample size and exposure proportion by Interval, Table S6.3. Sample size and exposure proportion by Interval and location (cross-tabulation), Table S6.4. Chi-square test results for each variable, and Table S6.5. Firth logistic regression results (reference: Interval 4, Daguping)). Supplementary S7: Comparison of Encounter Probability Between Giant Pandas and Domestic Dogs: Cold vs. Warm Seasons (including Table S7.1. Encounter probability index M (95% CI) for cold and warm seasons, and the difference ΔM (95% CI) by distance band; Table S7.2. Jacobs index and related metrics for cold season; Table S7.3. Jacobs index and related metrics for warm season; Figure S7.1. Comparison of encounter probability between cold and warm seasons. Shaded areas represent 95% bootstrap confidence intervals. Supplementary S8: Sample and individual counts for canine and giant panda samples across five time points.
