# Supplementary material for: Surveillance of Parvovirus in Free-Roaming Dogs in the Qinling Mountains and Assessment of the Risk of Cross-Species Transmission to Giant Pandas
Source: Animals (Basel). 2026 May 31;16(11):1686. doi: 10.3390/ani16111686 (PMC13255924; doi:10.3390/ani16111686)
Supplement: Supplementary file 1 [file animals-16-01686-s001.zip › Supplementary 1 Reference Sequences.pdf]

Supplementary 1 : Reference Sequences

>M38246|FPV|1990 USA|cat

ATGAGTGATGGAGCAGTTCAACCAGACGGTGGTCAACCTGCTGTCAGAAA
TGAAAGAGCTACAGGATCTGGGAACGGGTCTGGAGGCGGGGGTGGTGGTG
GTTCTGGGGGTGTGGGGATTTCTACGGGTACTTTCAATAATCAGACGGAATT
TAAATTTTTGGAAAACGGGTGGGTGGAAATCACAGCAAACTCAAGCAGAC
TTGTACATTTAAATATGCCAGAAAGTGAAAATTATAAAAGAGTAGTTGTAAA
TAATATGGATAAAACTGCAGTTAAAGGAAACATGGCTTTAGATGATATTCAT
GTACAAATTGTAACACCTTGGTCATTGGTTGATGCAAATGCTTGGGGAGTTT
GGTTTAATCCAGGAGATTGGCAACTAATTGTTAATACTATGAGTGAGTTGCA
TTTAGTTAGTTTTGAACAAGAAATTTTTAATGTTGTTTTAAAGACTGTTTCA
GAATCTGCTACTCAGCCACCAACTAAAGTTTATAATAATGATTTAACTGCAT
CATTGATGGTTGCATTAGATAGTAATAATACTATGCCATTTACTCCAGCAGCT
ATGAGATCTGAGACATTGGGTTTTTATCCATGGAAACCAACCATACCAACTC
CATGGAGATATTATTTTCAATGGGATAGAACATTAATACCATCTCATACTGGA
ACTAGTGGCACACCAACAAATGTATATCATGGTACAGATCCAGATGATGTTC
AATTTTATACTATTGAAAATTCTGTGCCAGTACACTTACTAAGAACAGGTGA
TGAATTTGCTACAGGAACATTTTTTTTTGATTGCAAACCATGTAGACTAACA
CATACATGGCAAACAAATAGAGCATTGGGCTTACCACCATTTTTAAATTCTT
TGCCTCAATCTGAAGGAGCTACTAACTTTGGTGATATAGGAGTTCAACAAG
ATAAAAGACGTGGTGTAACTCAAATGGGAAATACAGACTATATTACTGAAG
CTACTATTATGAGACCAGCTGAGGTTGGTTATAGTGCACCATATTATTCTTTT
GAAGCGTCTACACAAGGGCCATTTAAAACACCTATTGCAGCAGGACGGGG
GGGAGCGCAAACAGATGAAAATCAAGCAGCAGATGGTGATCCAAGATATG
CATTTGGTAGACAACATGGTCAAAAAACTACTACAACAGGAGAAACACCT
GAGAGATTTACATATATAGCACATCAAGATACAGGAAGATATCCAGAAGGA
GATTGGATTCAAAATATTAACTTTAACCTTCCTGTAACAAATGATAATGTATT
GCTACCAACAGATCCAATTGGAGGTAAAACAGGAATTAACTATACTAATATA
TTTAATACTTATGGTCCTTTAACTGCATTAAATAATGTACCACCAGTTTATCC
AAATGGTCAAATTTGGGATAAAGAATTTGATACTGACTTAAAACCAAGACT
TCATGTAAATGCACCATTTGTTTGTCAAAATAATTGTCCTGGTCAATTATTTG
TAAAAGTTGCGCCTAATTTAACGAATGAATATGATCCTGATGCATCTGCTAAT
ATGTCAAGAATTGTAACTTATTCAGATTTTTGGTGGAAAGGTAAATTAGTAT
TTAAAGCTAAACTAAGAGCATCTCATACTTGGAATCCAATTCAACAAATGA
GTATTAATGTAGATAACCAATTTAACTATGTACCAAATAATATTGGAGCTATG
AAAATTGTATATGAAAAATCTCAACTAGCACCTAGAAAATTATATTAA

>OR120160|FPV|2020 China Sichuan|panda

ATGAGTGATGGAGCAGTTCAACCAGACGGTGGTCAACCTGCTGTCAGAAA
TGAAAGAGCTACAGGATCTGGGAACGGGTCTGGAGGCGGGGGTGGTGGTG
GTTCTGGGGGTGTGGGGATTTCTACGGGTACTTTCAATAATCAGACGGAATT
TAAATTTTTGGAAAACGGATGGGTGGAAATCACAGCAAACTCAAGCAGAC
TTGTACATTTAAATATGCCAGAAAGTGAAAATTATAAAAGAGTAGTTGTAAA

TAATATGGATAAAACTGCAGTTAAAGGAAACATGGCTTTAGATGATACTCAT
GTACAAMTTGTAACACCTTGGTCATTGGTTGATGCAAATGCTTGGGGAGTT
TGGTTTAATCCAGGAGATTGGCAACTAATTGTTAATACTATGAGTGAGTTGC
ATTTAGTTAGTTTTGAACAAGAAATTTTTAATGTTGTTTTAAAGACTGTTTC
AGAATCTGCTACTCAACCACCAACTAAAGTTTATAATAATGATTTAACTGCA
TCATTGATGGTTGCATTAGATAGTAATAATACTATGCCATTTACTCCAGCAGC
TATGAGATCTGAGACATTGGGTTTTTATCCATGGAAACCAACCATACCAACT
CCATGGAGATATTATTTTCAATGGGATAGAACATTAATACCATCTCATACTGG
AACTAGTGGCACACCAACAAATGTATATTATGGTACAGATCCAGATGATGTT
CAATTTTATACTATTGAAAATTCTGTGCCAGTACACTTACTAAGAACAGGTG
ATGAATTTGCTACAGGAACATTTTTTTTGATTGTAAACCATGTAGACTAAC
ACATACATGGCAAACAAATAGAGCATTGGGCTTACCACCATTTCTAAATTCT
TTGCCTCAATCTGAAGGAGCTACTAACTTTGGTGATATAGGAGTTCAACAA
GATAAAAGACGTGGTGTAACACAAATGGGAAATACAGACTATATTACTGAA
GCTACTATTATGAGACCAGCTGAGGTTGGTTATAGTGCACCATATTATTCTTT
TGAAGCATCTACACAAGGACCATTTAAAACACCTATTGCAGCAGGACGGGG
GGGAGCGCAAACAGATGAAAACCAAGCAGCAGATGGTGATCCAAGATATG
CATTTGGTAGACAACATGGTCAAAAAACTACTACAACAGGAGAAACACCC
GAGAGATTTACATATATAGCACATCAAGATACAGGAAGATATCCAGAAGGA
GATTGGATTCAAAATATCAACTTTAACCTTCCTGTAACAAATGATAATGTATT
GCTACCAACAGATCCAATTGGAGGTAAAACAGGAATTAACTATACTAATATA
TTTAATACTTATGGTCCTTTAACTGCATTAAATAATGTACCACCAGTTTATCC
AAATGGTCAAATTTGGGATAAAGAATTTGATACTGACTTAAAACCAAGACT
TCATGTAAATGCACCATTTGTTTGTCAAAATAATTGTCCTGGTCAATTATTTG
TAAAAGTTGCGCCTAATTTAACAAATGAATATGATCCTGATGCATCTGCTAAT
ATGTCAAGAATTGTAACTTACTCAGATTTTTGGTGGAAAGGTAAATTAGTTT
TTAAAGCTAAACTAAGAGCATCTCATACTTGGAATCCAATTCAACAAATGA
GTATTAATGTAGATAACCAATTTAACTATGTACCAAATAATATTGGAGCTATG
AAAATTGTATATGAAAAATCTCAACTAGCACCTAGAAAATTATATTAA

>M38245|CPV-2|1978 USA|dog

ATGAGTGATGGAGCAGTTCAACCAGACGGTGGTCAACCTGCTGTCAGAAA
TGAAAGAGCTACAGGATCTGGGAACGGGTCTGGAGGCGGGGGTGGTGGTG
GTTCTGGGGGTGTGGGGATTTCTACGGGTACTTTCAATAATCAGACGGAATT
TAAATTTTTGGAAAACGGATGGGTGGAAATCACAGCAAACTCAAGCAGAC
TTGTACATTTAAATATGCCAGAAAGTGAAAATTATAGAAGAGTGGTTGTAAA
TAATATGGATAAAACTGCAGTTAACGGAAACATGGCTTTAGATGATATTCAT
GCACAAATTGTAACACCTTGGTCATTGGTTGATGCAAATGCTTGGGGAGTT
TGGTTTAATCCAGGAGATTGGCAACTAATTGTTAATACTATGAGTGAGTTGC
ATTTAGTTAGTTTTGAACAAGAAATTTTTAATGTTGTTTTAAAGACTGTTTC
AGAATCTGCTACTCAGCCACCAACTAAAGTTTATAATAATGATTTAACTGCA
TCATTGATGGTTGCATTAGATAGTAATAATACTATGCCATTTACTCCAGCAGC
TATGAGATCTGAGACATTGGGTTTTTATCCATGGAAACCAACCATACCAACT
CCATGGAGATATTATTTTCAATGGGATAGAACATTAATACCATCTCATACTGG

AACTAGTGGCACACCAACAAATATATACCATGGTACAGATCCAGATGATGTT
CAATTTTATACTATTGAAAATTCTGTGCCAGTACACTTACTAAGAACAGGTG
ATGAATTTGCTACAGGAACATTTTTTTTTGATTGTAAACCATGTAGACTAAC
ACATACATGGCAAACAAATAGAGCATTGGGCTTACCACCATTTCTAAATTCT
TTGCCTCAATCTGAAGGAGCTACTAACTTTGGTGATATAGGAGTTCAACAA
GATAAAAGACGTGGTGTAACTCAAATGGGAAATACAAACTATATTACTGAA
GCTACTATTATGAGACCAGCTGAGGTTGGTTATAGTGCACCATATTATTCTTT
TGAGGCGTCTACACAAGGGCCATTTAAAACACCTATTGCAGCAGGACGGG
GGGGAGCGCAAACAGATGAAAATCAAGCAGCAGATGGTAATCCAAGATAT
GCATTTGGTAGACAACATGGTCAAAAAACTACCACAACAGGAGAAACACC
TGAGAGATTTACATATATAGCACATCAAGATACAGGAAGATATCCAGAAGGA
GATTGGATTCAAAATATTAACTTTAACCTTCCTGTAACAAATGATAATGTATT
GCTACCAACAGATCCAATTGGAGGTAAAACAGGAATTAACTATACTAATATA
TTTAATACTTATGGTCCTTTAACTGCATTAAATAATGTACCACCAGTTTATCC
AAATGGTCAAATTTGGGATAAAGAATTTGATACTGACTTAAAACCAAGACT
TCATGTAAATGCACCATTTGTTTGTCAAAATAATTGTCCTGGTCAATTATTTG
TAAAAGTTGCGCCTAATTTAACAAATGAATATGATCCTGATGCATCTGCTAAT
ATGTCAAGAATTGTAACTTACTCAGATTTTTGGTGGAAAGGTAAATTAGTAT
TTAAAGCTAAACTAAGAGCCTCTCATACTTGGAATCCAATTCAACAAATGA
GTATTAATGTAGATAACCAATTTAACTATGTACCAAGTAATATTGGAGGTATG
AAAATTGTATATGAAAAATCTCAACTAGCACCTAGAAAATTATATTAA

>MT029328|CPV-2|2017 China Hebei|raccoon-dog

ATGAGTGATGGAGCAGTTCAACCAGACGGTGGTCAGCCTGCTGTCAGAAA
TGAAAGAGCTACAGGATCTGGGAACGGGACTGGAGGCGGGGGTGGTGGT
GGTTCTGGTGGTGTGGGGATTTCTACGGGTACTTTCAATAATCAGACGGAAT
TTAAATTTTTGGAAAACGGATGGGTGGAAATCACAGCAAACTCAAGCAGA
CTTGTACATTTAAATATGCCAGAAAGTGAAAATTATAGAAGAGTGGTTGTAA
ATAATATGGATAAAACTGCAGTTAATGGAAACATGGCTTTAGATGATATTCAT
GCACAAATTGTAACACCTTGGTCATTGGTTGATGCAAATGCTTGGGGAGTT
TGGTTTAATCCAGGAGATTGGCAACTAATTGTTAATACTATGAGTGAATTGC
ATTTAGTTAGTTTTGAACAAGAAATTTTTAATGTTGTTTTAAAGACTGTTTC
AGAATCTGCTACTCAGCCACCAACTAAAGTTTATAATAATGATTTAACTGCA
TCATTGATGGTTGCATTAGATAGTAATAATACTATGCCATTTACTCCAGCAGC
TATGAGATCTGAGACATTGGGTTTTTATCCATGGAAACCAACCATACCAACT
CCATGGAGATATTATTTCCAATGGGATAGAACATTAATACCATCTCATACTGG
AACTAGTGGCACACCAACAAATATATACCATGGTACAGATCCAGATGATGTT
CAATTTTATACTATTGAAAATTCTGTGCCAGTACACTTACTAAGAACAGGTG
ATGAATTTGCTACAGGAACATTTTTTTTTGATTGTAAACCATGTAGACTAAC
ACATACATGGCAAACAAATAGAGCATTGGGCTTACCACCATTTCTAAATTCT
TTGCCTCAAGCTGAAGGAGCTACTAACTTTGGTGATATAGGAGTTCAACAA
GATAAAAGACGTGGTGTAACTCAAATGGGAAATACAAACTATATTACTGAA
GCTACTATTATGAGACCAGCTGAGGTTGGTTATAGTGCACCATATTATTCTTT
TGAGGCGTCTACACAAGGGCCATTTAAAACACCTATTGCAGCAGGACGGG

GGGGAGCGCAAACAGATGAAAATCAAGCAGCAGATGGTGATCCAAGATAT
GCATTTGGTAGACAACATGGTCAAAAAACTACCACAACAGGAGAAACACC
TGAGAGATTTACATATATAGCACATCAAGATACAGGAAGATATCCAGAAGGA
GATTGGATTCAAAATACTAACTTTAACCTTCCTGTAACAAATGATAATGTATT
GCTACCAACAGATCCAATTGGAGGTAAAACAGGAATTAACTATACTAATATA
TTTAATACTTATGGTCCTTTAACTGCATTAAATAATGTACCACCAGTTTATCC
AAATGGTCAAATTTGGGATAAAGAATTTGATACTGACTTAAAACCAAGACT
TCATGTAAATGCACCATTTGTTTGTCAAAATAATTGTCCTGGTCAATTATTTG
TAAAAGTTGCGCCTAATTTAACAAATGAATATGATCCTGATGCATCTGCTAAT
ATGTCAAGAATTGTAACTTACTCAGATTTTTGGTGGAAAGGTAAATTAGTAT
TTAAAGCTAAACTAAGAGCCTCTCATACTTGGAATCCAATTCAACAAATGA
GTATTAATGTAGATAACCAATTTAACTATCTACCAAGTAATATTGGAGGTATG
AAAATTGTATATGAAAAATCTCAACTAGCACCTAGAAAATTATATTAA

>MW650832|CPV-2|vaccine NL-35-D|dog

ATGAGTGATGGAGCAGTTCAACCAGACGGTGGTCAACCTGCTGTCAGAAA
TGAAAGAGCTACAGGATCTGGGAACGGGTCTGGAGGCGGGGGTGGTGGTG
GTTCTGGGGGTGTGGGGATTTCTACGGGTGCTTTCAATAATCAGACGGAAT
TTAAATTTTTGGAAAACGGATGGGTGGAAATCACAGCAAACTCAAGCAGA
CTTGTACATTTAAATATGCCAGAAAGTGAAAATTATAGAAGAGTGGTTGTAA
ATAATATGGATAAAACTGCAGTTAACGGAAACATGGCTTTAGATGATATTCA
TGCACAAATTGTAACACCTTGGTCATTGGTTGATGCAAATGCTTGGGGAGT
TTGGTTTAATCCAGGAGATTGGCAACTAATTGTTAATACTATGAGTGAGTTG
CATTTAGTTAGTTTTGAACAAGAAATTTTTAATGTTGTTTTAAAGACTGTTT
CAGAATCTGCTACTCAGCCACCAACTAAAGTTTATAATAATGATTTAACTGC
ATCATTGATGGTTGCATTAGATAGTAATAATACTATGCCATTTACTCCAGCAG
CTATGAGATCTGAGACATTGGGTTTTTATCCATGGAAACCAACCATACCAAC
TCCATGGAGATATTATTTTCAATGGGATAGAACATTAATACCATCTCATACTG
GAACTAGTGGCACACCAACAAATATATACCATGGTACAGATCCAGATGATGT
TCAATTTTATACTATTGAAAATTCTGTGCCAGTACACTTACTAAGAACAGGT
GATGAATTTGCTACAGGAACATTTTTTTTTGATTGTAAACCATGTAGACTAA
CACATACATGGCAAACAAATAGAGCATTGGGCTTACCACCATTTCTAAATTC
TTTGCCTCAATCTGAAGGAGCTACTAACTTTGGTGATATAGGAGTTCAACA
AGATAAAAGACGTGGTGTAACTCAAATGGGAAATACAAACTATATTACTGA
AGCTACTATTATGAGACCAGCTGAGGTTGGTTATAGTGCACCATATTATTCTT
TTGAGGCGTCTACACAAGGGCCATTTAAAACACCTATTGCAGCAGGACGGG
GGGGAGCGCAAACAGATGAAAATCAAGCAGCAGATGGTGAACCAAGATAT
GCATTTGGTAGACAACATGGTCAAAAAACTACCACAACAGGAGAAACACC
TGAGAGATTTACATATATAGCACATCAAGATACAGGAAGATATCCAGAAGGA
GATTGGATTCAAAATATTAACTTTAACCTTCCTGTAACGAATGATAATGTATT
GCTACCAACAGATCCAATTGGAGGTAAAACAGGAATTAACTATACTAATATA
TTTAATACTTATGGTCCTTTAACTGCATTAAATAATGTACCACCAGTTTATCC
AAATGGTCAAATTTGGGATAAAGAATTTGATACTGACTTAAAACCAAGACT
TCATGTAAATGCACCATTTGTTTGTCAAAATAATTGTCCTGGTCAATTATTTG

TAAAAGTTGCGCCTAATTTAACAAATGAATATGATCCTGATGCATCTGCTAAT
ATGTCAAGAATTGTAACTTACTCAGATTTTTGGTGGAAAGGTAAATTAGTAT
TTAAAGCTAAACTAAGAGCCTCTCATACTTGGAATCCAATTCAACAAATGA
GTATTAATGTAGATAACCAATTTAACTATGTACCAAGTAATATTGGAGGTATG
AAAATTGTATTTGAAAAATCTCAACTAGCACCTAGAAAATTATATTAA

>GU569948|CPV-2a|1986 China Jilin|dog

ATGAGTGATGGAGCAGTTCAACCAGACGGTGGTCAACCTGCTGTCAGAAA
TGAAAGAGCTACAGGATCTGGGAACGGGTCTGGAGGCGGGGGTGGTGGTG
GTTCTGGGGGTGTGGGGATTTCTACGGGTACTTTCAATAATCAGACGGAATT
TAAATTTTTGGAAAACGGATGGGTGGAAATCACAGCAAACTCAAGCAGAC
TTGTACATTTAAATATGCCAGAAAGTGAAAATTATAGAAGAGTGGTTGTAAA
TAATTTGGATAAAACTGCAGTTAACGGAAACATGGCTTTAGATGATACCCAT
GCACAAATTGTAACACCTTGGTCATTGGTTGATGCAAATGCTTGGGGAGTT
TGGTTTAATCCAGGAGATTGGCAACTAATTGTTAATACTATGAGTGAGTTGC
ATTTAGTTAGTTTTGAACAAGAAATTTTTAATGTTGTTTTAAAGACTGTTTC
AGAATCTGCTACTCAGCCACCAACTAAAGTTTATAATAATGATTTAACTGCA
TCATTGATGGTTGCATTAGATAGTAATAATACTATGCCATTTACTCCAGCAGC
TATGAGATCTGAGACATTGGGTTTTTATCCATGGAAACCAACCATACCAACT
CCATGGAGATATTATTTTCAATGGGATAGAACATTAATACCATCTCATACTGG
AACTAGTGGCACACCAACAAATATATACCATGGTACAGATCCAGATGATGTT
CAATTTTATACTATTGAAAATTCTGTGCCAGTACACTTACTAAGAACAGGTG
ATGAATTTGCTACAGGAACATTTTTTTTTGATTGTAAACCATGTAGACTAAC
ACATACATGGCAAACAAATAGAGCATTGGGCTTACCACCATTTCTAAATTCT
TTGCCTCAATCTGAAGGAGGTACTAACTTTGGTTATATAGGAGTTCAACAAG
ATAAAAGACGTGGTGTAACTCAAATGGGAAATACAAACTATATTACTGAAG
CTACTATTATGAGACCAGCTGAGGTTGGTTATAGTGCACCATATTATTCTTTT
GAGGCGTCTACACAAGGGCCATTTAAAACACCTATTGCAGCAGGACGGGG
GGGAGCGCAAACAGATGAAAATCAAGCAGCAGATGGTAATCCAAGATATG
CATTTGGTAGACAACATGGTCAAAAAACTACCACAACAGGAGAAACACCT
GAGAGATTTACATATATAGCACATCAAGATACAGGAAGATATCCAGAAGGA
GATTGGATTCAAAATATTAACTTTAACCTTCCTGTAACAAATGATAATGTATT
GCTACCAACAGATCCAATTGGAGGTAAAACAGGAATTAACTATACTAATATA
TTTAATACTTATGGTCCTTTAACTGCATTAAATAATGTACCACCAGTTTATCC
AAATGGTCAAATTTGGGATAAAGAATTTGATACTGACTTAAAACCAAGACT
TCATGTAAATGCACCATTTGTTTGTCAAAATAATTGTCCTGGTCAATTATTTG
TAAAAGTTGCGCCTAATTTAACAAATGAATATGATCCTGATGCATCTGCTAAT
ATGTCAAGAATTGTAACTTACTCAGATTTTTGGTGGAAAGGTAAATTAGTAT
TTAAAGCTAAACTAAGAGCCTCTCATACTTGGAATCCAATTCAACAAATGA
GTATTAATGTAGATAACCAATTTAACTATGTACCAAGTAATATTGGAGGTATG
AAAATTGTATATGAAAAATCTCAACTAGCACCTAGAAAATTATATTAA

>EU659118|CPV-2a|1981 USA|dog

ATGAGTGATGGAGCAGTTCAACCAGACGGTGGTCAACCTGCTGTCAGAAA

TGAAAGAGCTACAGGATCTGGGAACGGGTCTGGAGGCGGGGGTGGTGGTG
GTTCTGGGGGTGTGGGGATTTCTACGGGTACTTTCAATAATCAGACGGAATT
TAAATTTTTGGAAAACGGATGGGTGGAAATCACAGCAAACTCAAGCAGAC
TTGTACATTTAAATATGCCAGAAAGTGAAAATTATAGAAGAGTGGTTGTAAA
TAATTTGGATAAAACTGCAGTTAACGGAAACATGGCTTTAGATGATACCCAT
GCACAAATTGTAACACCTTGGTCATTGGTTGATGCAAATGCTTGGGGAGTT
TGGTTTAATCCAGGAGATTGGCAACTAATTGTTAATACTATGAGTGAGTTGC
ATTTAGTTAGTTTTGAACAAGAAATTTTTAATGTTGTTTTAAAGACTGTTTC
AGAATCTGCTACTCAGCCACCAACTAAAGTTTATAATAATGATCTAACTGCA
TCATTGATGGTTGCATTAGATAGTAATAATACTATGCCATTTACTCCAGCAGC
TATGAGATCTGAGACATTGGGCTTTTATCCATGGAAACCAACCATACCAACT
CCATGGAGATATTATTTTCAATGGGATAGAACATTAATACCATCTCATACTGG
AACTAGTGGCACACCAACAAATATATACCATGGTACAGATCCAGATGATGTT
CAATTTTATACTATTGAAAATTCTGTGCCAGTACACTTACTAAGAACAGGTG
ATGAATTTGCTACAGGAACATTTTTTTTTGATTGTAAACCATGTAGACTAAC
ACATACATGGCAAACAAATAGAGCATTGGGCTTACCACCATTTCTAAATTCT
TTGCCTCAATCTGAAGGAGGTACTAACTTTGGTTATATAGGAGTTCAACAAG
ATAAAAGACGTGGTGTAACTCAAATGGGAAATACAAACTATATTACTGAAG
CTACTATTATGAGACCAGCTGAGGTTGGTTATAGTGCACCATATTATTCTTTT
GAGGCGTCTACACAAGGGCCATTTAAAACACCTATTGCAGCAGGACGGGG
GGGAGCGCAAACAGATGAAAATCAAGCAGCAGATGGTGATCCAAGATATG
CATTTGGTAGACAACATGGTCAAAAAACTACCACAACAGGAGAAACACCT
GAGAGATTTACATATATAGCACATCAAGATACAGGAAGATATCCAGAAGGA
GATTGGATTCAAAATATTAACTTTAACCTTCCTGTAACAAATGATAATGTATT
GCTACCAACAGATCCAATTGGAGGTAAAACAGGAATTAACTATACTAATATA
TTTAATACTTATGGTCCTTTAACTGCATTAAATAATGTACCACCAGTTTATCC
AAATGGTCAAATTTGGGATAAAGAATTTGATACTGACTTAAAACCAAGACT
TCATGTAAATGCACCATTTGTTTGTCAAAATAATTGTCCTGGTCAATTATTTG
TAAAAGTTGCGCCTAATTTAACAAATGAATATGATCCTGATGCATCTGCTAAT
ATGTCAAGAATTGTAACTTACTCAGATTTTTGGTGGAAAGGTAAATTAGTAT
TTAAAGCTAAACTAAGAGCCTCTCATACTTGGAATCCAATTCAACAAATGA
GTATTAATGTAGATAACCAATTTAACTATGTACCAAGTAATATTGGAGGTATG
AAAATTGTATATGAAAAATCTCAACTAGCACCTAGAAAATTATATTAA

>OK888556|New CPV-2a|2017 Argentina|dog

ATGAGTGATGGAGCAGTTCAACCAGACGGTGGTCAGCCTGCTGTCAGAAA
TGAAAGAGCTACAGGATCTGGGAACGGGTCTGGAGGCGGGGGTGGTGGTG
GTTCTGGGGGTGTGGGGATTTCTACGGGTACTTTCAATAATCAGACGGAATT
TAAATTTTTGGAAAACGGATGGGTGGAAATCACAGCAAACTCAAGCAGAC
TTGTACATTTAAATATGCCAGAAAGTGAAAATTATAGAAGAGTGGTTGTAAA
TAATTTGGATAAAACTGCAGTTAACGGAAACATGGCTTTAGATGATACCCAT
GCACAAATTGTAACACCTTGGTCATTGGTTGATGCAAATGCTTGGGGAGTT
TGGTTTAATCCAGGAGATTGGCAACTAATTGTTAATACTATGAGTGAGTTGC
ATTTAGTTAGTTTTGAACAAGAAATTTTTAATGTTGTTTTAAAGACTGTTTC

AGAATCTGCTACTCAGCCACCAACTAAAGTTTATAATAATGATTTAACTGCA
TCATTGATGGTTGCATTAGATAGTAATAATACTATGCCATTTACTCCAGCAGC
TATGAGATCTGAGACATTGGGTTTTTATCCATGGAAACCAACCATACCAACT
CCATGGAGATATTATTTTCAATGGGATAGAACATTAATACCATCTCATACTGG
AACTAGTGGCACACCAACAAATATATACCATGGTACAGATCCAGATGATGTT
CAATTTTACACTATTGAAAATTCTGTGCCAGTACACTTACTAAGAACAGGTG
ATGAATTTGCTACAGGAACATTTTATTTTGATTGTAAACCATGTAGACTAAC
ACACACATGGCAAACAAATAGAGCATTGGGCTTACCACCATTTCTAAATTCT
TTGCCTCAAGCTGAAGGAGGTACTAACTTTGGTTATATAGGAGTTCAACAA
GATAAAAGACGTGGTGTAACTCAAATGGGAAATACAAACATTATTACTGAA
GCTACTATTATGAGACCAGCTGAGGTTGGTTATAGTGCACCATATTATTCTTT
TGAGGCGTCTACACAAGGGCCATTTAAAACACCTATTGCAGCAGGACGGG
GGGGAGCGCAAACAGATGAAAATCAAGCAGCAGATGGTGATCCAAGATAT
GCATTTGGTAGACAACATGGTCAAAAAACTACCACAACAGGAGAAACACC
TGAGAGATTTACATATATAGCACATCAAGATACAGGAAGATATCCAGAAGGA
GATTGGATTCAAAATATTAACTTTAACCTTCCTGTAACAAATGATAATGTATT
GCTACCAACAGATCCAATTGGAGGTAAAGCAGGAATTAACTATACTAATATA
TTTAATACTTATGGTCCTTTAACTGCATTAAATAATGTACCACCAGTTTATCC
AAATGGTCAAATTTGGGATAAAGAATTTGATACTGACTTAAAACCAAGACT
TCATGTAAATGCACCATTTGTTTGTCAAAATAATTGTCCTGGTCAATTATTTG
TAAAAGTTGCGCCTAATTTAACAAATGAATATGATCCTGATGCATCTGCTAAT
ATGTCAAGAATTGTAACTTACTCAGATTTTTGGTGGAAAGGTAAATTAGTAT
TTAAAGCTAAACTAAGAGCCTCTCATACTTGGAATCCAATTCAACAAATGA
GTATTAATGTAGATAACCAATTTAACTATGTACCAAGTAATATTGGAGGTATG
AAAATTGTATATGAAAAATCTCAACTAGCACCTAGAAAATTATACTAA

>JQ268283|New CPV-2a|2011 China Gansu|dog

ATGAGTGATGGAGCAGTTCAACCAGACGGTGGTCAGCCTGCTGTCAGAAA
TGAAAGAGCTACAGGATCTGGGAACGGGTCTGGAGGCGGGGGTGGTGGTG
GTTCTGGGGGTGTGGGGATTTCTACGGGTACTTTCAATAATCAGACGGAATT
TAAATTTTTGGAAAACGGATGGGTGGAAATCACAGCAAACTCAAGCAGAC
TTGTACATTTAAATATGCCAGAAAGTGAAAATTATAGAAGAGTGGTTGTAAA
TAATTTGGATAAAACTGCAGTTAACGGAAACATGGCTTTAGATGATACCCAT
GCACAAATTGTAACACCTTGGTCATTGGTTGATGCAAATGCTTGGGGAGTT
TGGTTTAATCCAGGAGATTGGCAACTAATTGTTAATACTATGAGTGAGTTGC
ATTTAGTTAGTTTTGAACAAGAAATTTTTAATGTTGTTTTAAAGACTGTTTC
AGAATCTGCTACTCAGCCACCAACTAAAGTTTATAATAATGATTTAACTGCA
TCATTGATGGTTGCATTAGATAGTAATAATACTATGCCATTTACTCCAGCAGC
TATGAGATCTGAGACATTGGGTTTTTATCCATGGAAACCAACCATACCAACT
CCATGGAGATATTATTTTCAATGGGATAGAACATTAATACCATCTCATACTGG
AACTAGTGGCACACCAACAAATATATACCATGGTACAGATCCAGATGATGTT
CAATTTTATACTATTGAAAATTCTGTGCCAGTACACTTACTAAGAACAGGTG
ATGAATTTGCTACAGGAACATTTTTTTTTGATTGTAAACCATGCAGACTAAC
ACATACATGGCAAACAAATAGAGCATTGGGCTTACCACCATTTCTAAATTCT

TTGCCTCAAGCTGAAGGAGGTACTAACTTTGGTTATATAGGAGTTCAACAA
GATAAAAGACGTGGTGTAACTCAAATGGGAAATACAAACATTATTACTGAA
GCTACTATTATGAGACCAGCTGAGGTTGGTTATAGTGCACCATATTATTCTTT
TGAGGCGTCTACACAAGGGCCATTTAAAACACCTATTGCAGCAGGACGGG
GGGGAGCGCAAACAGATGAAAATCAAGCAGCAGATGGTGATCCAAGATAT
GCATTTGGTAGACAACATGGTCAAAAAACTACCACAACAGGAGAAACACC
TGAGAGATTTACATATATAGCACATCAAGATACAGGAAGATATCCAGAAGGA
GATTGGATTCAAAATATTAACTTTAACCTTCCTGTAACAAATGATAATGTATT
GCTACCAACAGATCCAATTGGAGGTAAAACAGGAATTAACTATACTAATATA
TTTAATACTTATGGTCCTTTAACTGCATTAAATAATGTACCACCAGTTTATCC
AAATGGTCAAATTTGGGATAAAGAATTTGATACTGACTTAAAACCAAGACT
TCATGTAAATGCACCATTTGTTTGTCAAAATAATTGTCCCGGTCAATTATTTG
TAAAAGTTGCGCCTAATTTAACAAATGAATATGATCCTGATGCATCTGCTAAT
ATGTCAAGAATTGTAACTTACTCAGATTTTTGGTGGAAAGGTAAATTAGTAT
TTAAAGCTAAACTAAGAGCCTCTCATACTTGGAATCCAATTCAACAAATGA
GTATTAATGTAGATAACCAATTTAACTATGTACCAAGTAATATTGGAGGTATG
AAAATTGTATATGAGAAATCTCAACTAGCACCTAGAAAATTATATTAA

>KY937653|New CPV-2a|2017 China Jiangsu|dog

ATGAGTGATGGAGCAGTTCAACCAGACGGTGGTCAGCCTGCTGTCAGAAA
TGAAAGAGCTACAGGATCTGGGAACGGGTCTGGAGGCGGGGGTGGTGGTG
GTTCTGGGGGTGTGGGGATTTCTACGGGTACTTTCAATAATCAGACGGAATT
TAAATTTTTGGAAAACGGATGGGTGGAAATCACAGCAAACTCAAGCAGAC
TTGTACATTTAAATATGCCAGAAAGTGAAAATTATAGAAGAGTGGTTGTAAA
TAATTTGGATAAAACTGCAGTTAACGGAAACATGGCTTTAGATGATACCCAT
GCACAAATTGTAACACCTTGGTCATTGGTTGATGCAAATGCTTGGGGAGTT
TGGTTTAATCCAGGAGATTGGCAACTAATTGTTAATACTATGAGTGAGTTGC
ATTTAGTTAGTTTTGAACAAGAAATTTTTAATGTTGTTTTAAAGACTGTTTC
AGAATCTGCTACTCAGCCACCAACTAAAGTTTATAATAATGATTTAACTGCA
TCATTGATGGTTGCATTAGATAGTAATAATACTATGCCATTTACTCCAGCAGC
TATGAGATCTGAGACATTGGGTTTTTATCCATGGAAACCAACCATACCAACT
CCATGGAGATATTATTTTCAATGGGATAGAACATTAATACCATCTCATACTGG
AACTAGTGGCACACCAACAAATATATACCATGGTACAGATCCAGATGATGTT
CAATTTTATACTATTGAAAATTCTGTGCCAGTACACTTACTAAGAACAGGTG
ATGAATTTGCTACAGGAACATTTTTTTTTGATTGTAAACCATGCAGACTAAC
ACATACATGGCAAACAAATAGAGCATTGGGCTTACCACCATTTCTAAATTCT
TTGCCTCAAGCTGAAGGAGGTACTAACTTTGGTTATATAGGAGTTCAAGAA
GATAAAAGACGTGGTGTAACTCAAATGGGAAATACAAACATTATTACTGAA
GCTACTATTATGAGACCAGCTGAGGTTGGTTATAGTGCACCATATTATTCTTT
TGAGGCGTCTACACAAGGGCCATTTAAAACACCTATTGCAGCAGGACGGG
GGGGAGCGCAAACAGATGAAAATCGAGCAGCAGATGGTGATCCAAGATAT
GCATTTGGTAGACAACATGGTCAAAAAACTACCACAACAGGAGAAACACC
TGAGAGATTTACATATATAGCACATCAAGATACAGGAAGATATCCAGAAGGA
GATTGGATTCAAAATATTAACTTTAACCTTCCTGTAACAAATGATAATGTATT

GCTACCAACAGATCCAATTGGAGGTAAAACAGGAATTAACTATACTAATATA
TTTAATACTTATGGTCCTTTAACTGCATTAAATAATGTACCACCAGTTTATCC
AAATGGTCAAATTTGGGATAAAGAATTTGATACTGACTTAAAACCAAGACT
TCATGTAAATGCACCATTTGTTTGTCAAAATAATTGTCCTGGTCAATTATTTG
TAAAAGTTGCGCCTAATTTAACAAATGAATATGATCCTGATGCATCTGCTAAT
ATGTCAAGAATTGTAACTTACTCAGATTTTTGGTGGAAAGGTAAATTAGTAT
TTAAAGCTAAACTAAGAGCCTCTCATACTTGGAATCCAATTCAACAAATGA
GTATTAATGTAGATAACCAATTTAACTATGTACCAAGTAATATTGGAGGTATG
AAAATTGTATATGAAAAATCTCAACTAGCACCTAGAAAATTATACTAA

>KR611481|New CPV-2a|2014 China Heilongjiang|dog

ATGAGTGATGGAGCAGTTCAACCAGACGGTGGTCAGCCTGCTGTCAGAAA
TGAAAGAGCTACAGGATCTGGGAACGGGTCTGGAGGCGGGGGTGGTGGTG
GTTCTGGGGGTGTGGGGATTTCTACGGGTACTTTCAATAATCAGACGGAATT
TAAATTTTTGGAAAACGGATGGGTGGAAATCACAGCAAACTCAAGCAGAC
TTGTACATTTAAATATGCCAGAAAGTGAAAATTATAGAAGAGTGGTTGTAAA
TAATTTGGATAAAACTGCAGTTAACGGAAACATGGCTTTAGATGATACCCAT
GCACAAATTGTAACACCTTGGTCATTGGTTGATGCAAATGCTTGGGGAGTT
TGGTTTAATCCAGGAGATTGGCAACTAATTGTTAATACTATGAGTGAGTTGC
ATTTAGTTAGTTTTGAACAAGAAATTTTTAATGTTGTTTTAAAGACTGTTTC
AGAATCTGCTACTCAGCCACCAACTAAAATTTATAATAATGATTTAACTGCA
TCATTGATGGTTGCATTAGATAGTAATAATACTATGCCATTTACTCCAGCAGC
TATGAGATCTGAGACATTGGGTTTTTATCCATGGAAACCAACCATACCAACT
CCATGGAGATATTATTTTCAATGGGATAGAACATTAATACCATCTCATACTGG
AACTAGTGGCACACCAACAAATATATACCATGGTACAGATCCAGATGATGTT
CAATTTTATACTATTGAAAATTCTGTGCCAGTACACTTACTAAGAACAGGTG
ATGAATTTGCTACAGGAACATTTTTTTTTGATTGTAAACCATGCAGACTAAC
ACATACATGGCAAACAAATAGAGCATTGGGCTTACCACCATTTCTAAATTCT
TTGCCTCAAGCTGAAGGAGGTACTAACTTTGGTTATATAGGAGTTCAAGAA
GATAAAAGACGTGGTGTAACTCAAATGGGAAATACAAACATTATTACTGAA
GCTACTATTATGAGACCAGCTGAGGTTGGTTATAGTGCACCATATTATTCTTT
TGAGGCGTCTACACAAGGGCCATTTAAAACACCTATTGCAGCAGGACGGG
GGGGAGCGCAAACAGATGAAAATCGAGCAGCAGATGGTGATCCAAGATAT
GCATTTGGTAGACAACATGGTCAAAAAACTACCACAACAGGAGAAACACC
TGAGAGATTTACATATATAGCACATCAAGATACAGGAAGATATCCAGAAGGA
GATTGGATTCAAAATATTAACTTTAACCTTCCTGTAACAAATGATAATGTATT
GCTACCAACAGATCCAATTGGAGGTAAAACAGGAATTAACTATACTAATATA
TTTAATACTTATGGTCCTTTAACTGCATTAAATAATGTACCACCAGTTTATCC
AAATGGTCAAATTTGGGATAAAGAATTTGATACTGACTTAAAACCAAGACT
TCATGTAAATGCACCATTTGTTTGTCAAAATAATTGTCCTGGTCAATTATTTG
TAAAAGTTGCGCCTAATTTAACAAATGAATATGATCCTGATGCATCTGCTAAT
ATGTCAAGAATTGTAACTTACTCAGATTTTTGGTGGAAAGGTAAATTAGTAT
TTAAAGCTAAACTAAGAGCCTCTCATACTTGGAATCCAATTCAACAAATGA
GTATTAATGTAGATAACCAATTTAACTATGTACCAAGTAATATTGGAGGTATG

AAAATTGTATATGAAAAATCTCAACTAGCACCTAGAAAATTATATTAA

>KM457139|New CPV-2a|2011 Uruguay|dog

ATGAGTGATGGAGCAGTTCAACCAGACGGTGGTCAGCCTGCTGTCAGAAA
TGAAAGAGCTACAGGATCTGGGAACGGGTCTGGAGGCGGGGGTGGTGGTG
GTTCTGGGGGTGTGGGGATTTCTACGGGTACTTTCAATAATCAGACGGAATT
TAAATTTTTGGAAAACGGATGGGTGGAAATCACAGCAAACTCAAGCAGAC
TTGTACATTTAAATATGCCAGAAAGTGAAAATTATAGAAGAGTGGTTGTAAA
TAATTTGGATAAAACTGCAGTTAACGGAAACATGGCTTTAGATGATACCCAT
GCACAAATTGTAACACCTTGGTCATTGGTTGATGCAAATGCTTGGGGAGTT
TGGTTTAATCCAGGAGATTGGCAACTAATTGTTAATACTATGAGTGAGTTGC
ATTTAGTTAGTTTTGAACAAGAAATTTTTAATGTTGTTTTAAAGACTGTTTC
AGAATCTGCTACTCAGCCACCAACTAAAGTTTATAATAATGATTTAACTGCA
TCATTGATGGTTGCATTAGATAGTAATAATACTATGCCATTTACTCCAGCAGC
TATGAGATCTGAGACATTGGGTTTTTATCCATGGAAACCAACCATACCAACT
CCATGGAGATATTATTTTCAATGGGATAGAACATTAATACCATCTCATACTGG
AACTAGTGGCACACCAACAAATATATACCATGGTACAGATCCAGATGATGTT
CAATTTTACACTATTGAAAATTCTGTGCCAGTACACTTACTAAGAACAGGTG
ATGAATTTGCTACAGGAACATTTTATTTTGATTGTAAACCATGTAGACTAAC
ACACACATGGCAAACAAATAGAGCATTGGGCTTACCACCATTTCTAAATTCT
TTGCCTCAAGCTGAAGGAGGTACTAACTTTGGTTATATAGGAGTTCAACAA
GATAAAAGACGTGGTGTAACTCAAATGGGAAATACAAACATTATTACTGAA
GCTACTATTATGAGACCAGCTGAGGTTGGTTATAGTGCACCATATTATTCTTT
TGAGGCGTCTACACAAGGGCCATTTAAAACACCTATTGCAGCAGGACGGG
GGGGAGCGCAAACAGATGAAAATCAAGCAGCAGATGGTGATCCAAGATAT
GCATTTGGTAGACAACATGGTCAAAAAACTACCACAACAGGAGAAACACC
TGAGAGATTTACATATATAGCACATCAAGATACAGGAAGATATCCAGAAGGA
GATTGGATTCAAAATATTAACTTTAACCTTCCTGTAACAAATGATAATGTATT
GCTACCAACAGATCCAATTGGAGGTAAAGCAGGAATTAACTATACTAATATA
TTTAATACTTATGGTCCTTTAACTGCATTAAATAATGTACCACCAGTTTATCC
AAATGGTCAAATTTGGGATAAAGAATTTGATACTGACTTAAAACCAAGACT
TCATGTAAATGCACCATTTGTTTGTCAAAATAATTGTCCTGGTCAATTATTTG
TAAAAGTTGCGCCTAATTTAACAAATGAATATGATCCTGATGCATCTGCTAAT
ATGTCAAGAATTGTAACTTACTCAGATTTTTGGTGGAAAGGTAAATTAGTAT
TTAAAGCTAAACTAAGAGCCTCTCATACTTGGAATCCAATTCAACAAATGA
GTATTAATGTAGATAACCAATTTAACTATGTACCAAGTAATATTGGAGGTATG
AAAATTGTATATGAAAAATCTCAACTAGCACCTAGAAAATTATACTAA

>KY083098|New CPV-2a|2014 Singapore|dog

ATGAGTGATGGAGCAGTTCAACCAGACGGTGGTCAGCCTGCTGTCAGAAA
TGAAAGAGCTACAGGATCTGGGAACGGGTCTGGAGGCGGGGGTGGTGGTG
GTTCTGGGGGTGTGGGGATTTCTACGGGTACTTTCAATAATCAGACGGAATT
TAAATTTTTGGAAAACGGATGGGTGGAAATCACAGCAAACTCAAGCAGAC
TTGTACATTTAAATATGCCAGAAAGTGAAAATTATAGAAGAGTGGTTGTAAA

TAATTTGGATAAAACTGCAGTTAACGGAAACATGGCTTTAGATGATACCCAT
GCACAAATTGTAACACCTTGGTCATTGGTTGATGCAAATGCTTGGGGAGTT
TGGTTTAATCCAGGAGATTGGCAACTAATTGTTAATACTATGAGTGAGTTGC
ATTTAGTTAGTTTTGAACAAGAAATTTTTAATGTTGTTTTAAAGACTGTTTC
AGAATCTGCTACTCAGCCACCAACTAAAGTTTATAATAATGATTTAACTGCA
TCATTGATGGTTGCATTAGACAGTAATAATACTATGCCATTTACTCCAGCAGC
TATGAGATCTGAGACATTGGGTTTTTATCCATGGAAACCAACCATACCAACT
CCATGGAGATATTATTTTCAATGGGATAGAACATTAATACCATCTCATACTGG
AACTAGTGGCACACCAACAAATATATACCATGGTACAGATCCAGATGATGTT
CAATTTTACACTATTGAAAATTCTGTGCCAGTACACTTACTAAGAACAGGTG
ATGAATTTGCTACAGGAACATTTTATTTTGATTGTAAACCATGTAGACTAAC
ACACACATGGCAAACAAATAGAGCATTGGGCTTACCACCATTTCTAAATTCT
TTGCCTCAAGCTGAAGGAGGTACTAACTTTGGTTATATAGGAGTTCAACAA
GATAAAAGACGTGGTGTAACTCAAATGGGAAATACAAACATTATTACTGAA
GCTACTATTATGAGACCAGCTGAGGTTGGTTATAGTGCACCATATTATTCTTT
TGAGGCGTCTACACAAGGGCCATTTAAAACACCTATTGCAGCAGGACGGG
GGGGAGCGCAAACAGATGAAAATCAAGCAGCAGATGGTGATCCAAGATAT
GCATTTGGTAGACAACATGGTCAAAAAACTACCACAACAGGAGAAACACC
TGAGAGATTTACATATATAGCACATCAAGATACAGGAAGATATCCAGAAGGA
GATTGGATTCAAAATATTAACTTTAACCTTCCTGTAACAAATGATAATGTATT
GCTACCAACAGATCCAATTGGAGGTAAAGCAGGAATTAACTATACTAATATA
TTTAATACTTATGGTCCTTTAACTGCATTAAATAATGTACCACCAGTTTATCC
AAATGGTCAAATTTGGGATAAAGAATTTGATACTGACTTAAAACCAAGACT
TCATGTAAATGCACCATTTGTTTGTCAAAATAATTGTCCTGGTCAATTATTTG
TAAAAGTTGCGCCTAATTTAACAAATGAATATGATCCTGATGCATCTGCTAAT
ATGTCAAGAATTGTAACTTACTCAGATTTTTGGTGGAAAGGTAAATTAGTAT
TTAAAGCTAAACTAAGAGCCTCTCATACTTGGAATCCAATTCAACAAATGA
GTATTAATGTAGATAACCAATTTAACTATGTACCAAGTAATATTGGAGGTATG
AAAATTGTATATGAAAAATCTCAACTAGCACCTAGAAAATTATACTAA

>KR002804|New CPV-2a|2014 China|dog

ATGAGTGATGGAGCAGTTCAACCAGACGGTGGTCAGCCTGCTGTCAGAAA
TGAAAGAGCTACAGGATCTGGGAACGGGTCTGGAGGCGGGGGTGGTGGTG
GTTCTGGGGGTGTGGGGATTTCTACGGGTACTTTCAATAATCAGACGGAATT
TAAATTTTTGGAAAACGGATGGGTGGAAATCACAGCAAACTCAAGCAGAC
TTGTACATTTAAATATGCCAGAAAGTGAAAATTATAGAAGAGTGGTTGTAAA
TAATTTGGATAAAACTGCAGTTAACGGAAACATGGCTTTAGATGATACCCAT
GCACAAATTGTAACACCTTGGTCATTGGTTGATGCAAATGCTTGGGGAGTT
TGGTTTAATCCAGGAGATTGGCAACTAATTGTTAATACTATGAGTGAGTTGC
ATTTAGTTAGTTTTGAACAAGAAATTTTTAATGTTGTTTTAAAGACTGTTTC
AGAATCTGCTACTCAGCCACCAACTAAAGTTTATAATAATGATTTAACTGCA
TCATTGATGGTTGCATTAGATAGTAATAATACTATGCCATTTACTCCAGCAGC
TATGAGATCTGAGACATTGGGTTTTTATCCATGGAAACCAACCATACCAACT
CCATGGAGATATTATTTTCAATGGGATAGAACATTAATACCATCTCATACTGG

AACTAGTGGCACACCAACAAATATATACCATGGTACAGATCCAGATGATGTT
CAATTTTACACTATTGAAAATTCTGTGCCAGTACACTTACTAAGAACAGGTG
ATGAATTTGCTACAGGAACATTTTATTTTGATTGTAAACCATGTAGACTAAC
ACACACATGGCAAACAAATAGAGCATTGGGCTTACCACCATTTCTAAATTCT
TTGCCTCAAGCTGAAGGAGGTACTAACTTTGGTTATATAGGAGTTCAACAA
GATAAAAGACGTGGTGTAACTCAAATGGGAAATACAAACATTATTACTGAA
GCTACTATTATGAGACCAGCTGAGGTTGGTTATAGTGCACCATATTATTCTTT
TGAGGCGTCTACACAAGGGCCATTTAAAACACCTATTGCAGCAGGACGGG
GGGGAGCGCAAACAGATGAAAATCAAGCAGCAGATGGTGATCCAAGATAT
GCATTTGGTAGACAACATGGTCAAAAAACTACCACAACAGGAGAAACACC
TGAGAGATTTACATATATAGCACATCAAGATACAGGAAGATATCCAGAAGGA
GATTGGATTCAAAATATTAACTTTAACCTTCCTGTAACAAATGATAATGTATT
GCTACCAACAGATCCAATTGGAGGTAAAGCAGGAATTAACTATACTAATATA
TTTAATACTTATGGTCCTTTAACTGCATTAAATAATGTACCACCAGTTTATCC
AAATGGTCAAATTTGGGATAAAGAATTTGATACTGACTTAAAACCAAGACT
TCATGTAAATGCACCATTTGTTTGTCAAAATAATTGTCCTGGTCAATTATTTG
TAAAAGTTGCGCCTAATTTAACAAATGAATATGATCCTGATGCATCTGCTAAT
ATGTCAAGAATTGTAACTTACTCAGATTTTTGGTGGAAAGGTAAATTAGTAT
TTAAAGCTAAACTAAGAGCCTCTCATACTTGGAATCCAATTCAACAAATGA
GTATTAATGTAGATAACCAATTTAACTATGTACCAAGTAATATTGGAGGTATG
AAAATTGTATATGAAAAATCTCAACTAGCACCTAGAAAATTATACTAA

>JQ686671|New CPV-2a|2011 China Guangxi|dog

ATGAGTGATGGAGCAGTTCAACCAGACGGTGGTCAGCCTGCTGTCAGAAA
TGAAAGAGCTACAGGATCTGGGAACGGGTCTGGAGGCGGGGGTGGTGGTG
GTTCTGGGGGTGTGGGGATTTCTACGGGTACTTTCAATAATCAGACGGAATT
TAAATTTTTGGAAAACGGATGGGTGGAAATCACAGCAAACTCAAGCAGAC
TTGTACATTTAAATATGCCAGAAAGTGAAAATTATAGAAGAGTGGTTGTAAA
TAATTTGGATAAAACTGCAGTTAACGGAAACATGGCTTTAGATGATACCCAT
GCACAAATTGTAACACCTTGGTCATTGGTTGATGCAAATGCTTGGGGAGTT
TGGTTTAATCCAGGAGATTGGCAACTAATTGTTAATACTATGAGTGAGTTGC
ATTTAGTTAGTTTTGAACAAGAAATTTTTAATGTTGTTTTAAAGACTGTTTC
AGAATCTGCTACTCAGCCACCAACTAAAGTTTATAATAATGATTTAACTGCA
TCATTGATGGTTGCATTAGATAGTAATAATACTATGCCATTTACTCCAGCAGC
TATGAGATCTGAGACATTGGGTTTTTATCCATGGAAACCAACCATACCAACT
CCATGGAGATATTATTTTCAATGGGATAGAACATTAATACCATCTCATACTGG
AACTAGTGGCACACCAACAAATATATACCATGGTACAGATCCAGATGATGTT
CAATTTTACACTATTGAAAATTCTGTGCCAGTACACTTACTAAGAACAGGTG
ATGAATTTGCTACAGGAACATTTTATTTTGATTGTAAACCATGTAGACTAAC
ACACACATGGCAAACAAATAGAGCATTGGGCTTACCACCATTTCTAAATTCT
TTGCCTCAAGCTGAAGGAGGTACTAACTTTGGTTATATAGGAGTTCAACAA
GATAAAAGACGTGGTGTAACTCAAATGGGAAATACAAACATTATTACTGAA
GCTACTATTATGAGACCAGCTGAGGTTGGTTATAGTGCACCATATTATTCTTT
TGAGGCGTCTACACAAGGGCCATTTAAAACACCTATTGCAGCAGGACGGG

GGGGAGCGCAAACAGATGAAAATCAAGCAGCAGATGGTGATCCAAGATAT
GCATTTGGTAGACAACATGGACAAAAAACTACCACAACAGGAGAAACACC
TGAGAGATTTACATATATAGCACATCAAGATACAGGAAGATATCCAGAAGGA
GATTGGATTCAAAATATTAACTTTAACCTTCCTGTAACAAATGATAATGTATT
GCTACCAACAGATCCAATTGGAGGTAAAGCAGGAATTAACTATACTAATATA
TTTAATACTTATGGTCCTTTAACTGCATTAAATAATGTACCACCAGTTTATCC
AAATGGTCAAATTTGGGATAAAGAATTTGATACTGACTTAAAACCAAGACT
TCATGTAAATGCACCATTTGTTTGTCAAAATAATTGTCCTGGTCAATTATTTG
TAAAAGTTGCGCCTAATTTAACAAATGAATATGATCCTGATGCATCTGCTAAT
ATGTCAAGAATTGTAACTTACTCAGATTTTTGGTGGAAAGGTAAATTAGTAT
TTAAAGCTAAACTAAGAGCCTCTCATACTTGGAATCCAATTCAACAAATGA
GTATTAATGTAGATAACCAATTTAACTATGTACCAAGTAATATTGGAGGTATG
AAAATTGTATATGAAAAATCTCAACTAGCACCTAGAAAATTATACTAA

>PQ880165|New CPV-2a|2022 China Shanghai|dog

ATGAGTGATGGAGCAGTTCAACCAGACGGTGGTCAGCCTGCTGTCAGAAA
TGAAAGAACTACAGGATCTGGGAACGGGTCTGGAGGCGGGGGTGGTGGTG
GTTCTGGGGGTGTGGGGATTTCTACGGGTACTTTCAATAATCAGACGGAATT
TAAATTTTTGGAAAACGGATGGGTGGAAATCACAGCAAACTCAAGCAGAC
TTGTACATTTAAATATGCCAGAAAGTGAAAATTATAGAAGAGTGGTTGTAAA
TAATTTGGATAAAACTGCAGTTAACGGAAACATGGCTTTAGATGATACCCAT
GCACAAATTGTAACACCTTGGTCATTGGTTGATGCAAATGCTTGGGGAGTT
TGGTTTAATCCAGGAGATTGGCAACTAATTGTTAATACTATGAGTGAGTTGC
ATTTAGTTAGTTTTGAACAAGAAATTTTTAATGTTGTTTTAAAGACTGTTTC
AGAATCTGCTACTCAGCCACCAACTAAAGTTTATAATAATGATTTAACTGCA
TCATTGATGGTTGCATTAGATAGTAATAATACTATGCCATTTACTCCAGCAGC
TATGAGATCTGAGACATTGGGTTTTTATCCATGGAAACCAACCATACCAACT
CCATGGAGATATTATTTTCAATGGGATAGAACATTAATACCATCTCATACTGG
AACTAGTGGCACACCAACAAATATATACCATGGTACAGATCCAGATGATGTT
CAATTTTACACTATTGAAAATTCTGTGCCAGTACACTTACTAAGAACAGGTG
ATGAATTTGCTACAGGAACATTTTATTTTGATTGTAAACCATGTAGACTAAC
ACACACATGGCAAACAAATAGAGCATTGGGCTTACCACCATTTCTAAATTCT
TTGCCTCAAGCTGAAGGAGGTACTAACTTTGGTTATATAGGAGTTCAACAA
GATAAAAGACGTGGTGTGACTCAAATGGGAAATACAAACATTATTACTGAA
GCTACTATTATGAGACCAGCTGAGGTTGGTTATAGTGCACCATATTATTCTTT
TGAGGCGTCTACACAAGGGCCATTTAAAACACCTATTGCAGCAGGACGGG
GGGGAGCGCAAACAGATGAAAATCAAGCAGCAGATGGTGATCCAAGATAT
GCATTTGGTAGACAACATGGTCAAAAAACTACCACAACAGGAGAAACACC
TGAGAGATTTACATATATAGCACATCAAGATACAGGAAGATATCCAGAAGGA
GATTGGATTCAAAATATTAACTTTAACCTTCCTGTAACAAATGATAATGTATT
GCTACCAACAGATCCAATTGGAGGTAAAGCAGGAATTAACTATACTAATATA
TTTAATACTTATGGTCCTTTAACTGCATTAAATAATGTACCACCAGTTTATCC
AAATGGTCAAATTTGGGATAAAGAATTTGATACTGACTTAAAACCAAGACT
TCATGTAAATGCACCATTTGTTTGTCAAAATAATTGTCCTGGTCAATTATTTG

TAAAAGTTGCGCCTAATTTAACAAATGAATATGATCCTGATGCATCTGCTAAT
ATGTCAAGAATTGTAACTTACTCAGATTTTTGGTGGAAAGGTAAATTAGTAT
TTAAAGCTAAACTAAGAGCCTCTCATACTTGGAATCCAATTCAACAAATGA
GTATTAATGTAGATAACCAATTTAACTATGTACCAAGTAATATTGGAGGTATG
AAAATTGTATATGAAAAATCTCAACTAGCACCTAGAAAATTATATTAA

>GU569936|New CPV-2a|2007 China Jilin|dog

ATGAGTGATGGAGCAGTTCAACCAGACGGTGGTCCGCCTGCTGTCAGAAA
TGAAAGAGCTACAGGATCTGGGAACGGGTCTGGAGGCGGGGGTGGTGGTG
GTTCTGGGGGTGTGGGGATTTCTACGGGTACTTTCAATAATCAGACGGAATT
TAAATTTTTGGAAAACGGATGGGTGGAAATCACAGCAAACTCAAGCAGAC
TTGTACATTTAAATATGCCAGAAAGTGAAAATTATAGAAGAGTGGTTGTAAA
TAATTTGGATAAAACTGCAGTTAACGGAAACATGGCTTTAGATGATACCCAT
GCACAAATTGTAACACCTTGGTCATTGGTTGATGCAAATGCTTGGGGAGTT
TGGTTTAATCCAGGAGATTGGCAACTAATTGTTAATACTATGAGTGAGTTGC
ACTTAGTTAGTTTTGAACAAGAAATTTTTAATGTTGTTTTAAAGACTGTTTC
AGAATCTGCTACTCAGCCACCAACTAAAGTTTATAATAATGATTTAACTGCA
TCATTGATGGTTGCATTAGATAGTAATAATACTATGCCATTTACTCCAGCAGC
TATGAGATCTGAGACATTGGGTTTTTATCCATGGAAACCAACCATACCAACT
CCATGGAGATATTATTTTCAATGGGATAGAACATTAATACCATCTCATACTGG
AACTAGTGGCACACCAACAAATATATACCATGGTACAGATCCAGATGATGTT
CAATTTTATACTATTGAAAATTCTGTGCCAGTACACTTACTAAGAACAGGTG
ATGAATTTGCTACAGGAACATTTTTTTTTGATTGTAAACCATGCAGACTAAC
ACATACATGGCAAACAAATAGAGCATTGGGCTTACCACCATTTCTAAATTCT
TTGCCTCAAGCTGAAGGAGGTACTAACTTTGGTTATATAGGAGTTCAACAA
GATAAAAGGCGTGGTGTAACTCAAATGGGAAATACAAACATTATTACTGAA
GCTACTATTATGAGACCAGCTGAGGTTGGTTATAGTGCACCATATTATTCTTT
TGAGGCGTCTACACAAGGGCCATTTAAAACACCTATTGCAGCAGGACGGG
GGGGAGCGCAAACAGATGAAAATCAAGCAGCAGATGGTGATCCAAGATAT
GCATTTGGTAGACAACATGGTCAGAAAACTACCACAACAGGAGAAACACC
TGAGAGATTTACATATATAGCACATCAAGATACAGGAAGGTATCCAGAAGG
AGATTGGATTCAAAATATTAACTTTAACCTTCCTGTAACAAATGATAATGTAT
TGCTACCAACAGATCCAATTGGAGGTAAAACAGGAATTAACTATACTAATAT
ATTTAATACTTATGGTCCTTTAACTGCATTAAATAATGTACCACCAGTTTATC
CAAATGGTCAAATTTGGGATAAAGAATTTGATACTGACTTAAAACCAAGAC
TTCATGTAAATGCACCATTTGTTTGTCAAAATAATTGTCCTGGTCAATTATTT
GTAAAAGTTGCGCCTAATTTAACAAATGAATATGATCCTGATGCATCTGCTA
ATATGTCAAGAATTGTAACTTACTCAGATTTTTGGTGGAAAGGTAAATTAGT
ATTTAAAGCTAAACTAAGAGCCTCTCATACTTGGAGTCCAATTCAACAAAT
GAGTATTAATGTAGATAACCAATTTAACTATGTACCAAGTAATATTGGAGCTA
TGAAAATTGTATATGAAAAATCTCAACTAGCACCTAGAAAATTATATTAA

>FJ435346|New CPV-2a|2008 China|dog

ATGGGTGATGGAGCAGTTCAACCAGACGGTGGTCAGCCTGCTGTCAGAAA

TGAAAGAGCTACAGGATCTGGGAACGGGTCTGGAGGCGGGGGTGGTGGTG
GTTCTGGGGGTGTGGGGATTTCTACGGGTACTTTCAATAATCAGACGGAATT
TAAATTTTTGGAAAACGGATGGGTGGAAATCACAGCAAACTCAAGCAGAC
TTGTACATTTAAATATGCCAGAAAGTGAAAATTATAGAAGAGTGGTTGTAAA
TAATTTGGATAAAACTGCAGTTAACGGAAACATGGCTTTAGATGATACCCAT
GCACAAATTGTAACACCTTGGTCATTGGTTGATGCAAATGCTTGGGGAGTT
TGGTTTAATCCAGGAGATTGGCAACTAATTGTTAATACTATGAGTGAGTTGC
ATTTAGTTAGTTTTGAACAAGAAATTTTTAATGTTGTTTTAAAGACTGTTTC
AGAATCTGCTACTCAGCCACCAACTAAAGTTTATAATAATGATTTAACTGCA
TCATTGATGGTTGCATTAGATAGTAATAATACTATGCCATTTACTCCAGCAGC
TATGAGATCTGAGACATTGGGTTTTTATCCATGGAAACCAACCATACCAACT
CCATGGAGATATTATTTTCAATGGGATAGAACATTAATACCATCTCATACTGG
AACTAGTGGCACACCAACAAATATATACCATGGTACAGATCCAGATGATGTT
CAATTTTATACTATTGAAAATTCTGTGCCAGTACACTTACTAAGAACAGGTG
ATGAATTTGCTACAGGAACATTTTTTTTTGATTGTAAACCATGTAGACTAAC
ACATACATGGCAAACAAATAGAGCATTGGGCTTACCACCATTTCTAAATTCT
TTGCCTCAAGCTGAAGGAGGTACTAACTTTGGTTATATAGGAGTTCAACAA
GATAAAAGACGTGGTGTAACTCAAATGGGAAATACAAACATTATTACTGAA
GCTACTATTATGAGACCAGCTGAGGTTGGTTATAGTGCACCATATTATTCTTT
TGAAGCGTCTACACAAGGGCCATTTAAAACACCTATTGCAGCAGGACGGG
GGGGAGCGCAAACAGATGAAAATCAAGCAGCAGATGGTGATCCAAGATAT
GCATTTGGTAGACAACATGGTCAGAAAACTACCACAACAGGAGAAACACC
TGAGAGATTTACATATATAGCACATCAAGATACAGGAAGATATCCAGAAGGA
GATTGGATTCAAAATATTAACTTTAACCTTCCTGTAACAAATGATAATGTATT
GCTACCAACAGATCCAATTGGAGGTAAAACAGGAATTAACTATACTAATATA
TTTAATACTTATGGTCCTTTAACTGCATTAAATAATGTACCACCAGTTTATCC
AAATGGTCAAATTTGGGATAAAGAATTTGATACTGACTTAAAACCAAGACT
TCATGTAAATGCACCATTTGTTTGTCAAAATAATTGTCCCGGTCAATTATTTG
TAAAAGTTGCGCCTAATTTAACAAATGAATATGATCCTGATGCATCTGCTAAT
ATGTCAAGAATTGTAACTTACTCAGATTTTTGGTGGAAAGGTAAATTAGTAT
TTAAAGCTAAACTAAGAGCCTCTCATACTTGGAATCCAATTCAACAAATGA
GTATTAATGTAGATAACCAATTTAACTATGTACCAAGTAACATTGGAGGTATG
AAAATTGTATATGAGAAATCTCAACTAGCACCTAGAAAATTATATTAA

>KT162038|New CPV-2a|2014 China Beijing|dog

ATGAGTGATGGAGCAGTTCAACCAGACGGTGGTCAGCCTGCTGTCAGAAA
TGAAAGAGCTACAGGATCTGGGAACGGGTCTGGAGGCGGGGGTGGTGGTG
GTTCTGGGGGTGTGGGGATTTCTACGGGTACTTTCAATAATCAGACGGAATT
TAAATTTTTGGAAAACGGATGGGTGGAAATCACAGCAAACTCAAGCAGAC
TTGTACATTTAAATATGCCAGAAAGTGAAAATTATAGAAGAGTGGTTGTAAA
TAATTTGGATAAAACTGCAGTTAACGGAAACATGGCTTTAGATGACACCCA
TGCACAAATTGTAACACCTTGGTCATTGGTTGATGCAAATGCTTGGGGAGT
TTGGTTTAATCCAGGAGATTGGCAACTAATTGTTAATACTATGAGTGAGTTG
CATTTAGTTAGTTTTGAACAAGAAATTTTTAATGTTGTTTTAAAGACTGTTT

CAGAATCTGCTACTCAGCCACCAACTAAAGTTTATAATAATGATTTAACTGC
ATCATTGATGGTTGCATTAGATAGTAATAATACTATGCCATTTACTCCAGCAG
CTATGAGATCTGAGACATTGGGTTTTTATCCATGGAAACCAACCATACCAAC
TCCATGGAGATATTATTTTCAATGGGATAGAACATTAATACCATCTCATACTG
GAACTAGTGGCACACCAACAAATATATACCATGGTACAGATCCAGATGATGT
TCAATTTTACACTATTGAAAATTCTGTGCCAGTACACTTACTAAGAACAGGT
GATGAATTTGCTACAGGAACATTTTATTTTGATTGTAAACCATGTAGACTAA
CACACACATGGCAAAAAAAAAGAGCATTGGGCTTACCACCATTTCTAAATT
CTTTGCCTCAAGCTGAAGGAGGTACTAACTTTGGTTATATAGGAGTTCAAC
AAGATAAAAGACGTGGTGTAACTCAAATGGGAAATACAAACATTATTACTG
AAGCTACTATTATGAGACCAGCTGAGGTTGGTTATAGTGCACCATATTATTCT
TTTGAGGCGTCTACACAAGGGCCATTTAAAACACCTATTGCAGCAGGACGG
GGGGGAGCGCAAACAGATGAAAATCAAGCAGCAGATGGTGATCCAAGATA
TGCATTTGGTAGACAACATGGTCAAAAAACTACCACAACAGGAGAAACAC
CTGAGAGATTTACATATATAGCACATCAAGATACAGGAAGATATCCAGAAGG
AGATTGGATTCAAAATATTAACTTTAACCTTCCTGTAACAAATGATAATGTAT
TGCTACCAACAGATCCAATTGGAGGTAAAGCAGGAATTAACTATACTAATAT
ATTTAATACTTATGGTCCTTTAACTGCATTAAATAATGTACCACCAGTTTATC
CAAATGGTCAAATTTGGGATAAAGAATTTGATACTGACTTAAAACCAAGAC
TTCATGTAAATGCACCATTTGTTTGTCAAAATAATTGTCCTGGTCAATTATTT
GTAAAAGTTGCGCCTAATTTAACAAATGAATATGATCCTGATGCATCTGCTA
ATATGTCAAGAATTGTAACTTACTCAGATTTTTGGTGGAAAGGTAAATTAGT
ATTTAAAGCTAAACTAAGAGCCTCTCATACTTGGAATCCAATTCAACAAATG
AGTATTAATGTAGATAACCAATTTAACTATGTACCAAGTAATATTGGAGGTAT
GAAAATTGTATATGAAAAATCTCAACTAGCACCTAGAAAATTATACTAA

>DQ903936|New CPV-2a|2006 China Sichuan|dog

ATGAGTGATGGAGCAGTTCAACCAGACGGTGGTCAGCCTGCTGTCAGAAA
TGAAAGAGCTACAGGATCTGGGAACGGGTCTGGAGGCAGGGGCGGTGGT
GGTTCTGGGGGTGTGGGGATTTCTACGGGTACTTTCAATAATCAGACGGAA
TTTAAATTTTTGGAAAACGGATGGGTGGAAATCACAGCAAACTCAAGCAG
ACTTGTACATTTAAATATGCCAGAAAGTGAAAATTATAGAAGAGTGGTTGTA
AATAATTTGGATAAAACTGCAGTTAACGGAAACATGGCTTTAGATGATACCC
ATGCACAAATTGTAACACCTTGGTCATTGGTTGATGCAAATGCTTGGGGAG
TTTGGTTTAATCCAGGAGATTGGCAACTAATTGTTAATACTATGAGTGAGTT
GCATTTAGTTAGTTTTGAGCAAGAAATTTTTAATGTTGTTTTAAAGACTGTT
TCAGAATCTGCTACTCAGCCACCAACTAAAGTTTATAATAATGATTTAACTA
CATCATTGATGGTTGCATTAGATAGTAATAATACTATGCCATTTACTCCAGCA
GCTATGAGATCTGAGACATTGGGTTTTTATCCATGGAAACCAACCATACCAA
CTCCATGGAGATATTATTTTCAATGGGATAGAACATTAATACCATCTCATACT
GGAACTAGTGGCACACCAACAAATATATACCATGGTACAGATCCAGATGAT
GTTCAATTTTATACTATTGAAAATTCTGTGCCAGTACACTTACTAAGAACAG
GTGATGAATTTGCTACAGGAACATTTTTTTTTGATTGTAAACCATGCAGACT
AACACATACATGCCAAACAAATAGAGCATTGCGCTTACCACCATTTCTAAAT

TCTTTGCCTCAAGCTGAAGGAGGTACTAACTTTGGTTATATAGGAGTTCAAC
AAGATAAAAGACGTGGTGTAACTCAAATGGGAAATACAAACTATATTACTG
AAGCTACTATTATGAGACCAGCTGAGGTTGGTTATAGTGCACCATATTATTCT
TTTGAGGCGTCTACACAAGGGCCATTTAAAACACCTATCGCAGCAGGACGG
GGGGGAGCGCAAACAGATGAAAATCAAGCAGCAGATGGTGATCCAAGATA
TGCATTTGGTAGACAACATGGTCAGAAAACTACCACAACAGGAGAAACAC
CTGAGAGATTTACATATATAGCACATCAAGATACAGGAAGATATCCAGAAGG
AGATTGGATTCAAAATATTAACTTTAACCTTCCTGTAACAAATGATAATGTAT
TGCTACCAACAGATCCAATTGGAGGTAAAACAGGAATTAACTATACTAATAT
ATTTAATACTTATGGTCCTTTAACTGCATTAAATAATGTACCACCAGTTTATC
CAAATGGTCAAATTTGGGATAAAGAATTTGATACTGACTTAAAACCAAGAC
TTCATGTAAATGCACCATTTGTTTGTCAAAATAATTGTCCCGGTCAATTATTT
GTAAAAGTTGCGCCTAATTTAACAAATGAATATGATCCTGATGCATCTGCTA
ATATGTCAAGAATTGTAACTTACTCAGATTTTTGGTGGAAAGGTAAATTAGT
ATTTAAAGCTAAACTAAGAGCCTCTCATACTTGGAATCCAATTCAACAAATG
AGTATTAATGTAGATAACCAATTTAACTATGTACCAAGTAACATTGGAGGTAT
GAAAATTGTATATGAGAAATCTCAACTAGCACCTAGAATACTATATTAA

>MH106698|New CPV-2a|2015 China|dog

ATGAGTGATGGAGCAGTTCAACCAGACGGTGGTCAGCCTGCTGTCAGAAA
TGAAAGAGCTACAGGATCTGGGAACGGGTCTGGAGGCGGGGGTGGTGGTG
GTTCTGGGGGTGTGGGGATTTCTACGGGTACTTTCAATAATCAGACGGAATT
TAAATTTTTGGAAAACGGATGGGTGGAAATCACAGCAAACTCAAGCAGAC
TTGTACATTTAAATATGCCAGAAAGTGAAAATTATAGAAGAGTGGTTGTAAA
TAATTTGGATAAAACTGCAGTTAACGGAAACATGGCTTTAGATGATACCCAT
GCACAAATTGTAACACCTTGGTCATTGGTTGATGCAAATGCTTGGGGAGTT
TGGTTTAATCCAGGAGATTGGCAACTAATTGTTAATACTATGAGTGAGTTGC
ATTTAGTTAGTTTTGAACAAGAAATTTTTAATGTTGTTTTAAAGACTGTTTC
AGAATCTGCTACTCAGCCACCAACTAAAGTTTATAATAATGATTTAACTGCA
TCATTGATGGTTGCATTAGATAGTAATAATACTATGCCATTTACTCCAGCAGC
TATGAGATCTGAGACATTGGGTTTTTATCCATGGAAACCAACCATACCAACT
CCATGGAGATATTATTTTCAATGGGATAGAACATTAATACCATCTCATACTGG
AACTAGTGGCACACCAACAAATATATACCATGGTACAGATCCAGATGATGTT
CAATTTTACACTATTGAAAATTCTGTGCCAGTACACTTACTAAGAACAGGTG
ATGAATTTGCTACAGGAACATTTTATTTTGATTGTAAACCATGTAGACTAAC
ACACACATGGCAAACAAATAGAGCATTGGGCTTACCACCATTTCTAAATTCT
TTGCCTCAAGCTGAAGGAGGTACTAACTTTGGTTATATAGGAGTTCAACAA
GATAAAAGACGTGGTGTAACTCAAATGGGAAATACAAACATTATTACTGAA
GCTACTATTATGAGACCAGCTGAGGTTGGTTATAGTGCACCATATTATTCTTT
TGAGGCGTCTACACAAGGGCCATTTAAAACACCTATTGCAGCAGGACGGG
GGGGAGCGCAAACAGATGAAAATCAAGCAGCAGATGGTGATCCAAGATAT
GCATTTGGTAGACAACATGGTCAAAAAACTACCACAACAGGAGAAACACC
TGAGAGATTTACATATATAGCACATCAAGATACAGGAAGATATCCAGAAGGA
GATTGGATTCAAAATATTAACTTTAACCTTCCTGTAACAAATGATAATGTATT

GCTACCAACAGATCCAATTGGAGGTAAAGCAGGAATTAACTATACTAATATA
TTTAATACTTATGGTCCTTTAACTGCATTAAATAATGTACCACCAGTTTATCC
AAATGGTCAAATTTGGGATAAAGAATTTGATACTGACTTAAAACCAAGACT
TCATGTAAATGCACCATTTGTTTGTCAAAATAATTGTCCTGGTCAATTATTTG
TAAAAGTTGCGCCTAATTTAACAAATGAATATGATCCTGATGCATCTGCTAAT
ATGTCAAGAATTGTAACTTACTCAGATTTTTGGTGGAAAGGTAAATTAGTAT
TTAAAGCTAAACTAAGAGCCTCTCATACTTGGAATCCAATTCAACAAATGA
GTATTAATGTAGATAACCAATTTAACTATGTACCAAGTAATATTGGAGGTATG
AAAATTGTATATGAAAAATCTCAACTAGCACCTAGAAAATTATACTAA

>MZ357121|New CPV-2a|2020 China Sichuan|cat

ATGAGTGATGGAGCAGTTCAACCAGACGGTGGTCAGCCTGCTGTCAGAAA
TGAAAGAGCTACAGGATCTGGGAACGGGTCTGGAGGCGGGGGTGGTGGTG
GTTCTGGGGGTGTGGGGATTTCTACGGGTACTTTCAATAATCAGACGGAATT
TAAATTTTTGGAAAACGGATGGGTGGAAATCACAGCAAACTCAAGCAGAC
TTGTACATTTAAATATGCCAGAAAGTGAAAATTATAGAAGAGTGGTTGTAAA
TAATTTGGATAAAACTGCAGTTAACGGAAACATGGCTTTAGATGATACCCAT
GCACAAATTGTAACACCTTGGTCATTGGTTGATGCAAATGCTTGGGGAGTT
TGGTTTAATCCAGGAGATTGGCAACTAATTGTTAATACTATGAGTGAGTTGC
ATTTAGTTAGTTTTGAACAAGAAATTTTTAATGTTGTTTTAAAGACTGTTTC
AGAATCTGCTACTCAGCCACCAACTAAAGTTTATAATAATGATTTAACTGCA
TCATTGATGGTTGCATTAGATAGTAATAATACTATGCCATTTACTCCAGCAGC
TATGAGATCTGAGACATTGGGTTTTTATCCATGGAAACCAACCATACCAACT
CCATGGAGATATTATTTTCAATGGGATAGAACATTAATACCATCTCATACTGG
AACTAGTGGCACACCAACAAATATATACCATGGTACAGATCCAGATGATGTT
CAATTTTACACTATTGAAAATTCTGTGCCAGTACACTTACTAAGAACAGGTG
ATGAATTTGCTACAGGAACATTTTATTTTGATTGTAAACCATGTAGACTAAC
ACACACATGGCAAACAAATAGAGCATTGGGCTTACCACCATTTCTAAATTCT
TTGCCTCAAGCTGAAGGAGGTACTAACTTTGGTTATATAGGAGTTCAACAA
GATAAAAGACGTGGTGTAACTCAAATGGGAAATACAAACATTATTACTGAA
GCTACTATTATGAGACCAGCTGAGGTTGGTTATAGTGCACCATATTATTCTTT
TGAGGCGTCTACACAAGGGCCATTTAAAACACCTATTGCAGCAGGACGGG
GGGGAGCGCAAACAGATGAAAATCAAGCAGCAGATGGTGATCCAAGATAT
GCATTTGGTAGACAACATGGTCAAAAAACTACCACAACAGGAGAAACACC
TGAGAGATTTACATATATAGCACATCAAGATACAGGAAGATATCCAGAAGGA
GATTGGATTCAAAATATTAACTTTAACCTTCCTGTAACAAATGATAATGTATT
GCTACCAACAGATCCAATTGGAGGTAAAGCAGGAATTAACTATACTAATATA
TTTAATACTTATGGTCCTTTAACTGCATTAAATAATGTACCACCAGTTTATCC
AAATGGTCAAATTTGGGATAAAGAATTTGATACTGACTTAAAACCAAGACT
TCATGTAAATGCACCATTTGTTTGTCAAAATAATTGTCCTGGTCAATTATTTG
TAAAAGTTGCGCCTAATTTAACAAATGAATATGATCCTGATGCATCTGCTAAT
ATGTCAAGAATTGTAACTTACTCAGATTTTTGGTGGAAAGGTAAATTAGTAT
TTAAAGCTAAACTAAGAGCCTCTCATACTTGGAATCCAATTCAACAAATGA
GTATTAATGTAGATAACCAATTTAACTATGTACCAAGTAATATTGGAGGTATG

AAAATTGTATATGAAAAATCTCAACTAGCACCTAGAAAATTATACTAA

>JF414817|CPV-2b|2003 Argentina|dog

ATGAGTGATGGAGCAGTACAACCAGACGGTGGTCAACCTGCTGTCAGAAA
TGAAAGAGCTACAGGATCTGGGAACGGGTCTGGAGGCGGGGGTGGTGGTG
GTTCTGGGGGTGTGGGGATTTCTACGGGTACTTTCAATAATCAGACGGAATT
TAAATTTTTGGAAAACGGATGGGTGGAAATCACAGCAAACTCAAGCAGAC
TTGTACATTTAAATATGCCAGAAAGTGAAAATTATAGAAGAGTGGTTGTAAA
TAATTTGGATAAAACTGCAGTTAACGGAAACATGGCTTTAGATGATACTCAT
GCACAAATTGTAACACCTTGGTCATTGGTTGATGCAAATGCTTGGGGAGTT
TGGTTTAATCCAGGAGATTGGCAACTAATTGTTAATACTATGAGTGAATTGC
ATTTAGTTAGTTTTGAACAAGAAATTTTCAATGTTGTTTTAAAGACTGTTTC
AGAATCTGCTACTCAGCCACCAACTAAAGTTTATAATAATGATTTAACTGCA
TCATTGATGGTTGCATTAGATAGTAATAATACTATGCCATTTACTCCAGCAGC
TATGAGATCTGAGACATTGGGTTTTTATCCATGGAAACCAACCATACCAACT
CCATGGAGATATTATTTTCAATGGGATAGAACATTAATACCATCTCATACTGG
AACTAGTGGCACACCAACAAATATATACCATGGTACAGATCCAGATGATGTT
CAATTTTATACTATTGAAAATTCTGTGCCAGTACACTTACTAAGAACAGGTG
ATGAATTTGCTACAGGAACATTTTTTTTTGATTGTAAACCATGTAGACTAAC
ACATACATGGCAAACAAATAGAGCATTGGGCTTACCACCATTTCTAAATTCT
TTGCCTCAAAATGAAGGAGGTACTAACTTTGGTTATATAGGAGTTCAACAA
GATAAAAGACGTGGTGTAACTCAAATGGGAAATACAAACTATATTACTGAA
GCTACTATTATGAGACCAGCTGAGGTTGGTTATAGTGCACCATATTATTCTTT
TGAGGCGTCTACACAAGGGCCATTTAAAACACCTATTGCAGCAGGACGGG
GGGGAGCGCAAACAGATGAAAATCAAGCAGCAGATGGTGATCCAAGATAT
GCATTTGGTAGACAACATGGCCAAAAAACTACCACAACAGGAGAAACACC
TGAGAGATTTACATATATAGCACATCAAGATACAGGAAGATACCCAGAAGG
AGATTGGATTCAAAATATTAACTTTAACCTTCCTGTAACAGATGATAATGTAT
TGCTACCAACAGATCCAATTGGAGGTAAAACAGGAATTAACTATACTAATAT
ATTTAATACTTATGGTCCTTTAACTGCATTAAATAATGTACCACCAGTTTATC
CAAATGGTCAAATTTGGGATAAAGAATTTGATACTGACTTAAAACCAAGAC
TTCATGTAAATGCACCATTTGTTTGTCAAAATAATTGTCCTGGTCAATTATTT
GTAAAAGTTGCGCCTAATTTAACAAATGAATATGATCCTGATGCATCTGCTA
ATATGTCAAGAATTGTAACTTACTCAGATTTTTGGTGGAAAGGTAAATTAGT
ATTTAAAGCTAAACTAAGAGCCTCTCATACTTGGAATCCAATTCAACAAATG
AGTATTAATGTAGATAACCAATTTAACTATGTACCAAGTAATATTGGAGGTAT
GAAAATTGTATATGAAAAATCTCAACTAGCACCTAGAAAATTATATTAA

>MN259054|CPV-2b|2019 Australia|dog

ATGAGTGATGGAGCAGTTCAACCAGACGGTGGTCAACCTGCTGTCAGAAA
TGAAAGAGCTACAGGATCTGGGAACGGGTCTGGAGGCGGGGGTGGTGGTG
GTTCTGGGGGTGTGGGGATTTCTACGGGTACTTTCAATAATCAGACGGAATT
TAAATTTTTGGAAAACGGATGGGTGGAAATCACAGCAAACTCAAGCAGAC
TTGTACATTTAAATATGCCAGAAAGTGAAAATTATAGAAGAGTGGTTGTAAA

TAATTTGGATAAAACTGCAGTTAACGGAAACATGGCTTTAGATGATACTCAT
GCACAAATTGTAACACCTTGGTCATTGGTTGATGCAAATGCTTGGGGAGTT
TGGTTTAATCCAGGAGATTGGCAACTAATTGTTAATACTATGAGTGAGTTGC
ATTTAGTTAGTTTTGAACAAGAAATTTTTAATGTTGTTTTAAAGACTGTTTC
AGAATCTGCTACTCAGCCACCAACTAAAGTTTATAATAATGATTTAACTGCA
TCATTGATGGTTGCATTAGATAGTAATAATACTATGCCATTTACTCCAGCAGC
TATGAGATCTGAGACATTGGGTTTTTATCCATGGAAACCAACCATACCAACT
CCATGGAGATATTATTTTCAATGGGATAGAACATTAATACCATCTCATACTGG
AACTAGTGGCACACCAACAAATATATACCATGGTACAGATCCAGATGATGTT
CAATTTTATACTATTGAAAATTCTGTGCCAGTACACTTACTAAGAACAGGTG
ATGAATTTGCTACAGGAACATTTTTTTTTGATTGTAAACCATGTAGACTAAC
ACATACATGGCAAACAAATAGAGCATTGGGCTTACCACCATTTCTAAATTCT
TTGCCTCAAGCTGAAGGAGGTACTAACTTTGGTTATATAGGAGTTCAACAA
GATAAAAGACGTGGTGTAACTCAAATGGGAAAAACAAACTATATTACTGAA
GCTACTATTATGAGACCAGCTGAGGTTGGTTATAGTGCACCATATTATTCTTT
TGAGGCGTCTACACAAGGGCCATTTAAAACACCTATTGCAGCAGGACGGG
GGGGAGCGCAAACAGATGAAAATCAAGCAGCAGATGGTGATCCAAGATAT
GCATTTGGTAGACAACATGGTCAAAAAACTACCACAACAGGAGAAACACC
TGAGAGATTTACATATATAGCACATCAAGATACAGGAAGATATCCAGAAGGA
AATTGGATTCAAAATATTAACTTTAACCTTCCTGTAACAGATGATAATGTATT
GCTACCAACAGATCCAATTGGAGGTAAAACAGGAATTAACTATACTAATATA
TTTAATACTTATGGTCCTTTAACTGCATTAAATAATGTACCACCAGTTTATCC
AAATGGTCAAATTTGGGATAAAGAATTTGATACTGACTTAAAACCAAGACT
TCATGTAAATGCACCATTTGTTTGTCAAAATAATTGTCCTGGTCAATTATTTG
TAAAAGTTGCGCCTAATTTAACAAATGAATATGATCCTGATGCATCTGCTAAT
ATGTCAAGAATTGTAACTTACTCAGATTTTTGGTGGAAAGGTAAATTAGTAT
TTAAAGCTAAACTAAGAGCCTCTCATACTTGGAATCCAATTCAACAAATGA
GTATTAATGTAGATAACCAATTTAACTATGTACCAAGTAATATTGGAGGTATG
GAAATTGTATATGAAAGATCTCAACTAGCACCTAGAAAATTATATTAA

>PV296092|CPV-2c|2021 China Guangdong|Malayan pangolin

ATGAGTGATGGAGGAGTTCAACCAGACGGTGGTCAACCTGCTGTCAGAAA
TGAAAGAGCTACAGGATCTGGGAACGGGTCTGGAGGCGGGGGTGGTGGTG
GTTCTGGGGGTGTGGGGATTTCTACGGGTACTTTTAATAATCAGACGGAATT
TAAATTTTTGGAAAACGGATGGGTGGAAATCACAGCAAACTCAAGCAGAC
TTGTGCATTTAAATATGCCAGAAAGTGAAAATTATAGAAGAGTGGTTGTAA
ATAATTTGGATAAAACTGCAGTTAACGGAAACATGGCTTTAGATGATACTCA
TGCACAAATTGTAACACCTTGGTCATTGGTTGATGCAAATGCTTGGGGAGT
TTGGTTTAATCCAGGAGATTGGCAACTAATTGTTAATACTATGAGTGAGTTG
CATTTAGTTAGTTTTGAACAAGAAATTTTTAATGTTGTTTTAAAGACTGTTT
CAGAATCTGCTACTCAGCCACCAACTAAAGTTTATAATAATGATTTAACTGC
ATCATTGATGGTTGCATTAGATAGTAATAATACTATGCCATTTACTCCAGCAG
CTATGAGATCTGAGACATTGGGTTTTTATCCATGGAAACCAACCATACCAAC
TCCATGGAGATATTATTTTCAATGGGATAGAACATTAATACCATCTCATACTG

GAACTAGTGGCACACCAACAAATATATACCATGGTACAGATCCAGATGATGT
TCAATTTTACACTATTGAAAATTCTGTGCCAGTACACTTACTAAGAACAGGT
GATGAATTTGCTACAGGAACATTTTATTTTGATTGTAAACCATGTAGACTAA
CACATACATGGCAAACAAATAGAGCATTGGGCTTACCACCATTTCTAAATTC
TTTGCCTCAAGCTGAAGGAGGTACTAACTTTGGTTATATAGGAGTTCAACA
AGATAAAAGACGTGGTGTAACTCAAATGGGAAACACAAACATTATTACTGA
AGCTACTATTATGAGACCAGCTGAGGTTGGTTATAGTGCACCATATTATTCTT
TTGAGGCGTCTACACAAGGGCCATTTAAAACACCTATTGCAGCAGGACGGG
GGGGAGCGCAAACAGATGAAAATCGAGCAGCAGATGGTGATCCAAGATAT
GCATTTGGTAGACAACATGGTCAAAAAACTACCACAACAGGAGAAACACC
TGAGAGATTTACATATATAGCACATCAAGATACAGGAAGATATCCAGAAGGA
GATTGGATTCAAAATATTAACTTTAACCTTCCTGTAACAGAAGATAATGTATT
GCTACCAACAGATCCAATTGGAGGTAAAACAGGAATTAACTATACTAATATA
TTTAATACTTATGGTCCTTTAACTGCATTAAATAATGTACCACCAGTTTATCC
AAATGGTCAAATTTGGGATAAAGAATTTGATACTGACTTAAAACCAAGACT
TCATGTAAATGCACCATTTGTTTGTCAAAATAATTGTCCTGGTCAATTATTTG
TAAAAGTTGCACCTAATTTAACAAATGAATATGATCCTGATGCATCTGCTAAT
ATGTCAAGAATTGTAACTTACTCAGATTTTTGGTGGAAAGGTAAATTAGTAT
TTAAAGCTAAACTAAGAGCCTCTCATACTTGGAATCCAATTCAACAAATGA
GTATCAATGTAGATAACCAATTTAACTATGTACCAAGTAATATTGGAGGTATG
AAAATTGTATATGAAAAATCTCAACTAGCACCTAGAAAATTATATTAA

>OP779651|CPV-2c|2022 China Sichuan|dog

ATGAGTGATGGAGGAGTTCAACCAGACGGTGGTCAGCCTGCTGTCAGAAA
TGAAAGAGCTACAGGATCTGGGAACGGGTCTGGAGGCGGGGGTGGTGGTG
GTTCTGGGGGTGTGGGGATTTCTACGGGTACTTTTAATAATCAGACGGAATT
TAAATTCTTGGAAAACGGATGGGTGGAAATCACAGCAAACTCAAGCAGAC
TTGTGCATTTAAATATGCCAGAAAGTGAAAATTATAGAAGAGTAGTTGTAAA
TAATTTGGATAAAACTGCAGTTAACGGAAACATGGCTTTAGATGATACTCAT
GCACAAATTGTAACACCTTGGTCATTGGTTGATGCAAATGCTTGGGGAGTT
TGGTTTAATCCAGGAGATTGGCAACTAATTGTTAATACTATGAGTGAGTTGC
ATTTAGTTAGTTTTGAACAAGAAATTTTTAATGTTGTTTTAAAGACTGTTTC
AGAATCTGCTACTCAGCCACCAACTAAAGTTTATAATAATGATTTAACTGCA
TCATTGATGGTTGCATTAGATAGTAATAATACTATGCCATTTACTCCAGCAGC
TATGAGATCTGAGACATTGGGTTTTTATCCATGGAAACCAACCATACCAACT
CCATGGAGATATTATTTTCAATGGGATAGAACATTAATACCATCTCATACTGG
AACTAGTGGCACACCAACAAATATATACCATGGTACAGATCCAGATGATGTT
CAATTTTACACTATTGAAAATTCTGTGCCAGTACACTTACTAAGAACAGGTG
ATGAATTTGCTACAGGAACATTTTATTTTGATTGTAAACCATGTAGACTAAC
ACATACATGGCAAACAAATAGAGCATTGGGCTTACCACCATTTCTAAATTCT
TTGCCTCAAGCTGAAGGAGGTACTAACTTTGGTTATATAGGAGTTCAACAA
GATAAAAGACGTGGTGTAACTCAAATGGGAAACACAAACATTATTACTGAA
GCTACTATTATGAGACCAGCTGAGGTTGGTTATAGTGCACCATATTATTCTTT
TGAGGCGTCTACACAAGGGCCATTTAAAACACCTATTGCAGCAGGACGGG

GGGGAGCGCAAACAGATGAAAATCGAGCAGCAGATGGTGATCCAAGATAT
GCATTTGGTAGACAACATGGTCAAAAAACTACCACAACAGGAGAAACACC
TGAGAGATTTACATATATAGCACATCAAGATACAGGAAGATATCCAGAAGGA
GATTGGATTCAAAATATTAACTTTAACCTTCCTGTAACAGAAGATAATGTATT
GCTACCAACAGATCCAATTGGAGGTAAAACAGGAATTAACTATACTAATATA
TTTAATACTTATGGTCCTTTAACTGCATTAAATAATGTACCACCAGTTTATCC
AAATGGTCAAATTTGGGATAAAGAATTTGATACTGACTTAAAACCAAGACT
TCATGTAAATGCACCATTTGTTTGTCAAAATAATTGTCCTGGTCAATTATTTG
TAAAAGTTGCACCTAATTTAACAAATGAATATGATCCTGATGCATCTGCTAAT
ATGTCAAGAATTGTAACTTACTCAGATTTTTGGTGGAAAGGTAAATTAGTAT
TTAAAGCTAAACTAAGAGCCTCTCATACTTGGAATCCAATTCAACAAATGA
GTATCAATGTAGATAACCAATTTAACTATGTACCAAGTAATATTGGAGGTATG
AAAATTGTATATGAAAAATCTCAACTAGCACCTAGAAAATTATATTAA

>MH476583|CPV-2c|2017 China Sichuan|dog

ATGAGTGATGGAGGAGTTCAACCAGACGGTGGTCAACCTGCTGTCAGAAA
TGAAAGAGCTACAGGATCTGGGAACGGGTCTGGAGGCGGGGGTGGTGGTG
GTTCTGGGGGTGTGGGGATTTCTACGGGTACTTTTAATAATCAGACGGAATT
TAAATTTTTGGAAAACGGATGGGTGGAAATCACAGCAAACTCAAGCAGAC
TTGTGCATTTAAATATGCCAGAAAGTGAAAATTATAGAAGAGTGGTTGTAA
ATAATTTGGATAAAACTGCAGTTAACGGAAACATGGCTTTAGATGATACTCA
TGCACAAATTGTAACACCTTGGTCATTGGTTGATGCAAATGCTTGGGGAGT
TTGGTTTAATCCAGGAGATTGGCAACTAATTGTTAATACTATGAGTGAGTTG
CATTTAGTTAGTTTTGAACAAGAAATTTTTAATGTTGTTTTAAAGACTGTTT
CAGAATCTGCTACTCAGCCACCAACTAAAGTTTATAATAATGATTTAACTGC
ATCATTGATGGTTGCATTAGATAGTAATAATACTATGCCATTTACTCCAGCAG
CTATGAGATCTGAGACATTGGGTTTTTATCCATGGAAACCAACCATACCAAC
TCCATGGAGATATTATTTTCAATGGGATAGAACATTAATACCATCTCATACTG
GAACTAGTGGCACACCAACAAATATATACCATGGTACAGATCCAGATGATGT
TCAATTTTACACTATTGAAAATTCTGTGCCAGTACACTTACTAAGAACAGGT
GATGAATTTGCTACAGGAACATTTTATTTTGATTGTAAACCATGTAGACTAA
CACATACATGGCAAACAAATAGAGCATTGGGCTTACCACCATTTCTAAATTC
TTTGCCTCAAGCTGAAGGAGGTACTAACTTTGGTTATATAGGAGTTCAACA
AGATAAAAGACGTGGTGTAACTCAAATGGGAAACACAAACATTATTACTGA
AGCTACTATTATGAGACCAGCTGAGGTTGGTTATAGTGCACCATATTATTCTT
TTGAGGCGTCTACACAAGGGCCATTTAAAACACCTATTGCAGCAGGACGGG
GGGGAGCGCAAACAGATGAAAATCGAGCAGCAGATGGTGATCCAAGATAT
GCATTTGGTAGACAACATGGTCAAAAAACTACCACAACAGGAGAAACACC
TGAGAGATTTACATATATAGCACATCAAGATACAGGAAGATATCCAGAAGGA
GATTGGATTCAAAATATTAACTTTAACCTTCCTGTAACAGAAGATAATGTATT
GCTACCAACAGATCCAATTGGAGGTAAAACAGGAATTAACTATACTAATATA
TTTAATACTTATGGTCCTTTAACTGCATTAAATAATGTACCACCAGTTTATCC
AAATGGTCAAATTTGGGATAAAGAATTTGATACTGACTTAAAACCAAGACT
TCATGTAAATGCACCATTTGTTTGTCAAAATAATTGTCCTGGTCAATTATTTG

TAAAAGTTGCACCTAATTTAACAAATGAATATGATCCTGATGCATCTGCTAAT
ATGTCAAGAATTGTAACTTACTCAGATTTTTGGTGGAAAGGTAAATTAGTAT
TTAAAGCTAAACTAAGAGCCTCTCATACTTGGAATCCAATTCAACAAATGA
GTATCAATGTAGATAACCAATTTAACTATGTACCAAGTAATATTGGAGGTATG
AAAATTGTATATGAAAAATCTCAACTAGCACCTAGAAAATTATATTAA

>MW182700|CPV-2c|2018 China Tianjin|dog

ATGAGTGATGGAGGAGTTCAACCAGACGGTGGTCAACCTGCTGTCAGAAA
TGAAAGAGCTACAGGATCTGGGAACGGGTCTGGAGGCGGGGGTGGTGGTG
GTTCTGGGGGTGTGGGGATTTCTACGGGTACTTTTAATAATCAGACGGAATT
TAAATTTTTGGAAAACGGATGGGTGGAAATCACAGCAAACTCAAGCAGAC
TTGTGCATTTAAATATGCCAGAAAGTGAAAATTATAGAAGAGTGGTTGTAA
ATAATTTGGATAAAACTGCAGTTAACGGAAACATGGCTTTAGATGATACTCA
TGCACAAATTGTAACACCTTGGTCATTGGTTGATGCAAATGCTTGGGGAGT
TTGGTTTAATCCAGGAGATTGGCAATTAATTGTTAATACTATGAGTGAGTTG
CATTTAGTTAGTTTTGAACAAGAAATTTTTAATGTTGTTTTAAAGACTGTTT
CAGAATCTGCTACTCAGCCACCAACTAAAGTTTATAATAATGATTTAACTGC
ATCATTGATGGTTGCATTAGATAGTAATAATACTATGCCATTTACTCCAGCAG
CTATGAGATCTGAGACATTGGGTTTTTATCCATGGAAACCAACCATACCAAC
TCCATGGAGATATTATTTTCAATGGGATAGAACATTAATACCATCTCATACTG
GAACTAGTGGCACACCAACAAATATATACCATGGTACAGATCCAGATGATGT
TCAATTTTACACTATTGAAAATTCTGTGCCAGTACACTTACTAAGAACAGGT
GATGAATTTGCTACAGGAACATTTTATTTTGATTGTAAACCATGCAGACTAA
CACATACATGGCAAACAAATAGAGCATTGGGCTTACCACCATTTCTAAATTC
TTTGCCTCAAGCTGAAGGAGGTACTAACTTTGGTTATATAGGAGTTCAACA
AGATAAAAGACGTGGTGTAACTCAAATGGGAAACACAAACATTATTACTGA
AGCTACTATTATGAGACCAGCTGAGGTTGGTTATAGTGCACCATATTATTCTT
TTGAGGCGTCTACACAAGGGCCATTTAAAACACCTATTGCAGCAGGACGGG
GGGGAGCGCAAACAGATGAAAATCGAGCAGCAGATGGTGATCCAAGATAT
GCATTTGGTAGACAACATGGTCAAAAAACTACCACAACAGGAGAAACACC
TGAGAGATTTACATATATAGCACATCAAGATACAGGAAGATATCCAGAAGGA
GATTGGATTCAAAATATTAACTTTAACCTTCCTGTAACAGAAGATAATGTATT
GCTACCAACAGATCCAATTGGAGGTAAAACAGGAATTAACTATACTAATATA
TTTAATACTTATGGTCCTTTAACTGCATTAAATAATGTACCACCAGTTTATCC
AAATGGTCAAATTTGGGATAAAGAATTTGATACTGACTTAAAACCAAGACT
TCATGTAAATGCACCATTTGTTTGTCAAAATAATTGTCCTGGTCAATTATTTG
TAAAAGTTGCACCTAATTTAACAAATGAATATGATCCTGATGCATCTGCTAAT
ATGTCAAGAATTGTAACTTACTCAGATTTTTGGTGGAAAGGTAAATTAGTAT
TTAAAGCTAAACTAAGAGCCTCTCATACTTGGAATCCAATTCAACAAATGA
GTATCAATGTAGATAACCAATTTAACTATGTACCAAGTAATATTGGAGGTATG
AAAATTGTATATGAAAAATCTCAACTAGCACCTAGAAAATTATATTAA

>PQ604667|CPV-2c|2023 South Korea|dog

ATGAGTGATGGAGCAGTTCAACCAGACGGTGGTCAGCCTGCTGTCAGAAA

TGAAAGAGCTACAGGATCTGGGAACGGGTCTGGAGGCGGGGGTGGTGGTG
GTTCTGGGGGTGTGGGGATTTCTACGGGTACTTTCAATAATCAGACGGAATT
TAAATTTTTGGAAAACGGATGGGTGGAAATCACAGCAAACTCAAGCAGAC
TTGTACATTTAAATATGCCAGAAAGTGAAAATTATAGAAGAGTGRWTGTAA
ATAATTTGGATAAAACTGCAGTTAACGGAAACATGGCTTTAGATGATACCCA
TGCACAAATTGTAACGCCTTGGTCATTGGTTGATGCAAATGCTTGGGGAGT
TTGGTTTAATCCAGGAGATTGGCAACTAATTGTTAATACTATGAGTGAGTTG
CATTTAGTTAGTTTTGAACAAGAAATTTTTAATGTTGTTTTAAAGACTGTTT
CAGAATCTGCTACTCAGCCACCAACTAAAGTTTATAATAATGATTTAACTGC
ATCATTGATGGTTGCATTAGATAGTAATAATACTATGCCATTTACTCCAGCAG
CTATGAGATCTGAGACATTGGGTTTTTATCCATGGAAACCAACCATACCAAC
TCCATGGAGATATTATTTTCAATGGGATAGAACACTAATACCATCTCATACTG
GAACTAGTGGCACACCAACAAATATATACCATGGTACAGATCCAGATGATGT
TCAATTTTACACTATTGAAAATTCTGTGCCAGTACACTTACTAAGAACAGGT
GATGAATTTGCTACAGGAACATTTTATTTTGATTGTAAACCATGTAGACTAA
CACACACATGGCAAACAAATAGAGCATTGGGCTTACCACCATTTCTAAATT
CTTTGCCTCAAGCTGAAGGAGGTACTAACTTTGGTTATATAGGAGTTCAAC
AAGATAAAAGACGTGGTGTAACTCAAATGGGAAATACAAACATTATTACTG
AAGCTACTATTATGAGACCAGCTGAGGTTGGTTATAGTGCACCATATTATTCT
TTTGAGGCGTCTACACAAGGGCCATTTAAAACACCTATTGCAGCAGGACGG
GGGGGAGCGCAAACAGATGAAAATCAAGCAGCAGATGGTGATCCAAGATA
TGCATTTGGTAGACAACATGGTCAAAAAACTACCACAACAGGAGAAACAC
CTGAGAGATTTACATATATAGCACATCAAGATACAGGAAGATATCCAGAAGG
AGATTGGATTCAAAATATTAACTTTAACCTTCCTGTAACAGAAGATAATGTA
TTGCTACCAACAGATCCAATTGGAGGTAAAGCAGGAATTAACTATACTAATA
TATTTAATACTTATGGTCCTTTAACTGCATTAAATAATGTACCACCAGTTTATC
CAAATGGTCAAATTTGGGATAAAGAATTTGATACTGACTTAAAACCAAGAC
TTCATGTAAATGCACCATTTGTTTGTCAAAATAATTGTCCTGGTCAATTATTT
GTAAAAGTTGCACCTAATTTAACAAATGAATATGATCCTGATGCATCTGCTA
ATATGTCAAGAATTGTAACTTACTCAGATTTTTGGTGGAAAGGTAAATTAGT
ATTTAAAGCTAAACTAAGAGCCTCTCATACTTGGAATCCAATTCAACAAATG
AGTATCAATGTAGATAACCAATTTAACTATGTACCAAGTAATATTGGAGGTAT
GAAAATTGTATATGAAAAATCTCAACTAGCACCTAGAAAATTATATTAA

>ON323041|CPV-2c|2019 Thailand|dog

ATGAGTGATGGAGGAGTTCAACCAGACGGTGGTCAACCTGCTGTCAGAAA
TGAAAGAGCTACAGGATCTGGGAACGGGTCTGGAGGCGGGGGTGGTGGTG
GTTCTGGGGGTGTGGGGATTTCTACGGGTACTTTTAATAATCAGACGGAATT
TAAATTTTTGGAAAACGGATGGGTGGAAATCACAGCAAACTCAAGCAGAC
TTGTGCATTTGAATATGCCAGAAAGTGAAAATTATAGAAGAGTGGTTGTAA
ATAATTTGGATAAAACTGCAGTTAACGGAAACATGGCTTTAGATGATACTCA
TGCACAAATTGTAACACCTTGGTCATTGGTTGATGCAAATGCTTGGGGAGT
TTGGTTTAATCCAGGAGATTGGCAACTAATTGTTAATACTATGAGTGAGTTG
CATTTAGTTAGTTTTGAACAAGAAATTTTTAATGTTGTTTTAAAGACTGTTT

CAGAATCTGCTACTCAGCCACCAACTAAAGTTTATAATAATGATTTAACTGC
GTCATTGATGGTTGCATTAGATAGTAATAATACTATGCCATTTACTCCAGCAG
CTATGAGATCTGAGACATTGGGTTTTTATCCATGGAAACCAACCATACCAAC
TCCATGGAGATATTATTTTCAATGGGATAGAACATTAATACCATCTCATACTG
GAACTAGTGGCACACCAACAAATATATACCATGGTACAGATCCAGATGATGT
TCAATTTTACACTATTGAAAATTCTGTGCCAGTACACTTACTAAGAACAGGT
GATGAATTTGCTACAGGAACATTTTATTTTGATTGTAAACCATGTAGACTAA
CACATACATGGCAAACAAATAGAGCATTGGGCTTACCACCATTTCTAAATTC
TTTGCCTCAAGCTGAAGGAGGTACTAACTTTGGTTATATAGGAGTCCAACA
AGATAAAAGACGTGGTGTAACTCAAATGGGAAACACAAACATTATTACTGA
AGCTACTATTATGAGACCAGCTGAGGTTGGTTATAGTGCACCATATTATTCTT
TTGAGGCGTCTACACAAGGGCCATTTAAAACACCTATTGCAGCAGGACGGG
GGGGAGCGCAAACAGATGAAAATCGAGCAGCAGATGGTGATCCAAGATAT
GCATTTGGTAGACAACATGGTCAAAAAACTACCACAACAGGAGAAACACC
TGAGAGATTTACATATATAGCACATCAAGATACAGGAAGATATCCAGAAGGA
GATTGGATTCAAAATATTAACTTTAACCTTCCTGTAACAGAAGATAATGTATT
GCTACCAACAGATCCAATTGGAGGTAAAACAGGAATTAACTATACTAATATG
TTTAATACTTATGGTCCTTTAACTGCATTAAATAATGTACCACCAGTTTATCC
AAATGGTCAAATTTGGGATAAAGAATTTGATACTGACTTAAAACCAAGACT
TCATGTAAATGCACCATTTGTTTGTCAAAATAATTGTCCTGGTCAATTATTTG
TAAAAGTTGCACCTAATTTAACAAATGAATATGATCCTGATGCATCTGCTAAT
ATGTCAAGAATTGTAACTTACTCAGATTTTTGGTGGAAAGGTAAATTAGTAT
TTAAAGCTAAACTAAGAGCCTCTCATACTTGGAATCCAATTCAACAAATGA
GTATCAATGTAGATAACCAATTTAACTATGTACCAAGTAATATTGGAGGTATG
AAAATTGTATATGAAAAATCTCAACTAGCACCTAGAAAATTATATTAA

>OK649758|CPV-2c|2020 China Shanghai|dog

ATGAGTGATGGAGGAGTTCAACCAGACGGTGGTCAACCTGCTGTCAGAAA
TGAAAGAGCTACAGGATCTGGGAACGGGTCTGGAGGCGGGGGTGGTGGTG
GTTCTGGGGGTGTGGGGATTTCTACGGGTACTTTTAATAATCAGACGGAATT
TAAATTTTTGGAAAACGGATGGGTGGAAATCACAGCAAACTCAAGCAGAC
TTGTGCATTTAAATATGCCAGAAAGTGAAAATTATAGAAGAGTGGTTGTAA
ATAATTTGGATAAAACTGCAGTTAACGGAAACATGGCTTTAGATGATACTCA
TGCACAAATTGTAACACCTTGGTCATTGGTTGATGCAAATGCTTGGGGAGT
TTGGTTTAATCCAGGAGATTGGCAACTAATTGTTAATACTATGAGTGAGTTG
CATTTAGTTAGTTTTGAACAAGAAATTTTTAATGTTGTTTTAAAGACTGTTT
CAGAATCTGCTACTCAGCCACCAACTAAAGTTTATAATAATGATTTAACTGC
ATCATTGATGGTTGCATTAGATAGTAATAATACTATGCCATTTACTCCAGCAG
CTATGAGATCTGAGACATTGGGTTTTTATCCATGGAAACCAACCATACCAAC
TCCATGGAGATATTATTTTCAATGGGATAGAACATTAATACCATCTCATACTG
GAACTAGTGGCACACCAACAAATATATACCATGGTACAGATCCAGATGATGT
TCAATTTTACACTATTGAAAATTCTGTGCCAGTACACTTACTAAGAACAGGT
GATGAATTTGCTACAGGAACATTTTATTTTGATTGTAAACCATGTAGACTAA
CACATACATGGCAAACAAATAGAGCATTGGGCTTACCACCATTTCTAAATTC

TTTGCCTCAAGCTGAAGGAGGTACTAACTTTGGTTATATAGGAGTTCAACA
AGATAAAAGACGTGGTGTAACTCAAATGGGAAACACAAACATTATTACTGA
AGCTACTATTATGAGACCAGCTGAGGTTGGTTATAGTGCACCATATTATTCTT
TTGAGGCGTCTACACAAGGGCCATTTAAAACACCTATTGCAGCAGGACGGG
GGGGAGCGCAAACAGATGAAAATCGAGCAGCAGATGGTGATCCAAGATAT
GCATTTGGTAGACAACATGGTCAAAAAACTACCACAACAGGAGAAACACC
TGAGAGATTTACATATATAGCACATCAAGATACAGGAAGATATCCAGAAGGA
GATTGGATTCAAAATATTAACTTTAACCTTCCTGTAACAGAAGATAATGTATT
GCTACCAACAGATCCAATTGGAGGTAAAACAGGAATTAACTATACTAATATA
TTTAATACTTATGGTCCTTTAACTGCATTAAATAATGTACCACCAGTTTATCC
AAATGGTCAAATTTGGGATAAAGAATTTGATACTGACTTAAAACCAAGACT
TCATGTAAATGCACCATTTGTTTGTCAAAATAATTGTCCTGGTCAATTATTTG
TAAAAGTTGCACCTAATTTAACAAATGAATATGATCCTGATGCATCTGCTAAT
ATGTCAAGAATTGTAACTTACTCAGATTTTTGGTGGAAAGGTAAATTAGTAT
TTAAAGCTAAACTAAGAGCCTCTCATACTTGGAATCCAATTCAACAAATGA
GTATCAATGTAGATAACCAATTTAACTATGTACCAAGTAATATTGGAGGTATG
AAAATTGTATATGAAAAATCTCAACTAGCACCTAGAAAATTATATTAA

>MW811188|CPV-2c|2020 China Shanghai|dog

ATGAGTGATGGAGGAGTTCAACCAGACGGTGGTCACCCTGCTGTCAGAAA
TGAAAGAGCTACAGGATCTGGGAACGGGTCTGGAGGCGGGGGTGGTGGTG
GTTCTGGGGGTGTGGGGATTTCTACGGGTACTTTTAATAATCAGACGGAATT
TAAATTCTTGGAAAACGGATGGGTGGAAATCACAGCAAACTCAAGCAGAC
TTGTGCATTTAAATATGCCAGAAAGTGAAAATTATAGAAGAGTAGTTGTAAA
TAATTTGGATAAAACTGCAGTTAACGGAAACATGGCTTTAGATGATACTCAT
GCACAAATTGTAACACCTTGGTCATTGGTTGATGCAAATGCTTGGGGAGTT
TGGTTTAATCCAGGAGATTGGCAACTAATTGTTAATACTATGAGTGAGTTGC
ATTTAGTTAGTTTTGAACAAGAAATTTTTAATGTTGTTTTAAAGACTGTTTC
AGAATCTGCTACTCAGCCACCAACTAAAGTTTATAATAATGATTTAACTGCA
TCATTGATGGTTGCATTAGATAGTAATAATACTATGCCATTTACTCCAGCAGC
TATGAGATCTGAGACATTGGGTTTTTATCCATGGAAACCAACCATACCAACT
CCATGGAGATATTATTTTCAATGGGATAGAACATTAATACCATCTCATACTGG
AACTAGTGGCACACCAACAAATATATACCATGGTACAGATCCAGATGATGTT
CAATTTTACACTATTGAAAATTCTGTGCCAGTACACTTACTAAGAACAGGTG
ATGAATTTGCTACAGGAACATTTTATTTTGATTGTAAACCATGTAGACTAAC
ACATACATGGCAAACAAATAGAGCATTGGGCTTACCACCATTTCTAAATTCT
TTGCCTCAAGCTGAAGGAGGTACTAACTTTGGTTATATAGGAGTTCAACAA
GATAAAAGACGTGGTGTAACTCAAATGGGAAACACAAACATTATTACTGAA
GCTACTATTATGAGACCAGCTGAGGTTGGTTATAGTGCACCATATTATTCTTT
TGAGGCGTCTACACAAGGGCCATTTAAAACACCTATTGCAGCAGGACGGG
GGGGAGCGCAAACAGATGAAAATCGAGCAGCAGATGGTGATCCAAGATAT
GCATTTGGTAGACAACATGGTCAAAAAACTACCACAACAGGAGAAACACC
TGAGAGATTTACATATATAGCACATCAAGATACAGGAAGATATCCAGAAGGA
GATTGGATTCAAAATATTAACTTTAACCTTCCTGTAACAGAAGATAATGTATT

GCTACCAACAGATCCAATTGGAGGTAAAACAGGAATTAACTATACTAATATA
TTTAATACTTATGGTCCTTTAACTGCATTAAATAATGTACCACCAGTTTATCC
AAATGGTCAAATTTGGGATAAAGAATTTGATACTGACTTAAAACCAAGACT
TCATGTAAATGCACCATTTGTTTGTCAAAATAATTGTCCTGGTCAATTATTTG
TAAAAGTTGCACCTAATTTAACAAATGAATATGATCCTGATGCATCTGCTAAT
ATGTCAAGAATTGTAACTTACTCAGATTTTTGGTGGAAAGGTAAATTAGTAT
TTAAAGCTAAACTAAGAGCCTCTCATACTTGGAATCCAATTCAACAAATGA
GTATCAATGTAGATAACCAATTTAACTATGTACCAAGTAATATTGGAGGTATG
AAAATTGTATATGAAAAATCTCAACTAGCACCTAGAAAATTATATTAA

>MW017590|CPV-2c|2019 China Shanghai|dog

ATGAGTGATGGAGGAGTTCAACCAGACGGTGGTCAACCTGCTGTCAGAAA
TGAAAGAGCTACAGGATCTGGGAACGGGTCTGGAGGCGGGGGTGGTGGTG
GTTCTGGGGGTGTGGGGATTTCTACGGGTACTTTTAATAATCAGACGGAATT
TAAATTTTTGGAAAACGGATGGGTGGAAATCACAGCAAACTCAAGCAGAC
TTGTGCATTTAAATATGCCAGAAAGTGAAAATTATAGAAGAGTGGTTGTAA
ATAATTTGGATAAAACTGCAGTTAACGGAAACATGGCTTTAGATGATACTCA
TGCACAAATTGTAACACCTTGGTCATTGGTTGATGCAAATGCTTGGGGAGT
TTGGTTTAATCCAGGAGATTGGCAACTAATTGTTAATACTATGAGTGAGTTG
CATTTAGTTAGTTTTGAACAAGAAATTTTTAATGTTGTTTTAAAGACTGTTT
CAGAATCTGCTACTCAGCCACCAACTAAAGTTTATAATAATGATTTAACTGC
ATCATTGATGGTTGCATTAGATAGTAATAATACTATGCCATTTACTCCAGCAG
CTATGAGATCTGAGACATTGGGTTTTTATCCATGGAAACCAACCATACCAAC
TCCATGGAGATATTATTTTCAATGGGATAGAACATTAATACCATCTCATACTG
GAACTAGTGGCACACCAACAAATATATACCATGGTACAGATCCAGATGATGT
TCAATTTTACACTATTGAAAATTCTGTGCCAGTACACTTACTAAGAACAGGT
GATGAATTTGCTACAGGAACATTTTATTTTGATTGTAAACCATGTAGACTAA
CACATACATGGCAAACAAATAGAGCATTGGGCTTACCACCATTTCTAAATTC
TTTGCCTCAAGCTGAAGGAGGTACTAACTTTGGTTATATAGGAGTTCAACA
AGATAAAAGACGTGGTGTAACTCAAATGGGAAACACAAACATTATTACTGA
AGCTACTATTATGAGACCAGCTGAGGTTGGTTATAGTGCACCATATTATTCTT
TTGAGGCGTCTACACAAGGGCCATTTAAAACACCTATTGCAGCAGGACGGG
GGGGAGCGCAAACAGATGAAAATCGAGCAGCAGATGGTGATCCAAGATAT
GCATTTGGTAGACAACATGGTCAAAAAACTACCACAACAGGAGAAACACC
TGAGAGATTTACATATATAGCACATCAAGATACAGGAAGATATCCAGAAGGA
GATTGGATTCAAAATATTAACTTTAACCTTCCTGTAACAGAAGATAATGTATT
GCTACCAACAGATCCAATTGGAGGTAAAACAGGAATTAACTATACTAATATA
TTTAATACTTATGGTCCTTTAACTGCATTAAATAATGTACCACCAGTTTATCC
AAATGGTCAAATTTGGGATAAAGAATTTGATACTGACTTAAAACCAAGACT
TCATGTAAATGCACCATTTGTTTGTCAAAATAATTGTCCTGGTCAATTATTTG
TAAAAGTTGCACCTAATTTAACAAATGAATATGATCCTGATGCATCTGCTAAT
ATGTCAAGAATTGTAACTTACTCAGATTTTTGGTGGAAAGGTAAATTAGTAT
TTAAAGCTAAACTAAGAGCCTCTCATACTTGGAATCCAATTCAACAAATGA
GTATCAATGTAGATAACCAATTTAACTATGTACCAAGTAATATTGGAGGTATG

AAAATTGTATATGAAAAATCTCAACTAGCACCTAGAAAATTATATTAA

>OR120158|CPV-2c|2020 China Sichuan|panda

ATGAGTGATGGAGGAGTTCAACCAGACGGTGGTCAACCTGCTGTCAGAAA
TGAAAGAGCTACAGGATCTGGGAACGGGTCTGGAGGCGGGGGTGGTGGTG
GTTCTGGGGGTGTGGGGATTTCTACGGGTACTTTTAATAATCAGACGGAATT
TAAATTCTTGGAAAACGGATGGGTGGAAATCACAGCAAACTCAAGCAGAC
TTGTGCATTTAAATATGCCAGAAAGTGAAAATTATAGAAGAGTAGTTGTAAA
TAATTTGGATAAAACTGCAGTTAACGGAAACATGGCTTTAGATGATACTCAT
GCACAAATTGTAACACCTTGGTCATTGGTTGATGCAAATGCTTGGGGAGTT
TGGTTTAATCCAGGAGATTGGCAACTAATTGTTAATACTATGAGTGAGTTGC
ATTTAGTTAGTTTTGAACAAGAAATTTTTAATGTTGTTTTAAAGACTGTTTC
AGAATCTGCTACTCAGCCACCAACTAAAGTTTATAATAATGATTTAACTGCA
TCATTGATGGTTGCATTAGATAGTAATAATACTATGCCATTTACTCCAGCAGC
TATGAGATCTGAGACATTGGGTTTTTATCCATGGAAACCAACCATACCAACT
CCATGGAGATATTATTTTCAATGGGATAGAACATTAATACCATCTCATACTGG
AACTAGTGGCACACCAACAAATATATACCATGGTACAGATCCAGATGATGTT
CAATTTTACACTATTGAAAATTCTGTGCCAGTACACTTACTAAGAACAGGTG
ATGAATTTGCTACAGGAACATTTTATTTTGATTGTAAACCATGTAGACTAAC
ACATACATGGCAAACAAATAGAGCATTGGGCTTACCACCATTTCTAAATTCT
TTGCCTCAAGCTGAAGGAGGTACTAACTTTGGTTATATAGGAGTTCAACAA
GATAAAAGACGTGGTGTAACTCAAATGGGAAACACAAACATTATTACTGAA
GCTACTATTATGAGACCAGCTGAGGTTGGTTATAGTGCACCATATTATTCTTT
TGAGGCGTCTACACAAGGGCCATTTAAAACACCTATTGCAGCAGGACGGG
GGGGAGCGCAAACAGATGAAAATCGAGCAGCAGATGGTGATCCAAGATAT
GCATTTGGTAGACAACATGGTCAAAAAACTACCACAACAGGAGAAACACC
TGAGAGATTTACATATATAGCACATCAAGATACAGGAAGATATCCAGAAGGA
GATTGGATTCAAAATATTAACTTTAACCTTCCTGTAACAGAAGATAATGTATT
GCTACCAACAGATCCAATTGGAGGTAAAACAGGAATTAACTATACTAATATA
TTTAATACTTATGGTCCTTTAACTGCATTAAATAATGTACCACCAGTTTATCC
AAATGGTCAAATTTGGGATAAAGAATTTGATACTGACTTAAAACCAAGACT
TCATGTAAATGCACCATTTGTTTGTCAAAATAATTGTCCTGGTCAATTATTTG
TAAAAGTTGCACCTAATTTAACAAATGAATATGATCCTGATGCATCTGCTAAT
ATGTCAAGAATTGTAACTTACTCAGATTTTTGGTGGAAAGGTAAATTAGTAT
TTAAAGCTAAACTAAGAGCCTCTCATACTTGGAATCCAATTCAACAAATGA
GTATCAATGTAGATAACCAATTTAACTATGTACCAAGTAATATTGGAGGTATG
AAAATTGTATATGAAAAATCTCAACTAGCACCTAGAAAATTATAT---

>PV854070|CPV-2c|2021 China Guangxi|cat

ATGAGTGATGGAGCAGTTCAACCAGACGGTGGTCAACCTGCTGTCAGAAA
TGAAAGAGCTACAGGATCTGGGAACGGGTCTGGAGGCGGGGGTGGTGGTG
GTTCTGGGGGTGTGGGGATTTCTACGGGTACTTTTAATAATCAGACGGAATT
TAAATTTTTGGAAAACGGATGGGTGGAAATCACAGCAAACTCAAGCAGAC
TTGTGCATTTAAATATGCCAGAAAGTGAAAATTATAGAAGAGTGGTTGTAA

ATAATTTGGATAAAACTGCAGTTAACGGAAACATGGCTTTAGATGATACTCA
TGCACAAATTGTAACACCTTGGTCATTGGTTGATGCAAATGCTTGGGGAGT
TTGGTTTAATCCAGGAGATTGGCAACTAATTGTTAATACTATGAGTGAGTTG
CATTTAGTTAGTTTTGAACAAGAAATTTTTAATGTTGTTTTAAAGACTGTTT
CAGAATCTGCTACTCAGCCACCAACTAAAGTTTATAATAATGATTTAACTGC
ATCATTGATGGTTGCATTAGATAGTAATAATACTATGCCATTTACTCCAGCAG
CTATGAGATCTGAGACATTGGGTTTTTATCCATGGAAACCAACCATACCAAC
TCCATGGAGATATTATTTTCAATGGGATAGAACATTAATACCATCTCATACTG
GAACTAGTGGCACACCAACAAATATATACCATGGTACAGATCCAGATGATGT
TCAATTTTACACTATTGAAAATTCTGTGCCAGTACACTTACTAAGAACAGGT
GATGAATTTGCTACAGGAACATTTTATTTTGATTGTAAACCATGTAGACTAA
CACATACATGGCAAACAAATAGAGCATTGGGCTTACCACCATTTCTAAATTC
TTTGCCTCAAGCTGAAGGAGGTACTAACTTTGGTTATATAGGAGTTCAACA
AGATAAAAGACGTGGTGTAACTCAAATGGGAAACACAAACATTATTACTGA
AGCTACTATTATGAGACCAGCTGAGGTTGGTTATAGTGCACCATATTATTCTT
TTGAGGCGTCTACACAAGGGCCATTTAAAACACCTATTGCAGCAGGACGGG
GGGGAGCGCAAACAGATGAAAATCGAGCAGCAGATGGTGATCCAAGATAT
GCATTTGGTAGACAACATGGTCAAAAAACTACCACAACAGGAGAAACACC
TGAGAGATTTACATATATAGCACATCAAGATACAGGAAGATATCCAGAAGGA
GATTGGATTCAAAATATTAACTTTAACCTTCCTGTAACAGAAGATAATGTATT
GCTACCAACAGATCCAATTGGAGGTAAAACAGGAATTAACTATACTAATATA
TTTAATACTTATGGTCCTTTAACTGCATTAAATAATGTACCACCAGTTTATCC
AAATGGTCAAATTTGGGATAAAGAATTTGATACTGACTTAAAACCAAGACT
TCATGTAAATGCACCATTTGTTTGTCAAAATAATTGTCCTGGTCAATTATTTG
TAAAAGTTGCACCTAATTTAACAAATGAATATGATCCTGATGCATCTGCTAAT
ATGTCAAGAATTGTAACTTACTCAGATTTTTGGTGGAAAGGTAAATTAGTAT
TTAAAGCTAAACTAAGAGCATCTCATACTTGGAATCCAATTCAACAAATGA
GTATTAATGTAGATAACCAATTTAACTATGTACCAAATAATATTGGAGCTATG
AAAATTGTATATGAAAAATCTCAACTAGCACCTAGAAAATTATATTAA

>PQ724070|CPV-2c|2022 China Henan|dog

ATGAGTGATGGAGCAGTTCAACCAGACGGTGGTCAGCCTGCTGTCAGAAA
TGAAAGAGCTACAGGATCTGGGAACGGGTCTGGAGGCGGGGGTGGTGGTG
GTTCTGGGGGTGTGGGGATTTCTACGGGTACTTTCAATAATCAGACGGAATT
TAAATTTTTGGAAAACGGATGGGTGGAAATCACAGCAAACTCAAGCAGAC
TTGTACATTTAAATATGCCAGAAAGTGAAAATTATAGAAGAGTAGTTGTAAA
TAATTTGGATAAAACTGCAGTTAACGGAAACATGGCTTTAGATGATACCCAT
GCACAAATTGTAACACCTTGGTCATTGGTTGATGCAAATGCTTGGGGAGTT
TGGTTTAATCCAGGAGATTGGCAACTAATTGTTAATACTATGAGTGAGTTGC
ATTTAGTTAGTTTTGAACAAGAAATTTTTAATGTTGTTTTAAAGACTGTTTC
AGAATCTGCTACTCAGCCACCAACTAAAGTTTATAATAATGATTTAACTGCA
TCATTGATGGTTGCATTAGATAGTAATAATACTATGCCATTTACTCCAGCAGC
TATGAGATCTGAGACATTGGGTTTTTATCCATGGAAACCAACCATACCAACT
CCATGGAGATATTATTTTCAATGGGATAGAACATTAATACCATCTCATACTGG

AACTAGTGGCACACCAACAAATATATACCATGGTACAGATCCAGATGATGTT
CAATTTTACACTATTGAAAATTCTGTGCCAGTACACTTACTAAGAACAGGTG
ATGAGTTTGCTACAGGAACATTTTATTTTGATTGTAAACCATGTAGACTAAC
ACATACATGGCAAACAAATAGAGCATTGGGCTTACCACCATTTCTAAATTCT
TTGCCTCAAGCTGAAGGAGGTACTAACTTTGGTTATATAGGAGTTCAACAA
GATAAAAGACGTGGTGTAACTCAAATGGGAAACACAAACATTATTACTGAA
GCTACTATTATGAGACCAGCTGAGGTTGGTTATAGTGCACCATATTATTCTTT
TGAGGCGTCTACACAAGGGCCATTTAAAACACCTATTGCAGCAGGACGGG
GGGGAGCGCAAACAGATGAAAATCGAGCAGCAGATGGTGATCCAAGATAT
GCATTTGGTAGACAACATGGTCAAAAAACTACCACAACAGGAGAAACACC
TGAGAGATTTACATATATAGCACATCAAGATACAGGAAGATATCCAGAAGGA
GATTGGATTCAAAATATTAACTTTAACCTTCCTGTAACAGAAGATAATGTATT
GCTACCAACAGATCCAATTGGAGGTAAAACAGGAATTAACTATACTAATATA
TTTAATACTTATGGTCCTTTAACTGCATTAAATAATGTACCACCAGTTTATCC
AAATGGTCAAATTTGGGATAAAGAATTTGATACTGACTTAAAACCAAGACT
TCATGTAAATGCACCATTTGTTTGTCAAAATAATTGTCCTGGTCAATTATTTG
TAAAAGTTGCACCTAATTTAACAAATGAATATGATCCTGATGCATCTGCTAAT
ATGTCAAGAATTGTAACTTACTCAGATTTTTGGTGGAAAGGTAAATTAGTAT
TTAAAGCTAAACTAAGAGCCTCTCATACTTGGAATCCAATTCAACAAATGA
GTATCAATGTAGATAACCAATTTAACTATGTACCAAGTAATATTGGAGGTATG
AAAATTGTATATGAAAAATCTCAACTAGCACCTAGAAAATTATATTAA

>PQ724066|CPV-2c|2022 China Liaoning|dog

ATGAGTGATGGAGCAGTTCAACCAGACGGTGGTCAGCCTGCTGTCAGAAA
TGAAAGAGCTACAGGATCTGGGAACGGGTCTGGAGGCGGGGGTGGTGGTG
GTTCTGGGGGTGTGGGGATTTCTACGGGTACTTTCAATAATCAGACGGAATT
TAAATTTTTGGAAAACGGATGGGTGGAAATCACAGCAAACTCAAGCAGAC
TTGTACATTTAAATATGCCAGAAAGTGAAAATTATAGAAGAGTAGTTGTAAA
TAATTTGGATAAAACTGCAGTTAACGGAAACATGGCTTTAGATGATACCCAT
GCACAAATTGTAACACCTTGGTCATTGGTTGATGCAAATGCTTGGGGAGTT
TGGTTTAATCCAGGAGATTGGCAACTAATTGTTAATACTATGAGTGAGTTGC
ATTTAGTTAGTTTTGAACAAGAAATTTTTAATGTTGTTTTAAAGACTGTTTC
AGAATCTGCTACTCAGCCACCAACTAAAGTTTATAATAATGATTTAACTGCA
TCATTGATGGTTGCATTAGATAGTAATAATACTATGCCATTTACTCCAGCAGC
TATGAGATCTGAGACATTGGGTTTTTATCCATGGAAACCAACCATACCAACT
CCATGGAGATATTATTTTCAATGGGATAGAACATTAATACCATCTCATACTGG
AACTAGTGGCACACCAACAAATATATACCATGGTACAGATCCAGATGATGTT
CAATTTTACACTATTGAAAATTCTGTGCCAGTACACTTACTAAGAACAGGTG
ATGAGTTTGCTACAGGAACATTTTATTTTGATTGTAAACCATGTAGACTAAC
ACATACATGGCAAACAAATAGAGCATTGGGCTTACCACCATTTCTAAATTCT
TTGCCTCAAGCTGAAGGAGGTACTAACTTTGGTTATATAGGAGTTCAACAA
GATAAAAGACGTGGTGTAACTCAAATGGGAAACACAAACATTATTACTGAA
GCTACTATTATGAGACCAGCTGAGGTTGGTTATAGTGCACCATATTATTCTTT
TGAGGCGTCTACACAAGGGCCATTTAAAACACCTATTGCAGCAGGACGGG

GGGGAGCGCAAACAGATGAAAATCGAGCAGCAGATGGTGATCCAAGATAT
GCATTTGGTAGACAACATGGTCAAAAAACTACCACAACAGGAGAAACACC
TGAGAGATTTACATATATAGCACATCAAGATACAGGAAGATATCCAGAAGGA
GATTGGATTCAAAATATTAACTTTAACCTTCCTGTAACAGAAGATAATGTATT
GCTACCAACAGATCCAATTGGAGGTAAAACAGGAATTAACTATACTAATATA
TTTAATACTTATGGTCCTTTAACTGCATTAAATAATGTACCACCAGTTTATCC
AAATGGTCAAATTTGGGATAAAGAATTTGATACTGACTTAAAACCAAGACT
TCATGTAAATGCACCATTTGTTTGTCAAAATAATTGTCCTGGTCAATTATTTG
TAAAAGTTGCACCTAATTTAACAAATGAATATGATCCTGATGCATCTGCTAAT
ATGTCAAGAATTGTAACTTACTCAGATTTTTGGTGGAAAGGTAAATTAGTAT
TTAAAGCTAAACTAAGAGCCTCTCATACTTGGAATCCAATTCAACAAATGA
GTATCAATGTAGATAACCAATTTAACTATGTACCAAGTAATATTGGAGGTATG
AAAATTGTATATGAAAAATCTCAACTAGCACCTAGAAAATTATATTAA

>MT892649|CPV-2c|2019 China Shaanxi|domestic dog

ATGAGTGATGGAGGAGTTCAACCAGACGGTGGTCAACCTGCTGTCAGAAA
TGAAAGAGCTACAGGATCTGGGAACGGGTCTGGAGGCGGGGGTGGTGGTG
GTTCTGGGGGTGTGGGGATTTCTACGGGTACTTTTAATAATCAGACGGAATT
TAAATTTTTGGAAAACGGATGGGTGGAAATCACAGCAAACTCAAGCAGAC
TTGTACATTTAAATATGCCAGAAAGTGAAAATTATAGAAGAGTGGTTGTAAA
TAATTTGGATAAAACTGCAGTTAACGGAAACATGGCTTTAGATGATACTCAT
GCACAAATTGTAACACCTTGGTCATTGGTTGATGCAAATGCTTGGGGAGTT
TGGTTTAATCCAGGAGATTGGCAACTAATTGTTAATACTATGAGTGAGTTGC
ATTTAGTTAGTTTTGAACAAGAAATTTTTAATGTTGTTTTAAAGACTGTTTC
AGAATCTGCTACTCAGCCACCAACTAAAGTTTATAATAATGATTTAACTGCA
TCATTGATGGTTGCATTAGATAGTAATAATACTATGCCATTTACTCCAGCAGC
TATGAGATCTGAGACATTGGGTTTTTATCCATGGAAACCAACCATACCAACT
CCATGGAGATATTATTTTCAATGGGATAGAACATTAATACCATCTCATACTGG
AACTAGTGGCACACCAACAAATATATACCATGGTACAGATCCAGATGATGTT
CAATTTTACACTATTGAAAATTCTGTGCCAGTACACTTACTAAGAACAGGTG
ATGAATTTGCTACAGGAACATTTTATTTTGATTGTAAACCATGTAGACTAAC
ACATACATGGCAAACAAATAGAGCATTGGGCTTACCACCATTTCTAAATTCT
TTGCCTCAAGCTGAAGGAGGTACTAACTTTGGTTATATAGGAGTTCAACAA
GATAAAAGACGTGGTGTAACTCAAATGGGAAACACAAACATTATTACTGAA
GCTACTATTATGAGACCAGCTGAGGTTGGTTATAGTGCACCATATTATTCTTT
TGAGGCGTCTACACAAGGGCCATTTAAAACACCTATTGCAGCAGGACGGG
GGGGAGCGCAAACAGATGAAAATCAAGCAGCAGATGGTGATCCAAGATAT
GCATTTGGTAGACAACATGGTCAAAAAACTACCACAACAGGAGAAACACC
TGAGAGATTTACATATATAGCACATCAAGATACAGGAAGATATCCAGAAGGA
GATTGGATTCAAAATATTAACTTTAACCTTCCTGTAACAGAAGATAATGTATT
GCTACCAACAGATCCAATTGGAGGTAAAACAGGAATTAACTATACTAATATA
TTTAATACTTATGGTCCTTTAACTGCATTAAATAATGTACCACCAGTTTATCC
AAATGGTCAAATTTGGGATAAAGAATTTGATACTGACTTAAAACCAAGACT
CCATGTAAATGCACCATTTGTTTGTCAAAATAATTGTCCTGGTCAATTATTTG

TAAAAGTTGCACCTAATTTAACAAATGAATATGATCCTGATGCATCTGCTAAT
ATGTCAAGAATTGTAACTTACTCAGATTTTTGGTGGAAAGGTAAATTAGTAT
TTAAAGCTAAACTAAGAGCCTCTCATACTTGGAATCCAATTCAACAAATGA
GTATCAATGTAGATAACCAATTTAACTATGTACCAAGTAATATTGGAGGTATG
AAAATTGCATATGAAAAATCTCAACTAGCACCTAGAAAATTATAT---

>MZ506743|CPV-2c|2019 China Shaanxi|domestic dog

ATGAGTGATGGAGGAGTTCAACCAGACGGTGGTCAACCTGCTGTCAGAAA
TGAAAGAGCTACAGGATCTGGGAACGGGTCTGGAGGCGGGGGTGGTGGTG
GTTCTGGGGGTGTGGGGATTTCTACGGGTACTTTTAATAATCAGACGGAATT
TAAATTTTTGGAAAACGGATGGGTGGAAATCACAGCAAACTCAAGCAGAC
TTGTGCATTTAAATATGCCAGAAAGTGAAAATTATAGAAGAGTGGTTGTAA
ATAATTTGGATAAAACTGCAGTTAACGGAAACATGGCTTTAGATGATACTCA
TGCACAAATTGTAACACCTTGGTCATTGGTTGATGCAAATGCTTGGGGAGT
TTGGTTTAATCCAGGAGATTGGCAACTAATTGTTAATACTATGAGTGAGTTG
CATTTAGTTAGTTTTGAACAAGAAATTTTTAATGTTGTTTTAAAGACTGTTT
CAGAATCTGCTACTCAGCCACCAACTAAAGTTTATAATAATGATTTAACTGC
ATCATTGATGGTTGCATTAGATAGTAATAATACTATGCCATTTACTCCAGCAG
CTATGAGATCTGAGACATTGGGTTTTTATCCATGGAAACCAACCATACCAAC
TCCATGGAGATATTATTTTCAATGGGATAGAACATTAATACCATCTCATACTG
GAACTAGTGGCACACCAACAAATATATACCATGGTACAGATCCAGATGATGT
TCAATTTTACACTATTGAAAATTCTGTGCCAGTACACTTACTAAGAACAGGT
GATGAATTTGCTACAGGAACATTTTATTTTGATTGTAAACCATGTAGACTAA
CACATACATGGCAAACAAATAGAGCATTGGGCTTACCACCATTTCTAAATTC
TTTGCCTCAAGCTGAAGGAGGTACTAACTTTGGTTATATAGGAGTTCAACA
AGATAAAAGACGTGGTGTAACTCAAATGGGAAACACAAACATTATTACTGA
AGCTACTATTATGAGACCAGCTGAGGTTGGTTATAGTGCACCATATTATTCTT
TTGAGGCGTCTACACAAGGGCCATTTAAAACACCTATTGCAGCAGGACGGG
GGGGAGCGCAAACAGATGAAAATCGAGCAGCAGATGGTGATCCAAGATAT
GCATTTGGTAGACAACATGGTCAAAAAACTACCACAACAGGAGAAACACC
TGAGAGATTTACATATATAGCACATCAAGATACAGGAAGATATCCAGAAGGA
GATTGGATTCAAAATATTAACTTTAACCTTCCTGTAACAGAAGATAATGTATT
GCTACCAACAGATCCAATTGGAGGTAAAACAGGAATTAACTATACTAATATA
TTTAATACTTATGGTCCTTTAACTGCATTAAATAATGTACCACCAGTTTATCC
AAATGGTCAAATTTGGGATAAAGAATTTGATACTGACTTAAAACCAAGACT
TCATGTAAATGCACCATTTGTTTGTCAAAATAATTGTCCTGGTCAATTATTTG
TAAAAGTTGCACCTAATTTAACAAATGAATATGATCCTGATGCATCTGCTAAT
ATGTCAAGAATTGTAACTTACTCAGATTTTTGGTGGAAAGGTAAATTAGTAT
TTAAAGCTAAACTAAGAGCCTCTCATACTTGGAATCCAATTCAACAAATGA
GTATCAATGTAGATAACCAATTTAACTATGTACCAAGTAATATTGGAGGTATG
AAAATTGTATATGAAAAATCTCAACTAGCACCTAGAAAATTATAT---

>MZ614967|CPV-2c|2019 China Shaanxi|domestic dog

ATGAGTGATGGAGGAGTTCAACCAGACGGTGGTCAGCCTACTGTCAGAAA

TGAAAGAGCTACAGGATCTGGGAACGGGTCTGGAGGCGGGGGTGGTGGTG
GTTCTGGGGGTGTGGGGATTTCTACGGGTACTTTCAATAATCAGACGGAATT
TAAATTTTTGGAAAACGGATGGGTGGAAATCACAGCAAACTCAAGCAGAC
TTGTACATTTAAATATGCCAGAAAGTGAAAATTATAGAAGAGTGGTTGTAAA
TAATTTGGATAAAACTGCAGTTAACGGAAACATGGCTTTAGATGATACCCAT
GCACAAATTGTAACACCTTGGTCATTGGTTGATGCAAATGCTTGGGGAGTT
TGGTTTAATCCAGGAGATTGGCAACTAATTGTTAATACTATGAGTGAGTTGC
ATTTAGTTAGTTTTGAACAAGAAATTTTTAATGTTGTTTTAAAGACTGTTTC
AGAATCTGCTACTCAGCCACCAACTAAAGTTTATAATAATGATTTAACTGCA
TCATTGATGGTTGCATTAGATAGTAATAATACTATGCCATTTACTCCAGCAGC
TATGAGATCTGAGACATTGGGTTTTTATCCATGGAAACCAACCATACCAACT
CCATGGAGATATTATTTTCAATGGGATAGAACATTAATACCATCTCATACTGG
AACTAGTGGCACACCAACAAATATATACCATGGTACAGATCCAGATGATGTT
CAATTTTACACTATTGAAAATTCTGTGCCAGTACACTTACTAAGAACAGGTG
ATGAGTTTGCTACAGGAACATTTTATTTTGATTGTAAACCATGTAGACTAAC
ACATACATGGCAAACAAATAGAGCATTGGGCTTACCACCATTTCTAAATTCT
TTGCCTCAAGCTGAAGGAGGTACTAACTTTGGTTATATAGGAGTTCAACAA
GATAAAAGACGTGGTGTAACTCAAATGGGAAACACAAACATTATTACTGAA
GCTACTATTATGAGACCAGCTGAGGTTGGTTATAGTGCACCATATTATTCTTT
TGAGGCGTCTACACAAGGGCCATTTAAAACACCTATTGCAGCAGGACGGG
GGGGAGCGCAAACAGATGAAAATCGAGCAGCAGATGGTGATCCAAGATAT
GCATTTGGTAGACAACATGGTCAAAAAACTACCACAACAGGAGAAACACC
TGAGAGATTTACATATATAGCACATCAAGATACAGGAAGATATCCAGAAGGA
GATTGGATTCAAAATATTAACTTTAACCTTCCTGTAACAGAAGATAATGTATT
GCTACCAACAGATCCAATTGGAGGTAAAACAGGAATTAACTATACTAATATA
TTTAATACTTATGGTCCTTTAACTGCATTAAATAATGTACCACCAGTTTATCC
AAATGGTCAAATTTGGGATAAAGAATTTGATACTGACTTAAAACCAAGACT
TCATGTAAATGCACCATTTGTTTGTCAAAATAATTGTCCTGGTCAATTATTTG
TAAAAGTTGCACCTAATTTAACAAATGAATATGATCCTGATGCATCTGCTAAT
ATGTCAAGAATTGTAACTTACTCAGATTTTTGGTGGAAAGGTAAATTAGTAT
TTAAAGCTAAACTAAGAGCCTCTCATACTTGGAATCCAATTCAACAAATGA
GTATCAATGTAGATAACCAATTTAACTATGTACCAAGTAATATTGGAGGTATG
AAAATTGTATATGAAAAATCTCAACTAGCACCTAGAAAATTATAT---

>MZ614966|CPV-2c|2019 China Shaanxi|domestic dog

ATGAGTGATGGAGCAGTTCAACCAGACGGTGGTCAGCCTGCTGTCAGAAA
TGAAAGAGCTACAGGATCTGGGAACGGGTCTGGAGGCGGGGGTGGTGGTG
GTTCTGGGGGTGTGGGGATTTCTACGGGTACTTTCAATAATCAGACGGAATT
TAAATTTTTGGAAAACGGATGGGTGGAAATCACAGCAAACTCAAGCAGAC
TTGTACATTTAAATATGCCAGAAAGTGAAAATTATAGAAGAGTGGTTGTAAA
TAATTTGGATAAAACTGCAGTTAACGGAAACATGGCTTTAGATGATACCCAT
GCACAAATTGTAACACCTTGGTCATTGGTTGATGCAAATGCTTGGGGAGTT
TGGTTTAATCCAGGAGATTGGCAACTAATTGTTAATACTATGAGTGAGTTGC
ATTTAGTTAGTTTTGAACAAGAAATTTTTAATGTTGTTTTAAAGACTGTTTC

AGAATCTGCTACTCAGCCACCAACTAAAGTTTATAATAATGATTTAACTGCA
TCATTGATGGTTGCATTAGATAGTAATAATACTATGCCATTTACTCCAGCAGC
TATGAGATCTGAGACATTGGGTTTTTATCCATGGAAACCAACCATACCAACT
CCATGGAGATATTATTTTCAATGGGATAGAACATTAATACCATCTCATACTGG
AACTAGTGGCACACCAACAAATATATACCATGGTACAGATCCAGATGATGTT
CAATTTTACACTATTGAAAATTCTGTGCCAGTACACTTACTAAGAACAGGTG
ATGAGTTTGCTACAGGAACATTTTATTTTGATTGTAAACCATGTAGACTAAC
ACATACATGGCAAACAAATAGAGCATTGGGCTTACCACCATTTCTAAATTCT
TTGCCTCAAGCTGAAGGAGGTACTAACTTTGGTTATATAGGAGTTCAACAA
GATAAAAGACGTGGTGTAACTCAAATGGGAAACACAAACATTATTACTGAA
GCTACTATTATGAGACCAGCTGAGGTTGGTTATAGTGCACCATATTATTCTTT
TGAGGCGTCTACACAAGGGCCATTTAAAACACCTATTGCAGCAGGACGGG
GGGGAGCGCAAACAGATGAAAATCGAGCAGCAGATGGTGATCCAAGATAT
GCATTTGGTAGACAACATGGTCAAAAAACTACCACAACAGGAGAAACACC
TGAGAGATTTACATATATAGCACATCAAGATACAGGAAGATATCCAGAAGGA
GATTGGATTCAAAATATTAACTTTAACCTTCCTGTAACAGAAGATAATGTATT
GCTACCAACAGATCCAATTGGAGGTAAAACAGGAATTAACTATACTAATATA
TTTAATACTTATGGTCCTTTAACTGCATTAAATAATGTACCACCAGTTTATCC
AAATGGTCAAATTTGGGATAAAGAATTTGATACTGACTTAAAACCAAGACT
TCATGTAAATGCACCATTTGTTTGTCAAAATAATTGTCCTGGTCAATTATTTG
TAAAAGTTGCACCTAATTTAACAAATGAATATGATCCTGATGCATCTGCTAAT
ATGTCAAGAATTGTAACTTACTCAGATTTTTGGTGGAAAGGTAAATTAGTAT
TTAAAGCTAAACTAAGAGCCTCTCATACTTGGAATCCAATTCAACAAATGA
GTATCAATGTAGATAACCAATTTAACTATGTACCAAGTAATATTGGAGGTATG
AAAATTGTATATGAAAAATCTCAACTAGCACCTAGAAAATTATAT---

>MZ614963|CPV-2c|2019 China Shaanxi|domestic dog

ATGAGTGATGGAGGAGTTCAACCAGACGGTGGTCAACCTGCTGTCAGAAA
TGAAAGAGCTACAGGATCTGGGAACGGGTCTGGAGGCGGGGGTGGTGGTG
GTTCTGGGGGTGTGGGGATTTCTACGGGTACTTTTAATAATCAGACGGAATT
TAAATTTTTGGAAAACGGATGGGTGGAAATCACAGCAAACTCAAGCAGAC
TTGTGCATTTAAATATGCCAGAAAGTGAAAATTATAGAAGAGTGGTTGTAA
ATAATTTGGATAAAACTGCAGTTAACGGAAACATGGCTTTAGATGATACTCA
TGCACAAATTGTAACACCTTGGTCATTGGTTGATGCAAATGCTTGGGGAGT
TTGGTTTAATCCAGGAGATTGGCAACTAATTGTTAATACTATGAGTGAGTTG
CATTTAGTTAGTTTTGAACAAGAAATTTTTAATGTTGTTTTAAAGACTGTTT
CAGAATCTGCTACTCAGCCACCAACTAAAGTTTATAATAATGATTTAACTGC
ATCATTGATGGTTGCATTAGATAGTAATAATACTATGCCATTTACTCCAGCAG
CTATGAGATCTGAGACATTGGGTTTTTATCCATGGAAACCAACCATACCAAC
TCCATGGAGATATTATTTTCAATGGGATAGAACATTAATACCATCTCATACTG
GAACTAGTGGCACACCAACAAATATATACCATGGTACAGATCCAGATGATGT
TCAATTTTACACCATTGAAAATTCTGTACCAGTACACTTACTAAGAACAGGT
GATGAATTTGCTACAGGAACATTTTATTTTGATTGTAAACCATGTAGACTAA
CACATACATGGCAAACAAATAGAGCATTGGGCTTACCACCATTTCTAAATTC

TTTGCCTCAAGCTGAAGGAGGTACTAACTTTGGTTATATAGGAGTTCAACA
AGATAAAAGACGTGGTGTAACTCAAATGGGAAACACAAACATTATTACTGA
AGCTACTATTATGAGACCAGCTGAGGTTGGTTATAGTGCACCATATTATTCTT
TTGAGGCGTCTACACAAGGGCCATTTAAAACACCTATTGCAGCAGGACGGG
GGGGAGCGCAAACAGATGAAAATCGAGCAGCAGATGGTGATCCAAGATAT
GCATTTGGTAGACAACATGGTCAAAAAACTACCACAACAGGAGAAACACC
TGAGAGATTTACATATATAGCACATCAAGATACAGGAAGATATCCAGAAGGA
GATTGGATTCAAAATATTAACTTTAACCTTCCTGTAACAGAAGATAATGTATT
GCTACCAACAGATCCAATTGGAGGTAAAACAGGAATTAACTATACTAATATA
TTTAATACTTATGGTCCTTTAACTGCATTAAATAATGTACCACCAGTTTATCC
AAATGGTCAAATTTGGGATAAAGAATTTGATACTGACTTAAAACCAAGACT
TCATGTAAATGCACCATTTGTTTGTCAAAATAATTGTCCTGGTCAATTATTTG
TAAAAGTTGCACCTAATTTAACAAATGAATATGATCCTGATGCATCTGCTAAT
ATGTCAAGAATTGTAACTTACTCAGATTTTTGGTGGAAAGGTAAATTAGTAT
TTAAAGCTAAACTAAGAGCCTCTCATACTTGGAATCCAATTCAACAAATGA
GTATCAATGTAGATAACCAATTTAACTATGTACCAAGTAATATTGGAGGTATG
AAAATTGTATATGAAAAATCTCAACTAGCACCTAGAAAATTATAT---

>MZ614965|CPV-2c|2019 China Shaanxi|domestic dog

ATGAGTGATGGAGGAGTTCAACCAGACGGTGGTCAACCTGCTGTCAGAAA
TGAAAGAGCTACAGGATCTGGGAACGGGTCTGGAGGCGGGGGTGGTGGTG
GTTCTGGGGGTGTGGGGATTTCTACGGGTACTTTTAATAATCAGACGGAATT
TAAATTCTTGGAAAACGGATGGGTGGAAATCACAGCAAACTCAAGCAGAC
TTGTGCATTTAAATATGCCAGAAAGTGAAAATTATAGAAGAGTAGTTGTAAA
TAATTTGGATAAAACTGCAGTTAACGGAAACATGGCTTTAGATGATACTCAT
GCACAAATTGTAACACCTTGGTCATTGGTTGATGCAAATGCTTGGGGAGTT
TGGTTTAATCCAGGAGATTGGCAACTAATTGTTAATACTATGAGTGAGTTGC
ATTTAGTTAGTTTTGAACAAGAAATTTTTAATGTTGTTTTAAAGACTGTTTC
AGAATCTGCTACTCAGCCACCAACTAAAGTTTATAATAATGATTTAACTGCA
TCATTGATGGTTGCATTAGATAGTAATAATACTATGCCATTTACTCCAGCAGC
TATGAGATCTGAGACATTGGGTTTTTATCCATGGAAACCAACCATACCAACT
CCATGGAGATATTATTTTCAATGGGATAGAACATTAATACCATCTCATACTGG
AACTAGTGGCACACCAACAAATATATACCATGGTACAGATCCAGATGATGTT
CAATTTTACACTATTGAAAATTCTGTGCCAGTACACTTACTAAGAACAGGTG
ATGAATTTGCTACAGGAACATTTTATTTTGATTGTAAACCATGTAGACTAAC
ACATACATGGCAAACAAATAGAGCATTGGGCTTACCACCATTTCTAAATTCT
TTGCCTCAAGCTGAAGGAGGTACTAACTTTGGTTATATAGGAGTTCAACAA
GATAAAAGACGTGGTGTAACTCAAATGGGAAACACAAATATTATTACTGAA
GCTACTATTATGAGACCAGCTGAGGTTGGTTATAGTGCACCATATTATTCTTT
TGAGGCGTCTACACAAGGGCCATTTAAAACACCTATTGCAGCAGGACGGG
GGGGAGCGCAAACAGATGAAAATCGAGCAGCAGATGGTGATCCAAGATAT
GCATTTGGTAGACAACATGGTCAAAAAACTACCACAACAGGAGAAACACC
TGAGAGATTTACATATATAGCACATCAAGATACAGGAAGATATCCAGAAGGA
GATTGGATTCAAAATATTAACTTTAACCTTCCTGTAACAGAAGATAATGTATT

GCTACCAACAGATCCAATTGGAGGTAAAACAGGAATTAACTATACTAATATA
TTTAATACTTATGGTCCTTTAACTGCATTAAATAATGTACCACCAGTTTATCC
AAATGGTCAAATTTGGGATAAAGAATTTGATACTGACCTAAAACCAAGACT
TCATGTAAATGCACCATTTGTTTGTCAAAATAATTGTCCTGGTCAATTATTTG
TAAAAGTTGCACCTAATTTAACAAATGAATATGATCCTGATGCATCTGCTAAT
ATGTCAAGAATTGTAACTTACTCAGATTTTTGGTGGAAAGGTAAATTAGTAT
TTAAAGCTAAACTAAGAGCCTCTCATACTTGGAATCCAATTCAACAAATGA
GTATCAATGTAGATAACCAATTTAACTATGTACCAAGTAATATTGGAGGTATG
AAAATTGTATATGAAAAATCTCAACTAGCACCTAGAAAATTATAT---

>MZ614964|CPV-2c|2019 China Shaanxi|domestic dog

ATGAGTGATGGAGGAGTTCAACCAGACGGTGGTCAACCTGCTGTCAGAAA
TGAAAGAGCTACAGGATCTGGGAACGGGTCTGGAGGCGGGGGTGGTGGTG
GTTCTGGGGGTGTGGGGATTTCTACGGGTACTTTTAATAATCAGACGGAATT
TAAATTTTTGGAAAACGGATGGGTGGAAATCACAGCAAACTCAAGCAGAC
TTGTGCATTTAAATATGCCAGAAAGTGAAAATTATAGAAGAGTGGTTGTAA
ATAATTTGGATAAAACTGCAGTTAACGGAAACATGGCTTTAGATGATACTCA
TGCACAAATTGTAACACCTTGGTCATTGGTTGATGCAAATGCTTGGGGAGT
TTGGTTTAATCCAGGAGATTGGCAACTAATTGTTAATACTATGAGTGAGTTG
CATTTAGTTAGTTTTGAACAAGAAATTTTTAATGTTGTTTTAAAGACTGTTT
CAGAATCTGCTACTCAGCCACCAACTAAAGTTTATAATAATGATTTAACTGC
ATCATTGATGGTTGCATTAGATAGTAATAATACTATGCCATTTACTCCAGCAG
CTATGAGATCTGAGACATTGGGTTTTTATCCATGGAAACCAACCATACCAAC
TCCATGGAGATATTATTTTCAATGGGATAGAACATTAATACCATCTCATACTG
GAACTAGTGGCACACCAACAAATATATACCATGGTACAGATCCAGATGATGT
TCAATTTTACACTATTGAAAATTCTGTGCCAGTACACTTACTAAGAACAGGT
GATGAATTTGCTACAGGAACATTTTATTTTGATTGTAAACCATGTAGACTAA
CACATACATGGCAAACAAATAGAGCATTGGGCTTACCACCATTTCTAAATTC
TTTGCCTCAAGCTGAAGGAGGTACTAACTTTGGTTATATAGGAGTTCAACA
AGATAAAAGACGTGGTGTAACTCAAATGGGAAACACAAACATTATTACTGA
AGCTACTATTATGAGACCAGCTGAGGTTGGTTATAGTGCACCATATTATTCTT
TTGAGGCGTCTACACAAGGGCCATTTAAAACACCTATTGCAGCAGGACGGG
GGGGAGCGCAAACAGATGAAAATCGAGCAGCAGATGGTGATCCAAGATAT
GCATTTGGTAGACAACATGGTCAAAAAACTACCACAACAGGAGAAACACC
TGAGAGATTTACATATATAGCACATCAAGATACAGGAAGATATCCAGAAGGA
GATTGGATTCAAAATATTAACTTTAACCTTCCTGTAACAGAAGATAATGTATT
GCTACCAACAGATCCAATTGGAGGTAAAACAGGAATTAACTATACTAATATA
TTTAATACTTATGGTCCTTTAACTGCATTAAATAATGTACCACCAGTTTATCC
AAATGGTCAAATTTGGGATAAAGAATTTGATACTGACTTAAAACCAAGACT
TCATGTAAATGCACCATTTGTTTGTCAAAATAATTGTCCTGGTCAATTATTTG
TAAAAGTTGCACCTAATTTAACAAATGAATATGATCCTGATGCATCTGCTAAT
ATGTCAAGAATTGTAACTTACTCAGATTTTTGGTGGAAAGGTAAATTAGTAT
TTAAAGCTAAACTAAGAGCCTCTCATACTTGGAATCCAATTCAACAAATGA
GTATCAATGTAGATAACCAATTTAACTATGTACCAAGTAATATTGGAGGTATG

AAAATTGTATATGAAAAATCTCAACTAGCACCTAGAAAATTATAT---
