# Supplementary material for: Surveillance of Parvovirus in Free-Roaming Dogs in the Qinling Mountains and Assessment of the Risk of Cross-Species Transmission to Giant Pandas
Source: Animals (Basel). 2026 May 31;16(11):1686. doi: 10.3390/ani16111686 (PMC13255924; doi:10.3390/ani16111686)
Supplement: Supplementary file 1 [file animals-16-01686-s001.zip › Supplementary 2 Antibody Titer Grading and Exposure Criteria.pdf]

Supplementary 2 : Antibody Titer Grading and Exposure Criteria

Table S1. Antibody Titer Grading

CPV-Ab titer range	Grade	Interpretation
< 30 U	S1	Low immunity level
30 – 60 U	S2	Relatively low antibody titer
60 – 100 U	S3	Moderate antibody titer
100 – 200 U	S4	Relatively high antibody titer
200 – 500 U	S5	High antibody titer
500 – 1000 U	S6	Very high antibody titer

2. Exposure Criteria

The study period was divided into the following Intervals based on sampling time points: autumn (September – November 2024), Interval 2 (December 2024 – mid-February 2025), Interval 3 (mid-February – April 2025), and Interval 4 (May – August 2025). The interval between two consecutive sampling occasions for the same dog was defined as one Intervalal exposure assessment unit. The exposure types included in the analysis were recent exposure and clinical infection. Previous exposure was recorded but not included in statistical analyses (see Methods section of the main text).

(1) Clinical Infection

If a dog tested positive for CPV nucleic acid by qPCR at a given sampling time point, the dog was considered to have been exposed during the Interval immediately preceding that sampling time point.

(2) Recent Exposure (Seroconversion)

Recent exposure was assigned to a given Interval if any of the following criteria were met and the dog did not receive vaccination during that Interval (regardless of prior vaccination history):

- Increase in antibody grade: The antibody titer grade increased from a lower to a higher grade between two consecutive sampling occasions.
- Significant increase within the same grade (S5 only): Both pre- and post-Interval antibody titers fell within the S5 range (200–500 U), and the difference (post-Interval titer minus pre-Interval titer) was > 27.7 U. This threshold represents the statistically significant change at the 95% confidence level, calculated based on manufacturer data. (No threshold was established for S6 due to insufficient sample size.)
- Missing first sampling occasion: If blood was not collected at the previous sampling occasion because the dog was younger than 4 months of age, and the antibody titer at the subsequent sampling occasion was ≥ S2, exposure was assigned to the intervening Interval.

(3) No Exposure

No exposure was assigned to a given Interval if any of the following criteria were met:

- The dog was not vaccinated during that Interval, and the antibody titer did not increase (or the difference within the S5 grade was ≤ 27.7 U).

- The dog was vaccinated during that Interval, and there was no increase in antibody titer grade or the difference within the S5 grade was ≤ 27.7 U.

- There were consecutive missing sampling occasions between the first and last available samples for a given interval, but the difference in antibody titer between those two samples was ≤ 27.7 U. In this case, all intervening Intervals were classified as no exposure.

- There were consecutive missing sampling occasions before the first available sample, and the antibody titer at that first sampling was S1. In this case, all Intervals prior to the first sampling were classified as no exposure.

(4) Unknown Exposure Status

Exposure status was classified as unknown (and excluded from analysis) for a given Interval if any of the following criteria were met:

- There were consecutive missing sampling occasions before the first available sample, and the antibody titer at that first sampling was ≥ S2. In this case, the timing of exposure could not be determined, and all Intervals prior to the first sampling were classified as unknown.

- There were consecutive missing sampling occasions between two available samples, and the difference in antibody titer between those two samples was > 27.7 U or the antibody grade increased. In this case, the specific Interval in which exposure occurred could not be identified, and all Intervals between the two samples were classified as unknown.

- The dog was vaccinated during the interval between two consecutive sampling occasions, and the antibody titer showed either an increase in grade or an increase > 27.7 U within the S5 grade. Because vaccine-induced and natural infections could not be distinguished, the exposure status for that Interval was classified as unknown.
