# Supplementary material for: Surveillance of Parvovirus in Free-Roaming Dogs in the Qinling Mountains and Assessment of the Risk of Cross-Species Transmission to Giant Pandas
Source: Animals (Basel). 2026 May 31;16(11):1686. doi: 10.3390/ani16111686 (PMC13255924; doi:10.3390/ani16111686)
Supplement: Supplementary file 1 [file animals-16-01686-s001.zip › Supplementary 6 Results of serological statistical analysis.pdf]

Supplementary 6: Results of serological statistical analysis

Table S6.1 Sample size and exposure proportion by location

Location	Total sample size	Number exposed	Exposure proportion
Sanguanmiao	37	2	0.054
Daguping	118	52	0.441
Peripheral habitat	39	5	0.128

Table S6.2 Sample size and exposure proportion by Interval

Interval	Total sample size	Number exposed	Exposure proportion
Interval 2	50	19	0.380
Interval 4	57	15	0.263
Interval 3	52	22	0.423
Interval 1	35	3	0.086

Table S6.3 Sample size and exposure proportion by Interval and location (cross-tabulation)

Interval	Location	Total sample size	Number exposed	Exposure proportion
Interval 2	Sanguanmiao	10	1	0.100
Interval 2	Daguping	30	18	0.600
Interval 2	Peripheral habitat	10	0	0.000
Interval 4	Sanguanmiao	10	0	0.000
Interval 4	Daguping	37	11	0.297
Interval 4	Peripheral habitat	10	4	0.400
Interval 3	Sanguanmiao	10	0	0.000
Interval 3	Daguping	32	21	0.656
Interval 3	Peripheral habitat	10	1	0.100
Interval 1	Sanguanmiao	7	1	0.143
Interval 1	Daguping	19	2	0.105
Interval 1	Peripheral habitat	9	0	0.000

Table S6.4 Chi-square test results for each variable

Variable	Chi-square value	df	p-value	Significance	Note
Interval	13.1781	3	0.0043	**	
Breed	0.9717	1	0.3243	ns	
Sex	0.3575	1	0.5499	ns	
Age group	33.8814	1	0.0000	***	
Vaccination history	0.6433	1	0.4225	ns	
Location	27.0331	2	0.0000	***	

Table S6.5 Firth logistic regression results (reference: Interval 4, Daguping)

Variable	OR	CI_lower	CI_upper	p_value
(Intercept)	0.166	0.062	0.395	0.0000
Age group (juvenile vs adult)	5.367	2.449	12.481	0.0000
Interval (Interval 3 vs Interval 4)	5.419	1.870	17.054	0.0016
Interval (Interval 2 vs Interval 4)	4.384	1.503	13.771	0.0064
Interval (Interval 1 vs Interval 4)	0.354	0.060	1.520	0.1695
Location (peripheral habitat vs Daguping)	3.533	0.745	16.414	0.1094
Location (Sanguanmiao vs Daguping)	0.151	0.001	1.533	0.1245
Interval 3 × peripheral habitat	0.037	0.002	0.380	0.0050
Interval 2 × peripheral habitat	0.013	0.000	0.216	0.0014
Interval 1 × peripheral habitat	0.169	0.001	3.876	0.2834
Interval 3 × Sanguanmiao	0.350	0.002	74.820	0.6295
Interval 2 × Sanguanmiao	0.828	0.029	143.025	0.9189
Interval 1 × Sanguanmiao	17.887	0.521	3,462.994	0.1104
