# Supplementary material for: Surveillance of Parvovirus in Free-Roaming Dogs in the Qinling Mountains and Assessment of the Risk of Cross-Species Transmission to Giant Pandas
Source: Animals (Basel). 2026 May 31;16(11):1686. doi: 10.3390/ani16111686 (PMC13255924; doi:10.3390/ani16111686)
Supplement: Supplementary file 1 [file animals-16-01686-s001.zip › Supplementary 7 Comparison of Encounter Probability Between Giant Pandas and Domestic Dogs Cold vs. Warm Seasons.pdf]

Supplementary 7: Comparison of Encounter Probability Between Giant Pandas and Domestic Dogs: Cold vs. Warm Seasons

Methods This study used GPS locations of giant pandas and domestic dogs during cold and warm seasons. The study area was defined as the 95% kernel density estimation (KDE) home range of pandas. A 100-m buffer zone was created around the patrol route, and encounter probability index M was calculated for each 100-m distance band as $M = (U_{panda} * U_{dog}) / A$, where U is the weighted number of locations and A is the area of the distance band within the study area. To compare seasonal differences, a common maximum distance was determined by taking the smaller of the two seasonal maximum distances and rounding up to the nearest 100 m. Bootstrap resampling (500 iterations) was used to estimate the mean and 95% confidence intervals (CI) of M for each season and the difference $\Delta M = M_{warm} - M_{cold}$. Paired t-test and Wilcoxon signed-rank test were applied to assess overall seasonal differences.
Overall Seasonal Differences Paired t-test: $t = -2.345$, $df = 29$, $p = 0.023$ Wilcoxon signed-rank test: $V = 15$, $p = 0.031$ The results indicate a significant difference in encounter probability between cold and warm seasons ($p < 0.05$).

Table S7.1. Encounter probability index M (95% CI) for cold and warm seasons, and the difference ΔM (95% CI) by distance band.

Distance band	Area_cold (m^2)	Area_warm (m^2)	Cold M mean	Cold M lower	Cold M upper	Warm M mean	Warm M lower	Warm M upper	ΔM mean	ΔM lower	ΔM upper	significant
0-100 m	2,087,242	990,696	0.5191	0.4433	0.5924	0.0003	0.0000	0.0009	-0.5188	-0.5920	-0.4431	significant
100-200 m	2,027,380	969,111	0.0608	0.0413	0.0815	0.0010	0.0002	0.0021	-0.0598	-0.0805	-0.0400	significant
200-300 m	1,966,831	944,848	0.0523	0.0374	0.0694	0.0003	0.0000	0.0010	-0.0520	-0.0691	-0.0369	significant
300-400 m	1,999,508	932,484	0.0180	0.0104	0.0275	0.0047	0.0000	0.0126	-0.0133	-0.0240	-0.0027	significant
400-500 m	2,035,237	917,983	0.0152	0.0066	0.0249	0.0000	0.0000	0.0000	-0.0152	-0.0249	-0.0066	significant
500-600 m	1,993,890	889,075	0.0071	0.0031	0.0115	0.0000	0.0000	0.0000	-0.0071	-0.0115	-0.0031	significant
600-700 m	1,950,001	815,963	0.0069	0.0031	0.0119	0.0000	0.0000	0.0000	-0.0069	-0.0119	-0.0031	significant
700-800 m	1,857,022	737,707	0.0176	0.0090	0.0274	0.0000	0.0000	0.0000	-0.0176	-0.0274	-0.0090	significant
800-900 m	1,730,759	661,899	0.0044	0.0012	0.0083	0.0000	0.0000	0.0000	-0.0044	-0.0083	-0.0012	significant
900-1000 m	1,585,986	647,428	0.0044	0.0019	0.0079	0.0000	0.0000	0.0000	-0.0044	-0.0079	-0.0019	significant

Distance band	Area_cold (m²)	Area_warm (m²)	Cold M mean	Cold M lower	Cold M upper	Warm M mean	Warm M lower	Warm M upper	ΔM mean	ΔM lower	ΔM upper	significant
1000-1100 m	1,485,168	711,638	0.0072	0.0028	0.0116	0.0000	0.0000	0.0000	-0.0072	-0.0116	-0.0028	significant
1100-1200 m	1,343,342	759,998	0.0093	0.0048	0.0159	0.0000	0.0000	0.0000	-0.0093	-0.0159	-0.0048	significant
1200-1300 m	1,283,739	799,581	0.0148	0.0066	0.0239	0.0000	0.0000	0.0000	-0.0148	-0.0239	-0.0066	significant
1300-1400 m	1,217,254	837,501	0.0029	0.0010	0.0055	0.0000	0.0000	0.0000	-0.0029	-0.0055	-0.0010	significant
1400-1500 m	1,122,361	891,460	0.0017	0.0006	0.0031	0.0000	0.0000	0.0000	-0.0017	-0.0031	-0.0006	significant
1500-1600 m	1,097,136	912,062	0.0006	0.0002	0.0012	0.0000	0.0000	0.0000	-0.0006	-0.0012	-0.0002	significant
1600-1700 m	1,007,961	914,864	0.0001	0.0000	0.0003	0.0000	0.0000	0.0000	-0.0001	-0.0003	0.0000	significant
1700-1800 m	905,453	918,633	0.0002	0.0000	0.0004	0.0000	0.0000	0.0000	-0.0002	-0.0004	0.0000	significant
1800-1900 m	781,114	922,940	0.0012	0.0005	0.0022	0.0000	0.0000	0.0000	-0.0012	-0.0022	-0.0005	significant
1900-2000 m	657,905	926,851	0.0028	0.0012	0.0048	0.0000	0.0000	0.0000	-0.0028	-0.0048	-0.0012	significant

Distance band	Area_cold (m²)	Area_warm (m²)	Cold M mean	Cold M lower	Cold M upper	Warm M mean	Warm M lower	Warm M upper	ΔM mean	ΔM lower	ΔM upper	significant
2000-2100 m	557,788	932,397	0.0017	0.0004	0.0033	0.0000	0.0000	0.0000	-0.0017	-0.0033	-0.0004	significant
2100-2200 m	442,500	942,449	0.0017	0.0000	0.0039	0.0000	0.0000	0.0000	-0.0017	-0.0039	0.0000	non-significant
2200-2300 m	326,637	950,174	0.0000	0.0000	0.0000	0.0000	0.0000	0.0000	0.0000	0.0000	0.0000	non-significant
2300-2400 m	266,341	956,805	0.0000	0.0000	0.0000	0.0000	0.0000	0.0000	0.0000	0.0000	0.0000	non-significant
2400-2500 m	263,185	962,209	0.0000	0.0000	0.0000	0.0000	0.0000	0.0000	0.0000	0.0000	0.0000	non-significant
2500-2600 m	259,741	962,337	0.0000	0.0000	0.0000	0.0000	0.0000	0.0000	0.0000	0.0000	0.0000	non-significant
2600-2700 m	252,588	961,394	0.0000	0.0000	0.0000	0.0000	0.0000	0.0000	0.0000	0.0000	0.0000	non-significant
2700-2800 m	244,624	956,719	0.0000	0.0000	0.0000	0.0000	0.0000	0.0000	0.0000	0.0000	0.0000	non-significant
2800-2900 m	234,332	948,730	0.0000	0.0000	0.0000	0.0000	0.0000	0.0000	0.0000	0.0000	0.0000	non-significant
2900-3000 m	221,875	937,910	0.0000	0.0000	0.0000	0.0000	0.0000	0.0000	0.0000	0.0000	0.0000	non-significant

Table S7.2. Jacobs index and related metrics for cold season.

Distance band	Area (m²)	Actual panda locations	Actual dog locations	Panda selection index (D)	Dog selection index (D)	Encounter probability index (M)	Normalized M
0-100 m	2,087,242	684.42	1,143	0.5523	0.8112	0.3748	0.6585
100-200 m	2,027,380	453.28	224	0.3750	0.1261	0.0501	0.0880
200-300 m	1,966,831	278.45	296	0.1396	0.2873	0.0419	0.0736
300-400 m	1,999,508	248.41	118	0.0702	-0.2024	0.0147	0.0258
400-500 m	2,035,237	272.47	75	0.1101	-0.4209	0.0100	0.0176
500-600 m	1,993,890	132.72	72	-0.2525	-0.4291	0.0048	0.0084
600-700 m	1,950,001	143.60	64	-0.2026	-0.4678	0.0047	0.0083
700-800 m	1,857,022	208.76	100	0.0168	-0.2468	0.0112	0.0198
800-900 m	1,730,759	109.23	44	-0.2765	-0.5615	0.0028	0.0049
900-1000 m	1,585,986	75.14	76	-0.4051	-0.3007	0.0036	0.0063
1000-1100 m	1,485,168	124.91	74	-0.1336	-0.2818	0.0062	0.0109
1100-1200 m	1,343,342	116.52	92	-0.1174	-0.1246	0.0080	0.0140
1200-1300 m	1,283,739	213.77	76	0.2192	-0.1973	0.0127	0.0222
1300-1400 m	1,217,254	57.03	53	-0.4072	-0.3422	0.0025	0.0044
1400-1500 m	1,122,361	31.17	55	-0.6017	-0.2873	0.0015	0.0027
1500-1600 m	1,097,136	15.27	76	-0.7791	-0.1180	0.0011	0.0019

Distance band	Area (m²)	Actual panda locations	Actual dog locations	Panda selection index (D)	Dog selection index (D)	Encounter probability index (M)	Normalized M
1600-1700 m	1,007,961	3.10	72	-0.9466	-0.1022	0.0002	0.0004
1700-1800 m	905,453	8.27	40	-0.8487	-0.3335	0.0004	0.0006
1800-1900 m	781,114	25.85	75	-0.5397	0.0492	0.0025	0.0044
1900-2000 m	657,905	55.32	79	-0.1308	0.1622	0.0066	0.0117
2000-2100 m	557,788	61.01	36	0.0022	-0.1515	0.0039	0.0069
2100-2200 m	442,500	91.51	24	0.3155	-0.2355	0.0050	0.0087
2200-2300 m	326,637	30.50	0	-0.0774	-1.0000	0.0000	0.0000
2300-2400 m	266,341	119.43	0	0.6170	-1.0000	0.0000	0.0000
2400-2500 m	263,185	71.35	0	0.4316	-1.0000	0.0000	0.0000
2500-2600 m	259,741	53.25	0	0.3092	-1.0000	0.0000	0.0000
2600-2700 m	252,588	14.48	0	-0.3120	-1.0000	0.0000	0.0000
2700-2800 m	244,624	0.52	0	-0.9622	-1.0000	0.0000	0.0000
2800-2900 m	234,332	1.03	0	-0.9226	-1.0000	0.0000	0.0000
2900-3000 m	221,875	0.52	0	-0.9584	-1.0000	0.0000	0.0000
3000-3100 m	204,545	0.00	0	-1.0000	-1.0000	0.0000	0.0000
Total	33,409,443	3,701.31	2,964				

Table S7.3. Jacobs index and related metrics for warm season.

Distance band	Area (m²)	Actual panda locations	Actual dog locations	Panda selection index (D)	Dog selection index (D)	Encounter probability index (M)	Normalized M
0-100 m	990,696	2.96	175	-0.9434	0.9790	0.0005	0.1294
100-200 m	969,111	44.06	35	-0.3810	0.7132	0.0016	0.3930
200-300 m	944,848	33.17	14	-0.4866	0.3794	0.0005	0.1214
300-400 m	932,484	96.05	14	0.0160	0.3852	0.0014	0.3562
400-500 m	917,983	137.29	0	0.2059	-1.0000	0.0000	0.0000
500-600 m	889,075	80.48	0	-0.0502	-1.0000	0.0000	0.0000
600-700 m	815,963	102.64	0	0.1181	-1.0000	0.0000	0.0000
700-800 m	737,707	28.81	0	-0.4429	-1.0000	0.0000	0.0000
800-900 m	661,899	85.40	0	0.1303	-1.0000	0.0000	0.0000
900-1000 m	647,428	14.41	0	-0.6398	-1.0000	0.0000	0.0000
1000-1100 m	711,638	32.19	0	-0.3813	-1.0000	0.0000	0.0000
1100-1200 m	759,998	34.15	0	-0.3845	-1.0000	0.0000	0.0000
1200-1300 m	799,581	62.61	0	-0.1235	-1.0000	0.0000	0.0000
1300-1400 m	837,501	29.65	0	-0.4827	-1.0000	0.0000	0.0000
1400-1500 m	891,460	133.90	0	0.2078	-1.0000	0.0000	0.0000
1500-1600 m	912,062	96.72	0	0.0310	-1.0000	0.0000	0.0000

Distance band	Area (m²)	Actual panda locations	Actual dog locations	Panda selection index (D)	Dog selection index (D)	Encounter probability index (M)	Normalized M
1600-1700 m	914,864	95.67	0	0.0238	-1.0000	0.0000	0.0000
1700-1800 m	918,633	36.71	0	-0.4349	-1.0000	0.0000	0.0000
1800-1900 m	922,940	113.87	0	0.1086	-1.0000	0.0000	0.0000
1900-2000 m	926,851	95.92	0	0.0184	-1.0000	0.0000	0.0000
2000-2100 m	932,397	115.34	0	0.1100	-1.0000	0.0000	0.0000
2100-2200 m	942,449	223.88	0	0.4244	-1.0000	0.0000	0.0000
2200-2300 m	950,174	349.24	0	0.5986	-1.0000	0.0000	0.0000
2300-2400 m	956,805	163.59	0	0.2721	-1.0000	0.0000	0.0000
2400-2500 m	962,209	111.52	0	0.0767	-1.0000	0.0000	0.0000
2500-2600 m	962,337	155.02	0	0.2431	-1.0000	0.0000	0.0000
2600-2700 m	961,394	29.12	0	-0.5415	-1.0000	0.0000	0.0000
2700-2800 m	956,719	52.49	0	-0.2966	-1.0000	0.0000	0.0000
2800-2900 m	948,730	83.33	0	-0.0657	-1.0000	0.0000	0.0000
2900-3000 m	937,910	146.26	0	0.2268	-1.0000	0.0000	0.0000
3000-3100 m	920,467	136.21	0	0.2006	-1.0000	0.0000	0.0000
3100-3200 m	898,788	81.00	0	-0.0525	-1.0000	0.0000	0.0000

Distance band	Area (m²)	Actual panda locations	Actual dog locations	Panda selection index (D)	Dog selection index (D)	Encounter probability index (M)	Normalized M
3200-3300 m	872,681	40.11	0	-0.3755	-1.0000	0.0000	0.0000
3300-3400 m	838,057	28.62	0	-0.4965	-1.0000	0.0000	0.0000
3400-3500 m	797,938	73.18	0	-0.0434	-1.0000	0.0000	0.0000
3500-3600 m	699,307	60.52	0	-0.0728	-1.0000	0.0000	0.0000
3600-3700 m	512,306	140.27	0	0.4759	-1.0000	0.0000	0.0000
3700-3800 m	449,173	28.62	0	-0.2232	-1.0000	0.0000	0.0000
3800-3900 m	403,123	28.62	0	-0.1706	-1.0000	0.0000	0.0000
3900-4000 m	352,174	31.90	0	-0.0491	-1.0000	0.0000	0.0000
4000-4100 m	273,756	0.00	0	-1.0000	-1.0000	0.0000	0.0000
4100-4200 m	199,943	14.31	0	-0.1658	-1.0000	0.0000	0.0000
4200-4300 m	181,310	0.00	0	-1.0000	-1.0000	0.0000	0.0000
Total	34,012,869	3,449.79	238				

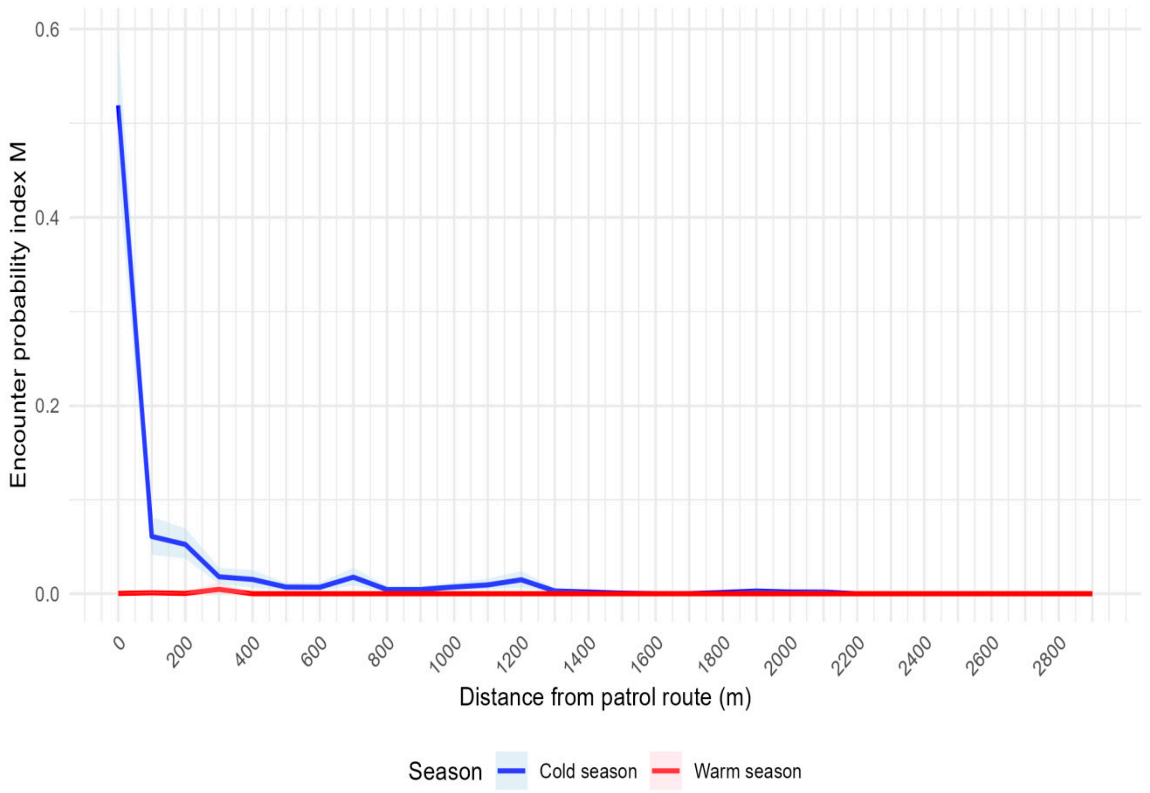

Figure S7.1. Comparison of encounter probability between cold and warm seasons. Shaded areas represent 95% bootstrap confidence intervals.
